# Activity‐Directed Synthesis of Inhibitors of the p53/*h*DM2 Protein–Protein Interaction

**DOI:** 10.1002/chem.202002153

**Published:** 2020-08-04

**Authors:** Adam I. Green, Fruzsina Hobor, Christopher P. Tinworth, Stuart Warriner, Andrew J. Wilson, Adam Nelson

**Affiliations:** ^1^ School of Chemistry University of Leeds Woodhouse Lane Leeds LS2 9JT UK; ^2^ Astbury Centre for Structural Molecular Biology University of Leeds Woodhouse Lane Leeds LS2 9JT UK; ^3^ School of Molecular and Cellular Biology University of Leeds Woodhouse Lane Leeds LS2 9JT UK; ^4^ GlaxoSmithKline Medicines Research Centre Stevenage SG1 2NY UK

**Keywords:** lead discovery, metal carbenoids, molecular diversity, protein–protein interactions, scaffold hopping

## Abstract

Protein–protein interactions (PPIs) provide a rich source of potential targets for drug discovery and biomedical science research. However, the identification of structural‐diverse starting points for discovery of PPI inhibitors remains a significant challenge. Activity‐directed synthesis (ADS), a function‐driven discovery approach, was harnessed in the discovery of the p53/*h*DM2 PPI. Over two rounds of ADS, 346 microscale reactions were performed, with prioritisation on the basis of the activity of the resulting product mixtures. Four distinct and novel series of PPI inhibitors were discovered that, through biophysical characterisation, were shown to have promising ligand efficiencies. It was thus shown that ADS can facilitate ligand discovery for a target that does not have a defined small‐molecule binding site, and can provide distinctive starting points for the discovery of PPI inhibitors.

## Graphical abstract

**Activity‐directed synthesis** (ADS) was harnessed in the discovery of the p53/*h*DM2 protein–protein interaction. Four novel series of PPI inhibitors were discovered that had promising ligand efficiencies. It was demonstrated that ADS can enable scaffold hopping, and facilitate lead discovery for targets lacking defined small‐molecule binding sites.

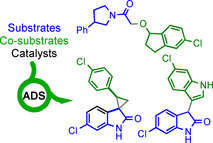

Protein–protein interactions (PPIs) are ubiquitous in cellular signalling mechanisms, and provide a rich source of potential targets for drug discovery.^1^ Aberrant PPIs have prompted the discovery of PPI inhibitors including small molecules, peptides and peptidomimetics.^2^ A recent success is the BCL‐2 inhibitor venetoclax (ABT‐199),^3^ which was discovered using a fragment‐based discovery approach, and is now used clinically to treat chronic lymphocytic leukaemia and small lymphocytic lymphoma.

Although they occur over a large surface area,2a the binding affinity in PPIs is often dominated by a small number of hotspot residues^4^ (or hot regions) which can inform inhibitor design. For example, the p53/*h*DM2 PPI inhibitors RG7112, MI‐77301 and AM‐8735 (Figure 1) all target *h*DM2 subpockets that are addressed by three hotspot residues on p53 (F19, W23 and L26).^5^ Deconstruction of the nutlin RG7112 has provided an insight into which combinations of groups are necessary to bind *h*DM2.^6^ Some of the substructures that target two *h*DM2 subpockets (F19/W23 or W23/L26) such as **1** and **2**, have detectable binding (by NMR) and could have been plausible starting points for the discovery of RG7112 using a fragment‐based approach.

**Figure 1:**
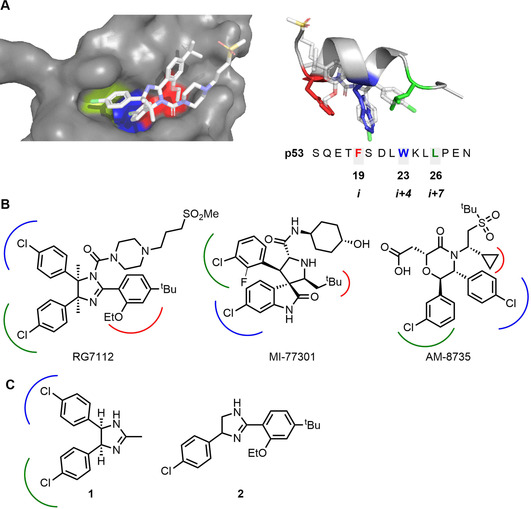
p53/*h*DM2 PPI inhibitors that target *h*DM2 subpockets that are addressed by hotspot residues on p53: F19, W23 and L26. Panel A: Structure of *h*DM2 in complex with RG7112 (PDB: 4IPF)^6^ and overlay of RG7112 with p53 transactivation domain (PDB: 1YCR);5e the subpockets targeted by p53 hotspot residues F19 (red), W23 (blue) and L26 (green) are shown. Panel B: Known *h*DM2 antagonists that target *h*DM2 subpockets. Panel C: Substructures of RG7112 that target two *h*DM2 subpockets and have detectable binding (by NMR) to *h*DM2.^5^, ^6^

We recently introduced activity‐directed synthesis, a function‐driven approach for the discovery of bioactive small molecules. The approach deliberately harnesses arrays of reactions that have many alternative outcomes, with promising reactions being prioritised based on the function of the corresponding product mixtures. Crude reaction mixtures are also screened in other discovery approaches^7^ including synthetic fermentation^8^ in which specific designed molecules are also not targeted. We have demonstrated that metal‐catalysed carbenoid chemistry^9^ may be successfully harnessed in the activity‐directed discovery of novel androgen receptor agonists.^10^ To date, the approach has only be applied to androgen receptor, a protein target with a binding site that has evolved to bind a small‐molecule ligand. In this Communication, we demonstrate that activity‐directed synthesis can also be exploited in the discovery of diverse inhibitors of a more challenging target: the p53/*h*DM2 PPI.

We designed 7 diazo substrates (**D1–7**) and 10 co‐substrates (**S1–10**), many of which contain groups with the potential to, or have been demonstrated to, mimic p53 hotspot residues: for example, phenyl, chlorophenyl and branched/cyclic/fluorinated alkyl groups.^5^, ^10^, ^11^ In addition, the co‐substrates all contained at least one functional group with precedented reactivity towards metal carbenoids such as alkene, benzylic C−H, hydroxyl, nitrile and indole groups.^9^, ^12^ We envisaged that some combinations of diazo substrates and co‐substrates may react to yield products that can inhibit the p53/*h*DM2 PPI by targeting multiple *h*DM2 subpockets (Figure 2).

**Figure 2:**
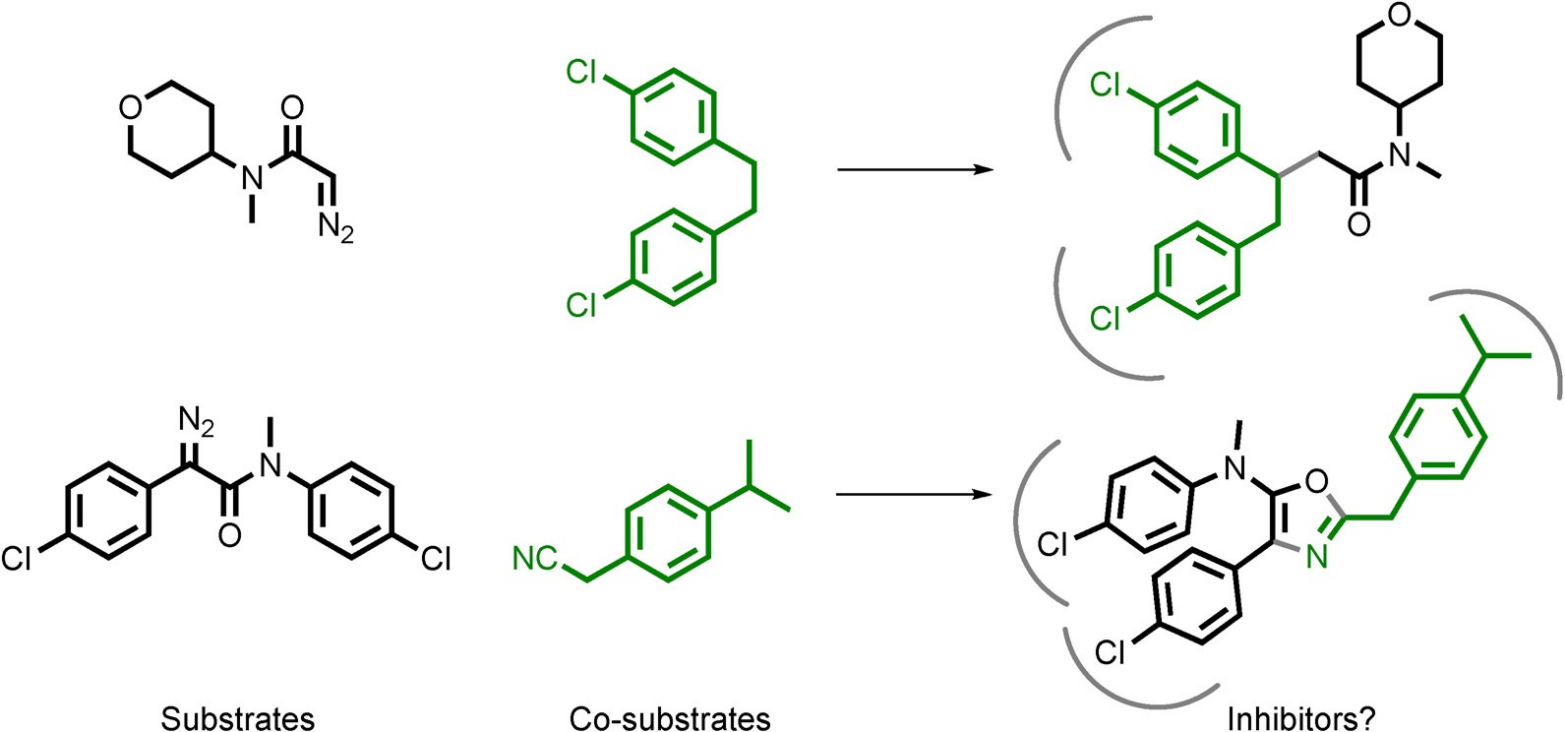
Potential outcomes of reactions between substrates (black) and co‐substrates (green). In ADS, reactions are prioritised based on the activity of the reaction mixtures.

Our first array of 154 reactions involved all combinations of seven diazo substrates (**D1–7**), eleven co‐substrates (**S1–10** and no co‐substrate), and two catalysts (Rh_2_piv_4_ and Rh_2_pfb_4_) (Figure 3). The catalysts were selected on the basis of their solubility in organic solvents and their complementary reactivity.^12^ Crucially, we had shown that none of the reaction components (diazo substrates: 20 μm; co‐substrates: 100 μm; catalysts: 200 nm) were active in our assay at the relevant screening concentration (see Supporting Information, Section 5.5). The reactions were assembled from stock solutions using multi‐channel pipettes and were performed in microscale vials in 96‐well plate format. Each reaction involved a diazo substrate (100 mm final concentration), a co‐substrate (500 mm) and 1 mol % catalyst (1 mm) in dichloromethane (total reaction volume: 100 μL). After 24 h, the crude reaction mixtures were scavenged to remove metal contaminants, evaporated and screened in duplicate using a fluorescence anisotropy assay for displacement of a p53 tracer peptide from *h*DM2 (total concentration of products based on the limiting diazo reactant: 20 μm in 1 % DMSO in pH 7.5 aqueous phosphate buffer).^13^ Intermolecular reaction products were observed by LC/MS for ≈75 % of the reactions (see Supporting Information, Section 7), demonstrating that most reactions had been productive. Six reaction mixtures derived from the diazo **D3** displayed promising activity (>35 % activity relative to 10 μm Nutlin‐3a), including with no co‐substrate, suggesting that an active product had likely been formed through an intramolecular reaction. The Rh_2_piv_4_‐catalysed reaction of the diazo substrate **D3** was therefore scaled up and was found to yield both the oxindole **P3** and the α‐keto amide **P4** (entry 3, Table 1). In addition, six other reaction mixtures displayed promising activity, three of which were found by LC/MS to contain an intermolecular reaction product.

**Figure 3:**
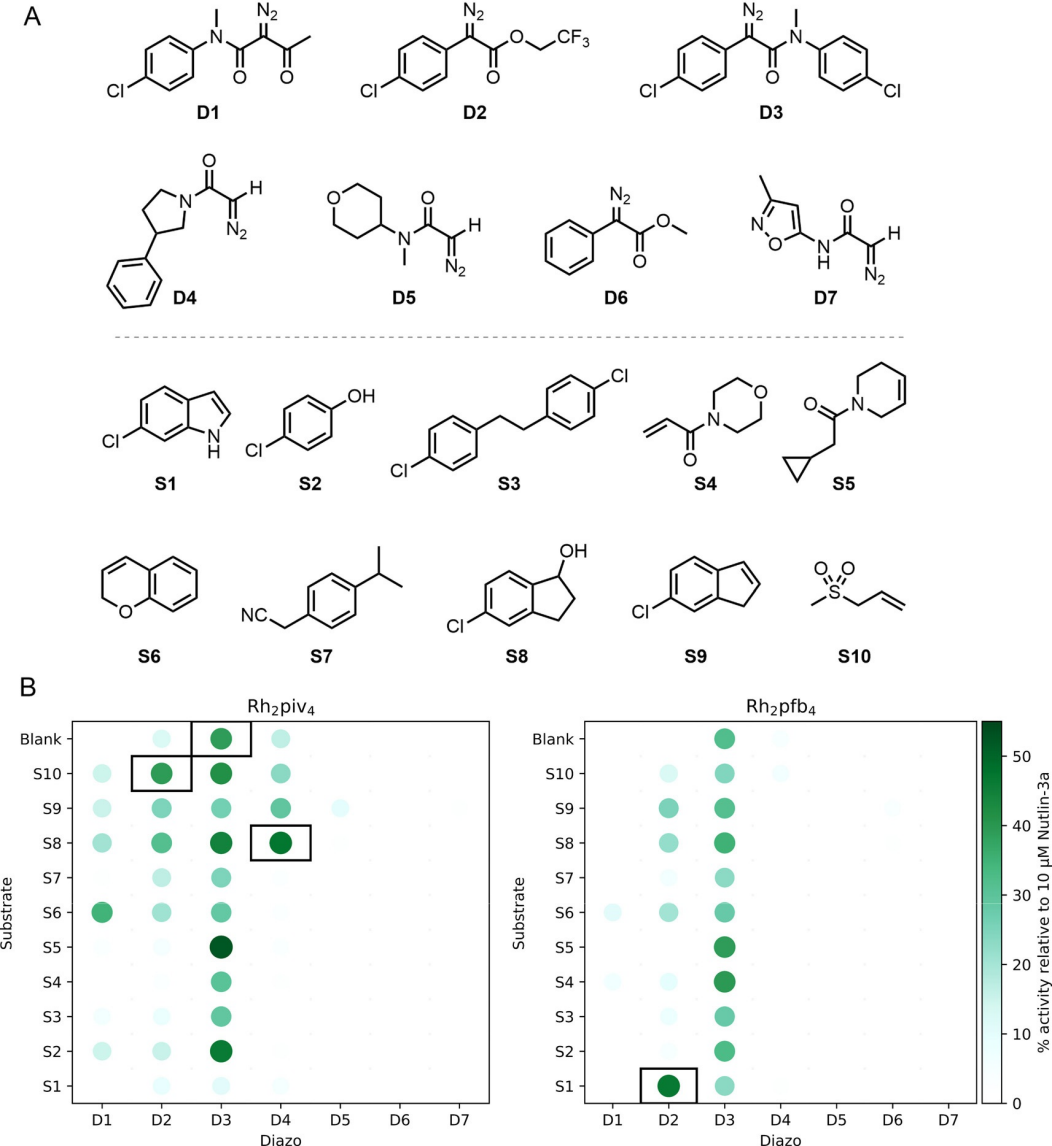
Round one of activity‐directed synthesis. Panel A: Diazo substrates and co‐substrates used. Panel B: Activities of product mixtures, screened in duplicate at 20 μm total product concentration, relative to 10 μm Nutlin‐3a (see Supporting Information for details). Products of reactions catalysed by Rh_2_piv_4_ (left) and Rh_2_pfb_4_ (right) that displayed promising activity (>35 % activity relative to 10 μm Nutlin‐3a), and for which the expected product mass was detected by LC‐MS (see text), are shown (black boxes).

**Table 1:** Yield and activities of the purified products derived from hit reaction mixtures.

Entry	Round^[a]^	Diazo	Substrate	Catalyst	Product (Yield^[b]^)	Fluorescence anisotropy^[c]^ IC_50_ [μm]	NMR Binding^[d]^ (*K*_d_/μm)	
1	1	**D2**	**S1**	Rh_2_pfb_4_	**P1** (14 %)	–^[e]^	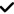	–^[f,h]^
2	1	**D4**	**S8**	Rh_2_piv_4_	**P2** (78 %)	15.0±0.1	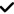	(34±13)
3	1	**D3**	blank	Rh_2_piv_4_	**P3** (4 %)	>100	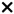	–
					**P4** (4 %)	>30	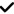	(35±16)
4	2	**D8**	**S17**	Rh_2_pfb_4_	**P5** (58 %)	≈10^[g]^	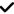	(<10^[f]^)
5	2	**D8**	**S1**	Rh_2_piv_4_	**P6** (53 %)	0.94±0.03	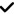	(<20^[f]^)
6	2	**D8**	**S20**	Rh_2_piv_4_	**P7** (14 %)	>160	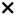	–
					Nutlin‐3a	0.095±0.02	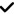	(<10^[f]^)

[a] Diazo substrate (1 equiv), co‐substrate (5 equiv), catalyst (1 mol %), dichloromethane solvent. [b] Isolated yield of purified product. [c] 50 nm *h*DM2_17‐25_, 25 nm fluorescein‐labelled p53 tracer, 0.02 mg mL^−1^ bovine serum albumin, 200 mm NaCl, 1 % DMSO in 40 mm pH 7.5 phosphate buffer; results obtained after 24 h incubation. [d] ^1^H–^15^N HSQC NMR experiment with 50 μm ^15^N‐labelled *h*DM2_17‐125_, 1 mm DTT, 1 % DMSO and 2.5 % glycerol in 100 mm pH 7.5 phosphate buffer. [e] Behaviour not consistent with direct competition of the peptide tracer for *h*DM2 binding. [f] Accurate determination not possible because *K*_d_ was much lower than the protein concentration. [g] Accurate determination not possible due to limited solubility. [h] Accurate determination not possible due to intermediate and slow exchange of key reporter peaks.

We designed a second reaction array based on the intermolecular hit reactions that had been identified in the first round (Figure 4). New diazo substrates (**D8** and **D9**) and co‐substrates (**S11**–**21**) were inspired by components used in these hit reactions, whilst **D8** was also an analogue of the product of an intramolecular hit reaction (**P3**). The new diazo **D10** was expected to mirror the observed reactivity of **D7** in the first reaction array where all but three reactions had been observed by LC/MS to yield intermolecular reaction products. In total, the array comprised 196 reactions in which all combinations of the six diazo substrates, the sixteen co‐substrates (including no co‐substrate) and two catalysts (Rh_2_piv_4_ and Rh_2_pfb_4_) were investigated. Once more, after 24 h, the reactions were scavenged and evaporated, and the crude reaction mixtures were screened in our fluorescence anisotropy assay. Six additional reaction mixtures displayed promising biological activity and were identified.

**Figure 4:**
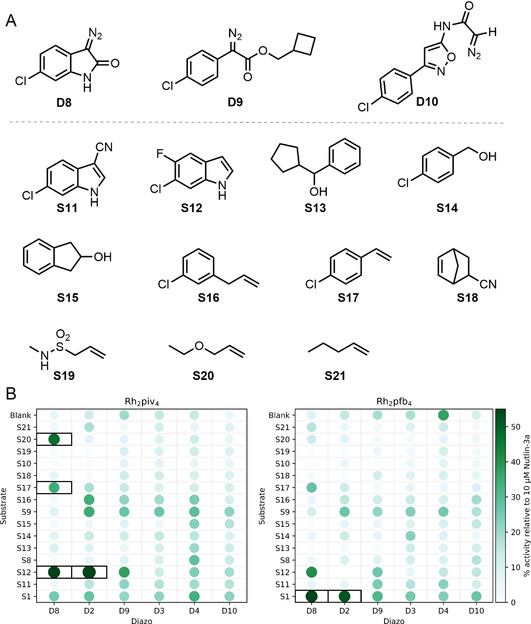
Round two of activity‐directed synthesis. Panel A: Additional diazo substrates and co‐substrates used. Panel B: Activities of product mixtures, screened in duplicate at 20 μm total product concentration, relative to 10 μm Nutlin‐3a (see Supporting Information for details). Reaction mixtures that displayed promising activity (>35 % activity relative to 10 μm nutlin‐3a) are shown (black boxes).

In addition to the intramolecular hit reaction of the diazo substrate **D3** that had previously been identified (entry 3, Table 1), we scaled up further hit reactions from both rounds of activity‐directed synthesis (entries 2–6). The reactions were typically repeated on 50‐fold larger scale, and the products were purified by column chromatography. The purified products were structurally elucidated, and were found to stem from a range of reaction types: insertion into indolyl C−H bonds (→**P1** and **P6**, entries 2 and 5); insertion into alcohol O−H bonds (→**P2**, entry 2); and cyclopropanation (→**P5** and **P7**, entries 4 and 6).

The purified products (Figure 5) were characterised in our fluorescence anisotropy assay and by ^1^H–^15^N HSQC NMR spectroscopy (Table 1; Figure 6). Titration of five of the products (**P1**, **P2**, **P4**, **P5** and **P6**) into 50 μm ^15^N‐labelled *h*DM2_17‐125_ resulted in specific concentration‐dependent perturbation of the chemical shifts of key residues that was consistent with protein‐ligand interaction.^14^ To assess selectivity, products were similarly titrated into ^15^N‐labelled MCL‐1, and no significant chemical shift perturbation was observed (see Supporting Information, Section 6.2). The concentration‐dependent fluorescence anisotropy observed with four of these ligands (**P2**, **P4**, **P5** and **P6**) was consistent with displacement of the peptide tracer from *h*DM2. In the case of **P1**, however, the anisotropy of the free tracer was not observed at high ligand concentration which may be symptomatic of more complex binding behaviour.^15^ The activity of **P7** was not validated using either biophysical method, suggesting that the *h*DM2‐binding reaction product had not been found in this case. The chemical shift perturbations induced by **P2**, **P4**, **P5** and **P6** were mapped onto the structure of *h*DM2 and were found to be consistent with ligand binding to the p53 binding cleft (Figure 6 and Supporting Information, Section 6). For all four of these ligands, docking studies suggested that the aromatic substituents (Figure 6 and Supporting Information, Section 8.1) may target the same pair of *h*DM2 subpockets as those in optimised inhibitors (Figure 1 and Supporting Information, Sections 8.1 and 8.2). We note, therefore, that ADS has enabled experimental scaffold‐hopping:^16^ that is, it has resulted in the discovery of ligands in which a common pharmacophore is displayed in the context of alternative scaffolds.

**Figure 5:**
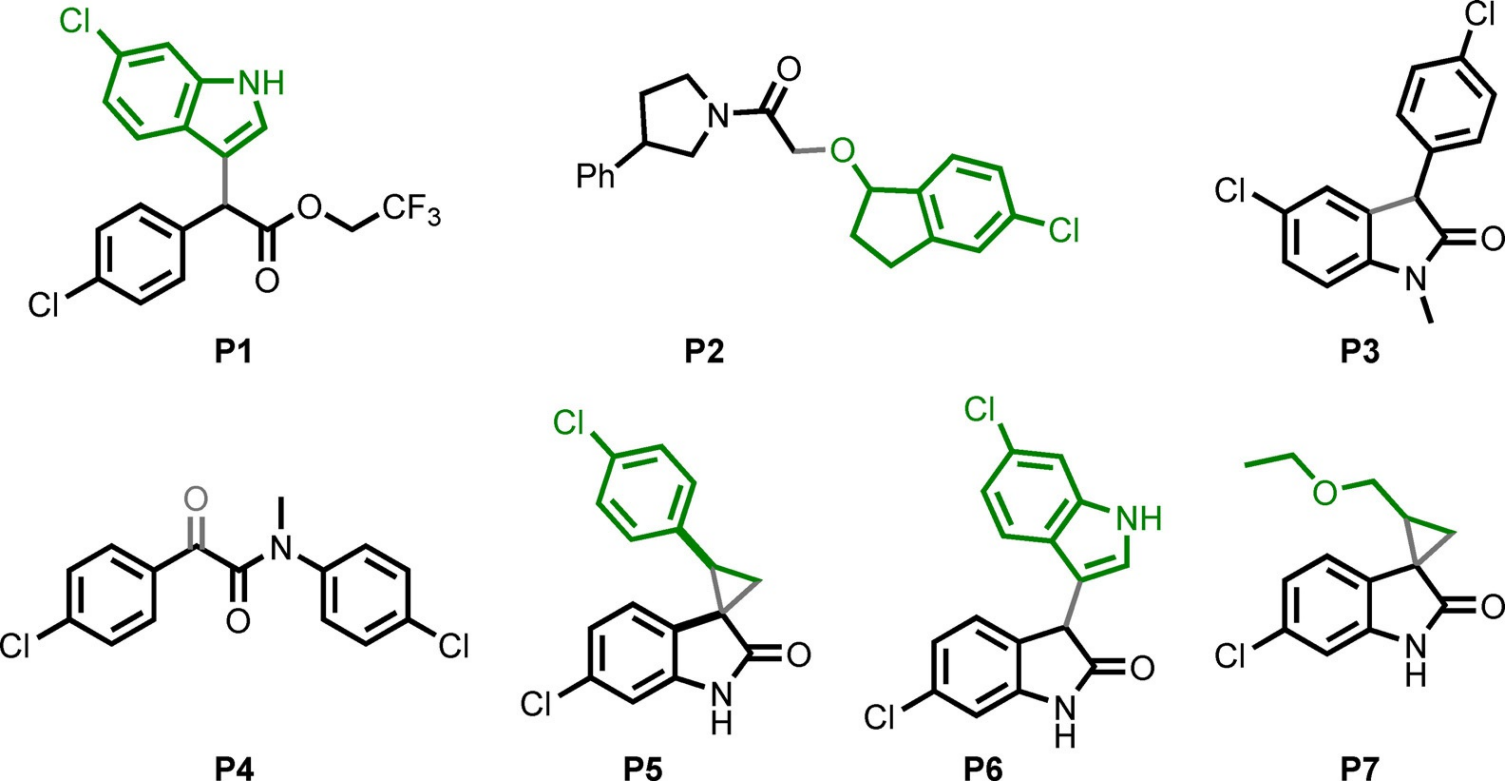
Purified products of reactions that were scaled up (see Table 1 for details). The provenance of structures from diazo substrates (black) and co‐substrates (green) is shown.

**Figure 6:**
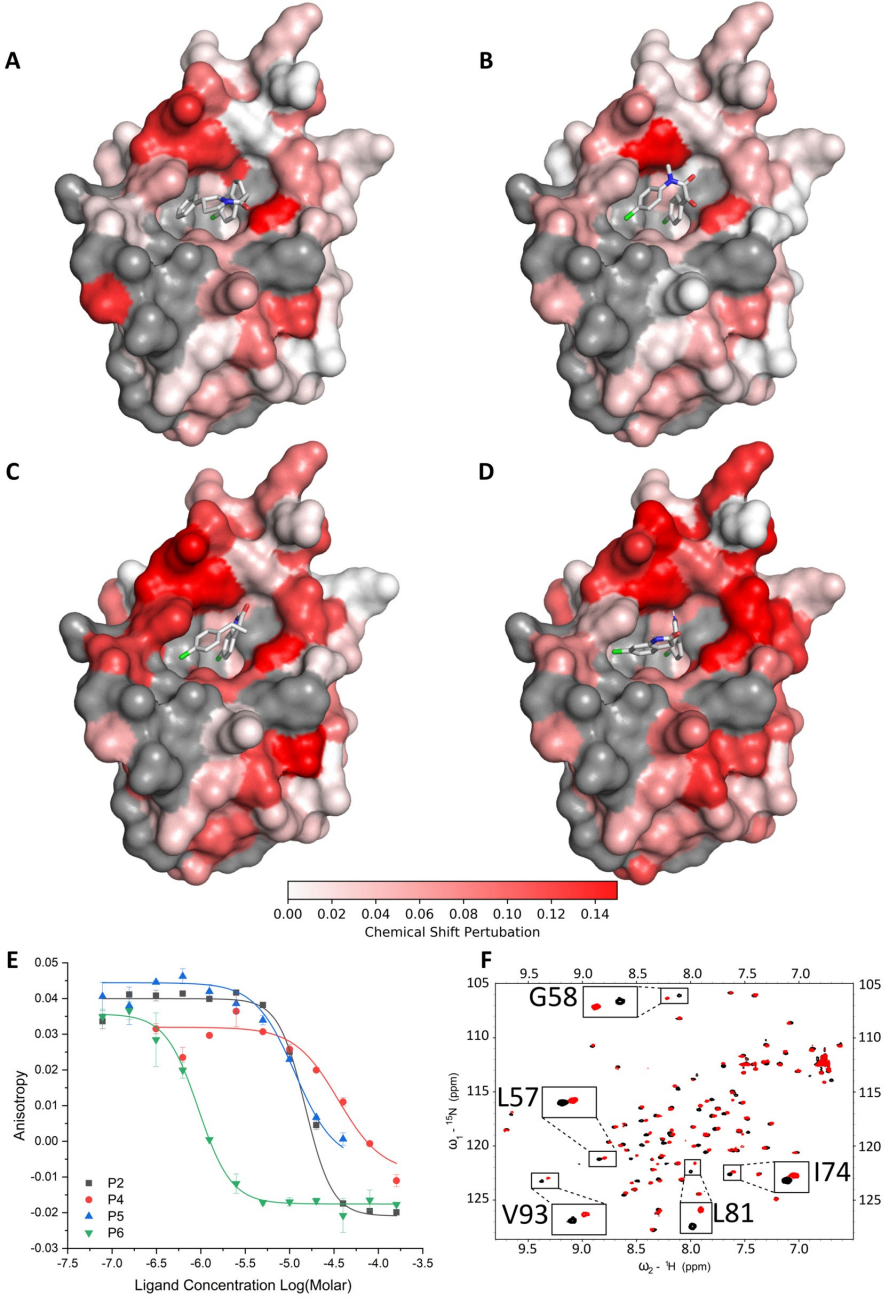
Characterisation of inhibitors of the p53/*h*DM2 PPI. Panels A–D: ^15^N‐H HSQC chemical shift perturbation of assigned peaks for 50 μm ^15^N‐labelled *h*DM2 on addition of ligand (A: 200 μm **P2**; B: 300 μm **P4**; C: 100 μm **P5**; D: 200 μm **P6**; Supporting Information, Section 6.1: nutlin‐3a) (unassigned residues, grey). The ligands were docked into *h*DM2 [PDB: 6Q9H] (see Supporting Information, Section 8.1). Panel E: Dose‐response fluorescence anisotropy competition assay. Panel F: ^1^H‐^15^N HSQC NMR spectrum in the absence (black) and presence (red) of 200 μm **P2**.

We assessed the similarity of the PPI inhibitors **P2**, **P4**, **P5** and **P6** by pairwise comparison of their Morgan molecular fingerprints (see Supporting Information, Section 8.3).^17^ In each case, the Tanimoto similarity index was low (between 0.28 and 0.46); it is remarkable that such dissimilar molecules were both prepared and identified as PPI inhibitors as part of the same discovery workflow. We also compared the inhibitors with 1314 *h*DM2 ligands extracted from the ChEMBL database.18a The similarity of each ligand with its nearest neighbour11a, 18 in ChEMBL was also low (between 0.37 and 0.61), demonstrating that activity‐directed synthesis had enabled the discovery of four distinct and novel chemotype series.

Finally, to demonstrate the value of ADS in the generation of PPI inhibitor series, we performed a limited SAR study for three of the chemotypes. Eight analogues were prepared by Rh‐catalysed reactions of the relevant diazo substrates with appropriate co‐substrates and allowed key structural features to be identified (see Supporting Information, Section 5.2). Both aryl rings in **P2**, and the substituted phenyl ring in **P5**, were found to be essential, which is consistent these groups targeting *h*DM2 subpockets.

In summary, we have demonstrated that ADS can drive the discovery of novel inhibitors of the p53/*h*DM2 PPI. Over two rounds of ADS, 10 diazo substrates and 21 co‐substrates were used, many incorporating groups intended to target *h*DM2 subpockets. In total, 346 microscale reactions were performed that resulted in, for example, ligand rigidification (via cyclisation) or fragment linkage (via reaction between pairs of substrates). By drawing on knowledge of substituents found in known ligands, it was possible to discover diverse ligands based on alternative scaffolds. In total, four distinct and novel series of PPI inhibitors were discovered whose ligand efficiency (LE, ranging from 0.28 to >0.4) compared well with those of deconstructed RG7112 analogues^6^ that target pairs of *h*DM2 subpockets (**1**: LE=0.31; **2**: not determinable). We have shown that ADS can facilitate ligand discovery for a target that, unlike androgen receptor, does not have a defined small‐molecule binding site. We conclude that ADS is a useful addition to the lead generation toolkit, and can provide distinctive starting points for the discovery of PPI inhibitors.

## Conflict of interest

The authors declare no conflict of interest.

## Supporting information

As a service to our authors and readers, this journal provides supporting information supplied by the authors. Such materials are peer reviewed and may be re‐organized for online delivery, but are not copy‐edited or typeset. Technical support issues arising from supporting information (other than missing files) should be addressed to the authors.

Supplementary
