# Supplementary material for: Activity‐Directed Synthesis of Inhibitors of the p53/hDM2 Protein–Protein Interaction
Source: Chemistry. 2020 Aug 4;26(47):10682–9. doi: 10.1002/chem.202002153 (PMC7496268; doi:10.1002/chem.202002153)
Supplement: Supplementary file 1 — Supplementary [file CHEM-26-10682-s001.pdf]

Chemistry–A European Journal

Supporting Information

Activity-Directed Synthesis of Inhibitors of the p53/*h*DM2 Protein–Protein Interaction

Adam I. Green,[a, b] Fruzsina Hobor,[b, c] Christopher P. Tinworth,[d] Stuart Warriner,[a, b] Andrew J. Wilson,[a, b] and Adam Nelson*[a, b]

Contents

1. General experimental section 2

2. Synthesis of diazo compounds and reaction array components 3

3. Synthesis of *h*DM2 Ligands 6

4. Implementation of high-throughput chemistry 15

5. Fluorescence Anisotropy Assay for the p53/*h*DM2 protein-protein interaction 16

6. NMR Measurements for K_d measurement by ^{15}N HSQC NMR 29

7. LC-MS analysis of reaction mixtures 40

8. Molecular Docking and similarity calculations 44

9. Spectra 50

10. References 69

1. General Experimental

Solvents were removed under reduced pressure using a Büchi rotary evaporator with a Vacuubrand PC2001 Vario diaphragm pump, or under N_2 blowdown at 40 ^{o}C. Dry solvents and reagents were purchased from commercial suppliers and used without further purification. Rhodium catalysts were purchased from Sigma-Aldrich and used as supplied. Flash column chromatography was carried out using silica gel 60 (35-70 µm particles) supplied by Merck. Thin-layer chromatography was conducted with Macherey-Nagel Polygram SIL G/UV254 0.2mm silica gel 60 with fluorescent indicator plates.

Analytical LC-MS was performed using a system comprising an Ultimate3000 HPLC instrument with a Brucker Amazon Speed MS detector with electrospray ionisation. The system ran with a positive and negative switching mode and UV diode array detector using a Phenomenex Kinetex C18 (50 mm × 2.1 mm × 2.6 µm) column and gradient elution with two binary solvent systems: MeCN/H_2O or MeCN/H_2O plus 0.1% formic acid. Accurate mass spectrometry was performed using electrospray ionisation on a Bruker MaXis Impact spectrometer.

NMR analysis was conducted using a Bruker AV-400 spectrometer (^{1}H = 400 MHz, ^{13}C = 100 MHz and ^{19}F = 376 MHz C-F decoupled), Bruker AV-500(Cyroprobe) spectrometer (^{1}H = 500 MHz and ^{13}C = 125 MHz), JEOL ECA600ii 14.1 T spectrometer (^{1}H = 600 MHz and ^{13}C = 150 MHz), 750 MHz Oxford Magnet spectrometer (TCI-Cyroprobe, ^{1}H optimized triple resonance NMR 'inverse' probe) (^{1}H = 750 MHz and ^{15}N = 76 MHz) or a 600 MHz Oxford Magnet spectrometer (QCI-P-Cyroprobe, ^{1}H optimized quadruple resonance NMR 'inverse' probe) (^{1}H = 600 MHz and ^{15}N = 61 MHz) using an internal deuterium lock. Chemical shifts are quoted in parts per million (ppm) and coupling constants are given in Hz. Splitting patterns have been abbreviated as follows: s (singlet), d (doublet), dd (doublet of doublets), t (triplet), q (quartet) and m (multiplet). NMR data is reported in the format: ppm (number of protons, splitting pattern, coupling constant). Infrared spectra were recorded on a Bruker Alpha ATR FR-IR spectrometer; absorptions are reported in wavenumber (cm^{-1}).

The human homologue of MDM2 is referred to as *h*DM2 throughout. The p53/*h*DM2 fluorescence anisotropy assay was assembled and performed as described by Wilson *et. al.*[1] *h*DM2 protein (residues 17 to 125, with L33E mutation; referred to as $hDM2_{17\text{-}125}$) and $p53_{15\text{-}31}$-fluorescein (Ac-SQETFSDLWKLLPENNVC(Flu)-NH_2) peptide tracer, in which the fluorophore was linked to the *C*-terminal cysteine thiol through a maleimide, were used for all biological screening.

Total product concentration was used to standardise the effective screening concentrations for the high-throughput screening of reaction mixtures and is defined as the concentration of the limiting reactant in each well before the reaction took place (here, the diazo reactant).

2. Synthesis of diazo compounds

CAUTION: All diazo compounds (excluding those isolated as solid material) described below appear to be volatile at room temperature under reduced pressure. Gradual loss of mass was observed when left under high vacuum. Diazo compounds are potentially explosive on contact and should be treated with caution, although no adverse advents occurred during this study. Compounds **D1**, **D4**, **D5**, **D7**, **D8** and 2-diazo-1-(pyrrolidin-1-yl)ethenone were synthesised as previously reported.[2] Enantiomerically pure compounds (*S*)-**D4** ($[\alpha]_D^{20} = -35$) and (*R*)-**D4** ($[\alpha]_D^{20} = +28$) were prepared by the same procedure as racemic **D4**. Compound **D2** was prepared as previously reported.[3] Compound **D8** was prepared as previously reported.[4]

2.1 General procedure for the scale-up of ADS hits, A

A crimp vial (10 or 20 mL) was sequentially charged with solutions of Rhodium(II) catalyst in DCM (240 µL, 12.5 mM) and co-substrate in DCM (240 µL, 6.25 M) and stirred. A solution of diazo in DCM (240 µL, 1.25 M) was added and the vial capped. After 24 hours 900 mg of Quadrapure TU resin was added, followed by a further 720 µL DCM. After a further 24 hours the resin was removed by filtration and the solvent evaporated under reduced pressure to yield the crude reaction product.

2.2 General procedure for the scale-up of ADS hits/analogues using a syringe pump, B

A 20 mL vial was charged with Rhodium(II) catalyst (1 mol%) and degassed under N_2 atmosphere, followed by the addition of co-substrate (6.25 M) in DCM. Diazo (1.25 M) in DCM was then added dropwise to the stirred solution over 6 hours using a syringe pump. After 24 hours Quadrapure TU™ resin was then added and the reaction left for a further 24 hours. The resin was then removed by filtration and the solvent removed under reduced pressure to give a crude product.

Cyclobutylmethyl 2-(4-chlorophenyl)-2-diazoacetate, D9

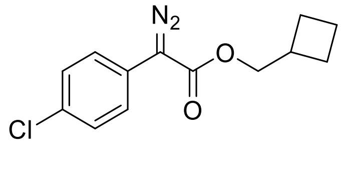

N,N-diisopropylethylamine (2.9 mL, 17 mmol) was added to a stirred suspension of EDC.HCl (882 mg, 4.6 mmol) and 4-dimethylaminopyridine (51 mg, 0.40 mmol) in DCM (50 mL), followed by the sequential addition of cyclobutane methanol (0.4 mL, 4.6 mmol) and 4-chlorophenyl acetic acid (717 mg, 4.2 mmol). After 20 hours half the solvent was removed under vacuum and the reaction mixture

washed with 10% w/v aqueous citric acid (2 x 50 mL), brine (1 x 50 mL), 10% v/v aqueous $NaHCO_3$ (2 x 50 mL), brine (1 x 50 mL) and distilled water (3 x 100 mL). The combined organic layer was then passed through a phase separation frit and concentrated under reduced pressure to give a crude oil. The oil was then dissolved in acetonitrile (42 mL, 0.1 M) and cooled to 0 ^{o}C using an ice-bath, 1,8-Diazabicyclo[5.4.0]undec-7-ene (0.9 mL, 1.4 eq) was then added followed by the portion-wise addition of 4-Acetamidobenzenesulfonyl azide (1.1 g, 1.2 eq). After 16 hours, the solvent was removed under reduced pressure to give a crude product that was dissolved in diethyl ether (40 mL) and washed sequentially with 10% w/w citric acid (2 x 10 mL), brine (2 x 10 mL), 10% w/v ammonium chloride (2 x 10 mL) and brine (2 x 10 mL). The organics were passed through a phase separation frit and concentrated under reduced pressure to give a crude material that was purified by flash column chromatography eluting 20:1 Pentane/Et_2O to give the *diazo* **D9** as an orange oil (1.1 g, 99%), R_F 0.46 (20:1 Pentane/Et_2O); δ_H (500 MHz, Acetone-d^6) 7.57 (2H, d, *J* 8.7, Ar-2H and -6H), 7.43 (2H, d, *J* 8.7, Ar-3H and -5H), 4.24 (2H, d, *J* 6.6, cyclobutylmethyl-4H_2), 2.71 (1H, dt, *J* 14.9 and 7.3, cyclobutylmethyl-3H_1), 2.12 – 2.06 (2H, m, cyclobutylmethyl-1H_2) and 1.97 – 1.81 (4H, m, cyclobutylmethyl-2H_2 and -2'H_2). δ_C (125 MHz) 165.3, 131.6, 129.7, 126.2, 126.0, 69.1, 63.7, 35.1, 25.1 and 18.9. IR ν_{max} (CH_2Cl_2 film)/cm^{-1} 2084 (diazo), 1682 and 1490. HRMS (ESI): $C_{13}H_{13}ClN_2O_2$ requires $[2M+H -N_2]^+$, calculated 501.1348, found 501.1343.

***N*-[3-(4-Chlorophenyl)-1,2-oxazol-5-yl]-2-diazoacetamide, D10**

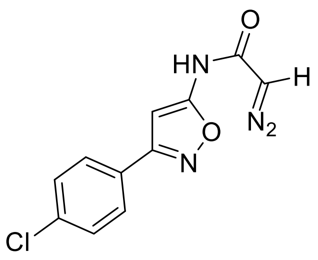

2-[(4-Methylbenzenesulfonamido)imino]acetic acid (823 mg, 3.4 mmol) was suspended in Toluene (12.5 mL) and stirred. Thionyl chloride (0.5 mL, 6.8 mmol) was added and the reaction heated to 90 ^{o}C for 3 hours, then the solvent removed under vacuum to give an orange solid. The solid was then dissolved in DCM (20 mL) and cooled to 0 ^{o}C using an ice-bath. A solution of 3-(4-chlorophenyl)isoxazole-5-amine (668 mg, 3.4 mmol) and *N*,*N*-dimethylaniline (0.5 mL, 3.7 mmol) in DCM (5 mL) was subsequently added drop-wise to the stirred solution over 5 minutes. After 1 hour triethylamine (2.3 mL, 17 mmol) was added and the reaction allowed to warm to room temperature overnight. The organics were then washed sequentially with 10% w/w citric acid (2 x 20 mL), brine (2 x 20 mL), 10% w/v ammonium chloride (2 x 20 mL) and brine (2 x 20 mL), passed through a phase

separation frit and concentrated under reduced pressure to give a crude material that was purified by flash column chromatography eluting 9:1 DCM/Et_2O to afford *diazo* **D10** as a bright orange oil (165 mg, 19%), R_F 0.09 (9:1 DCM/Et_2O); δ_H (500 MHz, Acetonitrile-d[3]) 9.14 (1H, diazoacetamide-NH), 7.82 (2H, d, *J* 8.7, phenyl-3H and -5H), 7.49 (2H, d, *J* 8.7, phenyl-2H and -6H), 6.65 (1H, s, oxazolyl-4H) and 5.31 (1H, s, diazoacetamide-2H). δ_C (125 MHz) 163.3, 163.2, 163.1, 136.5, 130.0, 129.2, 129.1, 86.3 and 49.9. IR ν_{max} (CH_2Cl_2 film)/cm^{-1} 2994, 2113 (diazo), 1678 and 1364. HRMS (ESI): $C_{11}H_7ClN_4O_2$ requires $[M+H]^+$, calculated 263.0336, found 263.0323.

2.3 Synthesis of Co-Substrates

2-Cyclopropyl-1-(1,2,3,6-tetrahydropyridin-1-yl)ethan-1-one, S5

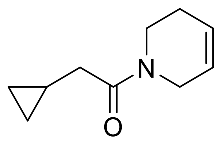

Cyclopropylacetic acid (0.50 g, 5.0 mmol) and carbonylimidazole (0.81 g, 5.0 mmol) were dissolved in THF (20 mL) and stirred for 30 minutes, followed by dropwise addition of 1,2,3,6-tetrahydropyridine (0.42 g, 5.0 mmol) in THF (4 mL). After 16 hours 1M HCl (10 mL) was added and the mixture stirred vigorously for 10 minutes. The solvent was then reduced to a minimum under vacuum and partitioned with DCM (30 mL). The organics were washed sequentially with 20% v/v $NaHCO_3$ (1 x 20 mL) and brine (1 x 20 mL), passed through a phase separation filter, and dried under vacuum to afford *amide* **S5** as a colourless oil (0.71 g, 86%). δ_H (500 MHz, Chloroform-d) 5.89 – 5.77 (1H, m, *THP-5H), 5.70 – 5.61 (1H, m, THP-4H), 4.05 – 3.90 (2H, m, THP-6H_2), 3.67 (1H, t, *J* 5.8, THP-2H_a), 3.49 (1H, t, *J* 5.8, THP-2H_b), 2.26 (2H, dd, *J* 6.8 and 12.0, ethanone-2H_2), 2.18 – 2.11 (2H, m, THP-3H_2), 1.07 – 1.01 (1H, m, cyclopropyl-1H), 0.56 – 0.51 (2H, m, cyclopropyl-2H_a and 2'H_a) and 0.18 – 0.13 (2H, m, cyclopropyl-2H_b and 2'H_b). δ_C (125 MHz) 171.5 (rot-A), 171.5 (rot-B), 126.8 (rot-A), 125.1 (rot-B), 124.6 (rot-A), 123.4 (rot-B), 45.1 (rot-A), 42.7 (rot-A), 42.0 (rot-B), 39.1 (rot-B), 38.8 (rot-A), 38.3 (rot-B), 26.0 (rot-A), 25.0 (rot-B), 7.4 (rot-A), 7.2 (rot-B), 4.6 (rot-A), 4.5 (rot-B). IR ν_{max} (CH_2Cl_2 film)/cm^{-1} 3079, 2918, 1622 and 1431. HRMS (ESI): $C_{10}H_{15}NO$ requires $[M+H]^+$, calculated 166.1231, found 166.1226. *THP = tetrahydropyridin-1-yl.

3. Synthesis of hDM2 Ligands

2-[(5-Chloro-2,3-dihydro-1H-inden-1-yl)oxy]-1-(3-phenylpyrrolidin-1-yl)ethenone, P2a and P2b

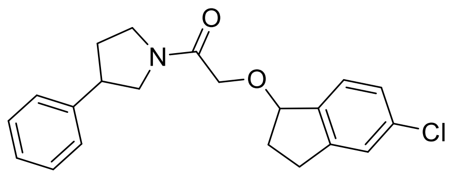

According to general procedure **A**, Rh_2piv_4 (2.8 mg, 4.6 µmol), 2-diazo-1-(3-phenylpyrrolidin-1-yl)ethan-1-one (100 mg, 0.46 mmol) and 5-chloro-2,3-dihydro-1H-inden-1-ol (391 mg, 2.32 mmol) gave an orange oil. The crude oil was then purified by flash column chromatography eluting 100% Et_2O to afford *ether* **P2** as a colourless oil (127 mg, 78%), R_F 0.18 (100% Et_2O); δ_H (600 MHz, d^6-DMSO, 1:1 mixture of diastereomers; 1:1 mixture of rotamers): 7.48 – 7.45 (1H, m, Ar-4H), 7.35 – 7.23 (7H, m, Ar), 4.97 – 4.93 (1H, m, inden-1-yloxy-1H), 4.21 – 4.14 (2H, m, ethenone-2H), 3.89 – 3.83 (1H, m, pyrrolidinyl-3H), 3.66 – 3.59 (1H, m, pyrrolidinyl-2H_a), 3.50 – 3.39 (1H, m, pyrrolidinyl-2H_b), 3.37 – 3.21 (2H, m, pyrrolidinyl-5H), 3.00 – 2.74 (2H, m, pyrrolidinyl-4H), 2.33 – 2.219 (2H, m, 2,3-dihydroindenyl-3H) and 2.03 – 1.87 (2H, m, 2,3-dihydroindenyl-2H). δ_C (150 MHz, d^6-DMSO): 167.3 (broad s, major), 167.3 (rot-A, minor), 167.2 (rot-B, minor), 146.3 (broad s, major), 146.3 (broad s, minor), 141.6 (broad s, major), 141.6 (broad s, minor), 141.2 (broad s, major), 141.1 (broad s, minor), 132.9 (broad s, major+minor), 128.5 (broad s, major+minor), 127.1 (major), 127.0 (minor), 126.8 (major), 126.8 (minor), 126.7 (broad s, major), 126.6 (broad s, minor), 126.2 (broad s, major), 126.2 (broad s, minor), 124.7 (broad s, major+minor), 82.0 (broad s, major), 81.9 (broad s, minor), 67.7 (rot-A, major), 67.7 (rot-B, major), 67.6 (broad s, minor), 51.6 (broad s, major), 51.2 (broad s, minor), 45.4 (broad s, major), 45.0 (broad s, minor), 43.7 (broad s, major), 41.6 (broad s, minor), 33.0 (broad s, major), 33.0 (broad s, minor), 32.0 (broad s, major), 32.0 (broad s, minor), 30.8 (broad s, major) and 30.0 (broad s, minor). HRMS (ESI): $C_{21}H_{22}ClNO_2$ requires $[M+H]^+$, calculated 356.1417, found 356.1425. The diastereomeric ratio was determined by analysis of the ^{13}C chemical shift for the inden-1-yloxy-C1 carbon.

2-{[(*1R*)-5-Chloro-2,3-dihydro-1H-inden-1-yl]oxy}-1-[(3S)-3-phenylpyrrolidin-1-yl]ethenone, P2a

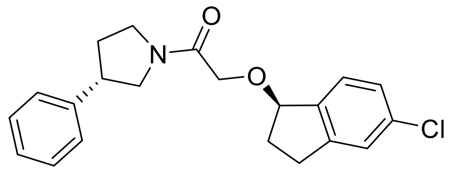

According to general procedure **A**, Rh_2piv_4 (0.6 mg, 1.0 µmol), 2-diazo-1-((*S*)-3-phenylpyrrolidin-1-yl)ethan-1-one (21.5 mg, 0.1 mmol) and (*1R*)-5-chloro-2,3-dihydro-1H-inden-1-ol (84.0 mg, 0.5 mmol)

gave an orange oil. The crude oil was then purified by flash column chromatography eluting 100% Et_2O to afford *ether* **P2a** as a colourless oil (24 mg, 67%), R_F 0.48 (100% Et_2O); δ_H (500 MHz, Chloroform-d): 7.32 – 7.09 (16H, m, Ar, rot-A and rot-B), 4.93 (1H, ddd, *J* 17.6, 6.4 and 3.7, inden-1-yloxy-1H, rot-A and rot-B), 4.11 – 4.09 (2H, m, ethenone-2H_2, rot-A and rot-B), 3.98 – 3.93 (1H, m, pyrrolidinyl-2H_a, rot-A), 3.80 – 3.72 (2H, m, pyrrolidinyl-3H, rot-A and rot-B), 3.65 – 3.60 (1H, m, pyrrolidinyl-2H_a, rot-B), 3.50 – 3.25 (6H, m, pyrrolidinyl-2H_a and -5H_2, rot-A and rot-B), 3.01 – 2.96 (2H, m, 2,3-dihydroindenyl-3H_a, rot-A and rot-B), 2.74 – 2.71 (2H, m, 2,3-dihydroindenyl-3H_b, rot-A and rot-B), 2.33 – 2.20 (4H, m, pyrrolidinyl-4H_2, rot-A and rot-B), 2.13 – 2.05 (2H, m, 2,3-dihydroindenyl-2H_a, rot-A and rot-B) and 2.00 – 1.88 (2H, m, 2,3-dihydroindenyl-2H_b, rot-A and rot B). δ_C (125 MHz, Chloroform-d): 168.3 (rot A), 168.2 (rot B), 146.4 (rot A), 146.4 (rot B), 141.0 (rot-A and rot-B), 140.8 (rot A), 140.6 (rot B), 134.6 (rot-A and rot-B), 128.9 (rot A), 128.8 (rot B), 127.2 (rot A), 127.1 (rot B), 126.8 (rot A), 126.7 (rot B), 126.6 (rot A), 126.6 (rot B), 125.3 (rot-A and rot-B), 83.1 (rot-A and rot-B), 68.6 (rot A), 68.5 (rot B), 52.6 (rot A), 52.1 (rot B), 46.1 (rot A), 46.0 (rot B), 44.7 (rot-A and rot-B), 42.3 (rot-A and rot-B), 33.8 (rot-A and rot-B), 32.6 (rot-A and rot-B), 31.3 (rot-A and rot-B) and 30.3 (rot-A and rot-B). HRMS (ESI): $C_{21}H_{22}ClNO_2$ requires $[M+Na]^+$, calculated 378.1237, found 378.1237.

2-{[(*1S*)-5-Chloro-2,3-dihydro-1H-inden-1-yl]oxy}-1-[(3R)-3-phenylpyrrolidin-1-yl]ethenone, *ent*-P2a

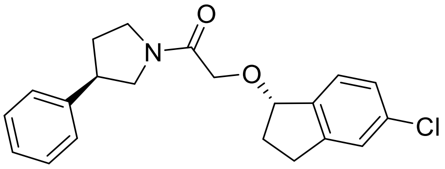

According to general procedure **A**, Rh_2piv_4 (0.6 mg, 1.0 µmol), 2-diazo-1-((*R*)-3-phenylpyrrolidin-1-yl)ethan-1-one (21.5 mg, 0.1 mmol) and (*1S*)-5-chloro-2,3-dihydro-1H-inden-1-ol (84.0 mg, 0.5 mmol) gave an orange oil. The crude oil was then purified by flash column chromatography eluting 100% Et_2O to afford *ether ent*-**P2a** as a colourless oil (19 mg, 54%), R_F 0.32 (100% Et_2O); spectroscopically identical to compound **P2a**.

2-{[(*1S*)-5-Chloro-2,3-dihydro-1H-inden-1-yl]oxy}-1-[(3S)-3-phenylpyrrolidin-1-yl]ethenone, P2b

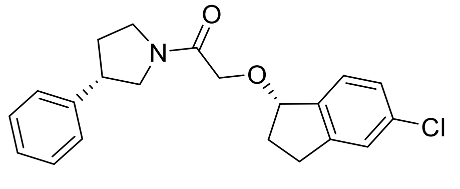

According to general procedure **A**, Rh_2piv_4 (0.6 mg, 1.0 µmol), 2-diazo-1-((*S*)-3-phenylpyrrolidin-1-yl)ethan-1-one (21.5 mg, 0.1 mmol) and (*1S*)-5-chloro-2,3-dihydro-1H-inden-1-ol (84.0 mg, 0.5 mmol) gave an orange oil. The crude oil was then purified by flash column chromatography eluting 100% Et_2O to afford *ether* **P2b** as a colourless oil (28 mg, 79%), R_F 0.47 (100% Et_2O); δ_H (500 MHz, Chloroform-d): 7.33 – 7.08 (16H, m, rot-A and rot-B), 4.94 (2H, ddd, *J* 12.4, 6.5 and 3.7, inden-1-yloxy-1H, rot-A and rot-B), 4.14 – 4.09 (4H, m, ethenone-$2H_2$, rot-A and rot-B), 3.97 – 3.93 (1H, m, pyrrolidinyl-$2H_a$, rot-A), 3.83 – 3.72 (2H, m, pyrrolidinyl-3H, rot-A and rot-B), 3.65 – 3.61 (1H, m, pyrrolidinyl-$2H_b$, rot-B), 3.48 – 3.24 (6H, m, pyrrolidinyl-$2H_a$ and -$5H_2$, rot-A and rot-B), 3.02 – 2.95 (2H, m, 2,3-dihydroindenyl-$3H_a$, rot-A and rot-B), 2.76 – 2.68 (2H, m, 2,3-dihydroindenyl-$3H_b$, rot-A and rot-B), 2.32 – 2.18 (4H, m, pyrrolidinyl-$4H_2$, rot-A and rot-B), 2.11 – 2.06 (2H, m, 2,3-dihydroindenyl-$2H_a$, rot-A and rot-B) and 2.01 – 1.84 (2H, m, 2,3-dihydroindenyl-$2H_b$, rot-A and rot B). δ_C (125 MHz, Chloroform-d): 168.3 (rot A), 168.3 (rot B), 146.4 (rot-A and rot-B), 141.0 (rot A), 140.7 (rot-A and rot-B), 140.6 (rot B), 134.6 (rot-A and rot-B), 128.9 (rot A), 128.8 (rot B), 127.2 (rot-A and rot-B), 127.1 (rot A), 127.1 (rot B), 126.8 (rot A), 126.7 (rot B), 126.6 (rot A), 126.6 (rot B), 125.3 (rot A), 125.3 (rot B), 83.2 (rot A), 83.1 (rot B), 68.7 (rot A), 68.5 (rot B), 52.6 (rot A), 52.1 (rot B), 46.1 (rot A), 46.0 (rot B), 44.7 (rot-A and rot-B), 42.3 (rot-A and rot-B), 33.7 (rot A), 32.6 (rot A and rot-B), 31.3 (rot B), 30.3 (rot-A) and 30.3 (rot B). HRMS (ESI): $C_{21}H_{22}ClNO_2$ requires $[M+Na]^+$, calculated 378.1237, found 378.1230.

2-{[(*1R*)-5-Chloro-2,3-dihydro-1H-inden-1-yl]oxy}-1-[(3R)-3-phenylpyrrolidin-1-yl]ethenone, *ent*-P2b

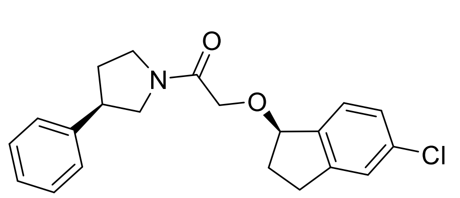

According to general procedure **A**, Rh_2piv_4 (0.6 mg, 1.0 µmol), 2-diazo-1-((*R*)-3-phenylpyrrolidin-1-yl)ethan-1-one (21.5 mg, 0.1 mmol) and (*1R*)-5-chloro-2,3-dihydro-1H-inden-1-ol (84.0 mg, 0.5 mmol) gave an orange oil. The crude oil was then purified by flash column chromatography eluting 100% Et_2O

to afford *ether ent*-**P2b** as a colourless oil (18 mg, 51%), R_F 0.59 (100% Et_2O); spectroscopically identical to compound **P2b**.

2-(Cyclopentyloxy)-1-(3-phenylpyrrolidin-1-yl)ethenone, SI1

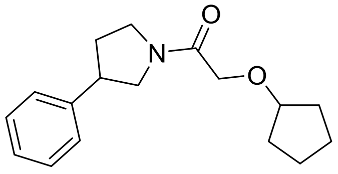

According to general procedure **B**, Rh_2piv_4 (1.4 mg, 2.5 µmol), 2-diazo-1-(3-phenylpyrrolidin-1-yl)ethan-1-one (50 mg, 0.25 mmol) and cyclopentanol (105 µL, 1.2 mmol) gave an orange oil. The crude oil was then purified by flash column chromatography eluting 100% Et_2O to afford *ether* **10** as a colourless oil (34 mg, 50%), R_F 0.29 (100% Et_2O); δ_H (500 MHz, $CDCl_3$): 7.29 – 7.16 (10H, m, Ar, rot-A and rot-B), 4.00 (4H, d, *J* 11.4, ethenone-2H_2, rot-A and rot-B), 3.98 – 3.86 (4H, m, cyclopentyloxy-1H and pyrrolidinyl -2H_a, rot-A and rot-B), 3.74 (2H, ddd, *J* 10.9, 8.2 and 2.9, pyrrolidinyl-2H_b, rot-A), 3.68 (1H, ddd, *J* 10.9, 8.2 and 2.9, pyrrolidinyl-2H_b, rot-B), 3.54 – 3.25 (6H, m, pyrrolidinyl-3H and -5H_2, rot-A and rot-B), 2.33 – 219 (2H, m, pyrrolidinyl-4H_a, rot-A and rot-B), 2.04 – 1.87 (2H, m, pyrrolidinyl-4H_b, rot-A and rot-B) and 1.67 – 1.62 (16H, m, cyclopentyloxy-2H and -3H, rot-A and rot-B). δ_C (125 MHz, $CDCl_3$): 168.6 (rot A), 168.5 (rot B), 141.1 (rot A), 140.9 (rot B), 128.9 (rot A), 128.8 (rot B), 127.2 (rot A), 127.1 (rot B), 127.1 (rot A), 127.0 (rot B), 82.4 (rot A), 82.3 (rot B), 69.4 (rot A), 69.2 (rot B), 52.6 (rot A), 52.0 (rot B), 46.1 (rot A), 46.0 (rot B), 44.8 (rot A), 42.3 (rot B), 33.8 (rot A), 32.2 (rot A), 32.2 (rot B), 31.4 (rot B), 23.6 (rot A) and 23.6 (rot B). HRMS (ESI): $C_{17}H_{23}NO_2$ requires $[M+H]^+$, calculated 274.1807, found 274.1803.

2-[(5-Chloro-2,3-dihydro-1H-inden-1-yl)oxy]-1-(pyrrolidin-1-yl)ethenone, SI2

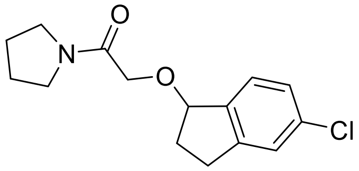

According to general procedure **B**, Rh_2piv_4 (2.2 mg, 3.6 µmol), 2-diazo-1-(pyrrolidin-1-yl)ethenone (50 mg, 0.36 mmol) and 5-chloro-2,3-dihydro-1H-inden-1-ol (120 mg, 0.72 mmol) gave an orange oil. The crude oil was then purified by flash column chromatography eluting 100% Et_2O to afford *ether* **11** as a colourless oil (51 mg, 50%), R_F 0.09 (100% Et_2O); δ_H (500 MHz, $CDCl_3$): 7.37 (1H, d, *J* 8.0, indenyloxy-7H), 7.22 (1H, broad s, indenyloxy-4H), 7.18 – 7.16 (1H, m, indenyloxy-6H), 4.99 (1H, dd, *J* 6.5 and 3.7, indenyloxy-1H), 4.14 (2H, m, ethenone-1H), 3.49 (2H, app. t, *J* 6.9, pyrrolidinyl-2H_a), 3.45 – 3.38 (2H,

m, pyrrolidinyl-2H_b), 3.09 – 3.03 (1H, m, indenyloxy-3H_a), 2.81 – 2.76 (1H, m, indenyloxy-3H_b), 2.35 (1H, ddt, *J* 13.0, 8.5 and 6.4, indenyloxy-2H_a), 2.15 (1H, dddd, *J* 13.3, 8.4, 4.8 and 3.8, indenyloxy-2H_b), 1.95 – 1.90 (2H, m, pyrrolidinyl-3H_a) and 1.86 – 1.81 (2H, m, pyrrolidinyl-3H_b). δ_C (125 MHz, $CDCl_3$) 168.3, 146.4, 140.7, 134.5, 126.7, 126.6, 125.2, 83.0, 68.5, 46.2 (rot-A), 46.1 (rot-B), 32.5, 30.3, 26.3 (rot-A) and 24.0 (rot-B). HRMS (ESI): $C_{15}H_{18}ClNO_2$ requires $[M+Na]^+$, calculated 302.0924, found 302.0926.

2,2,2-Trifluoroethyl 2-(6-chloro-1H-indol-3-yl)-2-(4-chlorophenyl)acetate, P1

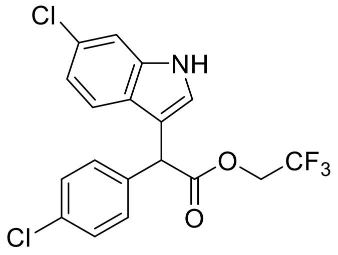

According to general procedure **B**, Rh_2pfb_4 (3.8 mg, 3.6 µmol), 2,2,2-trifluoroethyl 2-(4-chlorophenyl)-2-diazoacetate (100 mg, 0.36 mmol) and 6-chloroindole (272 mg, 1.8 mmol) gave an orange oil. The crude oil was then purified by flash column chromatography eluting 9:1 pentane/Et_2O to afford *indole* **P1** as a colourless oil (20 mg, 14%), R_F 0.19 (3:1 Pentane/Et_2O); δ_H (500 MHz, $CDCl_3$): 8.10 (1H, s, 6-chloroindolyl-NH), 7.30 (1H, d, *J* 1.8, 6-chloroindolyl-4H), 7.28 – 7.24 (4H, m, 4-chlorophenyl-2H_2 and -3H_2), 7.21 (1H, d, *J* 8.5, 6-chloroindolyl-2H), 7.14 (1H, dd, *J* 2.5 and 0.8, 6-chloroindolyl-7H), 6.99 (1H, dd, *J* 8.5 and 1.8, 6-chloroindolyl-5H), 5.24 (1H, s, acetate-2H) and 4.49 (2H, qq, *J* 12.7 and 8.4, trifluoroethyl-1H). δ_C (125 MHz, $CDCl_3$): 170.9, 136.8, 135.8, 133.9, 129.8, 129.1, 128.8, 124.9, 124.0, 121.8, 121.0, 119.9, 112.5, 111.5, 61.0 (q, $J_{C\text{-}F}$ 36.7), 47.9. HRMS (ESI): $C_{18}H_{12}Cl_2F_3NO_2$ requires $[M+H]^+$, calculated 402.0275, found 402.0270.

6,6'-Dichloro-1H,1'H,3H-[3,3'-biindol]-2-one, P6

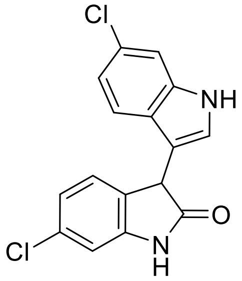

According to general procedure **A**, Rh_2pfb_4 (2.8 mg, 2.6 µmol), 6-chloro-3-diazo-1H-indol-2-one (50 mg, 0.26 mmol) and 6-chloroindole (254 mg, 1.3 mmol) gave an orange oil. The crude oil was then purified by flash column chromatography eluting 9:1 DCM/Et_2O to afford *oxindole* **P6** as a colourless oil (44 mg, 53%), R_F 0.17 (9:1 DCM/Et_2O); δ_H (500 MHz, $CDCl_3$): 8.54 (1H, broad s, biindol-2-one-NH), 8.29 (1H, broad s, biindol-NH), 7.31 (1H, d, *J* 1.8 Hz, biindol-4H), 7.14 (1H, d, *J* 8.4, biindol-2H), 7.06 – 7.04 (2H, m, biindol-2-one-4H and -7H), 6.99 (2H, td, *J* 8.4 and 1.8, biindol-2-one-5H and biindol-5H), 6.94 (1H, d, *J* 1.8, biindol-7H) and 4.83 (1H, s, biindol-2-one-3H). δ_C (125 MHz, $CDCl_3$): 178.5, 142.4, 137.1, 134.2, 128.7, 127.8, 126.1, 124.7, 124.3, 122.9, 121.0, 120.1, 111.5, 110.7, 110.4 and 44.5. HRMS (ESI): $C_{16}H_{10}Cl_2N_2O$ requires $[M+H]^+$, calculated 317.0248, found 317.0226.

5',6-Dichloro-1H,1'H,3H-[3,3'-biindol]-2-one, SI3

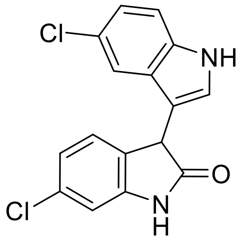

According to general procedure **B**, Rh_2pfb_4 (2.8 mg, 2.6 µmol), 6-chloro-3-diazo-1H-indol-2-one (50 mg, 0.26 mmol) and 5-chloroindole (254 mg, 1.3 mmol) gave an orange oil. The crude oil was then purified by flash column chromatography eluting 9:1 DCM/Et_2O to afford *oxindole* **12** as a colourless oil (31.2 mg, 38%), R_F 0.17 (9:1 DCM/Et_2O); δ_H (500 MHz, DMSO-d_6): 11.27 (1H, s, biindol-2-one-NH), 10.71 (1H, s, biindol-NH), 7.39 (1H, dd, *J* 8.6 and 0.5, biindol-2-one-4H), 7.31 (1H, d, *J* 2.5, biindol-4H), 7.11 – 7.04 (3H, m, biindol-2-one-5H, biindol-2H and -5H), 6.98 – 6.95 (2H, m, biinol-2-one-7H and biindol-7H) and 4.98 (1H, s, biindol-2-one-3H). δ_C (125 MHz, DMSO-d_6): 177.4, 144.0, 134.9, 132.1, 128.9, 127.1, 126.2, 125.9, 123.3, 121.3, 121.2, 117.6, 113.3, 109.4, 109.4 and 43.6. HRMS (ESI): $C_{16}H_{10}Cl_2N_2O$ requires $[M+H]^+$, calculated 317.0248, found 317.0237.

(1S*,3R*)-6'-Chloro-3-(4-chlorophenyl)-1'H-spiro[cyclopropane-1,3'-indol]-2'-one, P5

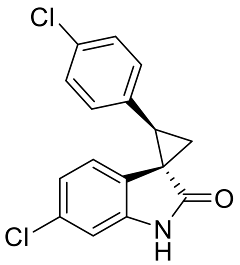

According to general procedure **A**, Rh_2piv_4 (1.6 mg, 2.6 µmol), 6-chloro-3-diazo-1H-indol-2-one (50 mg, 0.26 mmol) and 4-chlorostyrene (156 µL, 1.3 mmol) gave an orange oil. The crude oil was then purified by flash column chromatography eluting 9:1 DCM/Et_2O to afford *oxindole* **P5** as a colourless oil (46 mg, 58%), R_F 0.17 (9:1 DCM/Et_2O); δ_H (500 MHz, $CDCl_3$): 8.57 (1H, broad s, indolone-NH), 7.28 (2H, d, *J* 8.2, 4-chlorophenyl-3H_2), 7.11 (2H, d, *J* 8.2, 4-chlorophenyl-2H_2), 6.96 (1H, d, *J* 1.8, indol-2-one-7H), 6.69 (1H, dd, *J* 8.1 and 1.9, indol-2-one-5H) 5.84 (1H, d, *J* 8.1, indol-2-one-4H), 3.28 (1H, app. t, *J* 8.6, cyclopropane-1H), 2.23 (1H, dd, *J* 9.2 and 4.8, cyclopropane-2H_a), 1.97 (1H, dd, *J* 8.0 and 4.8, cyclopropane-2H_b). δ_C (125 MHz, $CDCl_3$): 178.3, 142.0, 133.7, 133.3, 132.8, 131.4, 128.9, 126.0, 121.9, 121.8, 110.5, 35.7, 33.5 and 22.9. HRMS (ESI): $C_{16}H_{11}Cl_2NO$ requires $[M+H]^+$, calculated 304.0296, found 304.0286.

(1S*,3R*)-6'-Chloro-3-(3-chlorophenyl)-1'H-spiro[cyclopropane-1,3'-indol]-2'-one, SI4

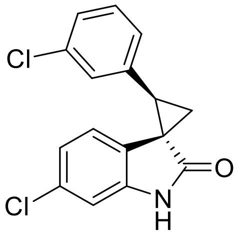

According to general procedure **B**, Rh_2piv_4 (1.6 mg, 2.6 µmol), 6-chloro-3-diazo-1H-indol-2-one (50 mg, 0.26 mmol) and 3-chlorostyrene (165 µL, 1.3 mmol) gave an orange oil. The crude oil was then purified by flash column chromatography eluting 9:1 DCM/Et_2O to afford *oxindole* **13** as a colourless oil (39 mg, 49%), R_F 0.38 (9:1 DCM/Et_2O); δ_H (500 MHz, $CDCl_3$): 8.42 (1H, br s, indol-2-one-NH), 7.27 – 7.22 (3H, m, 3-chlorophenyl-4H, -5H and -6H), 7.04 (1H, broad dd, *J* 7.3 and 0.6, 3-chlorophenyl-2H), 6.96 (1H, d, *J* 1.8, indol-2-one-7H), 6.69 (1H, dd, *J* 8.1 and 1.9, indol-2-one-5H), 5.87 (1H, d, *J* 8.1, indol-2-one-4H), 3.29 (1H, app. t, *J* 8.6, cyclopropane-1H), 2.23 (1H, dd, *J* 9.2 and 4.8, cyclopropane-2H_a) and 1.99 (1H, dd, *J* 8.0 and 4.8, cyclopropane-2H_b). δ_C (125 MHz, $CDCl_3$): 178.0, 142.0, 136.9, 134.6, 132.9,

130.0, 129.9, 129.9, 128.3, 128.1, 125.9, 121.9, 110.5, 35.8, 33.5 and 22.7. HRMS (ESI): $C_{16}H_{11}Cl_2NO$ requires $[M+H]^+$, calculated 304.0296, found 304.0285.

6-Chloro-3-(4-chlorophenyl)-1-methyl-3H-indol-2-one, P3

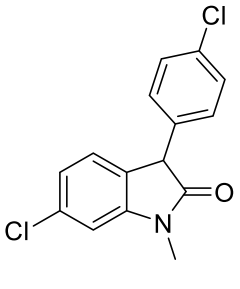

According to general procedure **A**, Rh_2piv_4 (1.9 mg, 3.0 µmol) and N,2-bis(4-chlorophenyl)-2-diazo-N-methylacetamide (100 mg, 0.3 mmol) gave an orange oil. The crude oil was then purified by flash column chromatography initially eluting 100% DCM, which gave a mixture of products, and the oil was re-purified eluting 8:2 pentane/EtOAc to afford *oxindole* **P3** as a colourless oil (3.5 mg, 4%), R_F 0.15 (8:2 pentane/EtOAc); δ_H (500 MHz, $CDCl_3$): 7.33 – 7.31 (3H, m, 4-chlorophenyl-3H_2 and indol-2-one-4H), 7.14 – 7.11 (3H, m, 4-chlorophenyl-2H_2 and indol-2-one-5H), 6.82 (1H, d, *J* 8.3, indol-2-one-4H), 4.57 (1H, s, indol-2-one-3H) and 3.24 (3H, s, indol-2-one-NCH_3). δ_C (125 MHz, $CDCl_3$): 175.2, 143.2, 134.4, 134.0, 130.0, 129.9, 129.3, 128.8, 128.4, 125.6, 109.4, 51.5 and 26.8. HRMS (ESI): $C_{15}H_{11}Cl_2NO$ requires $[M+H]^+$, calculated 292.0295, found 292.0279.

***N*,2-Bis(4-chlorophenyl)-*N*-methyl-2-oxoacetamide, P4**

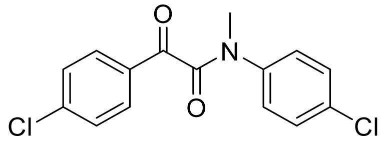

According to general procedure **A**, Rh_2piv_4 (1.9 mg, 3.0 µmol) and N,2-bis(4-chlorophenyl)-2-diazo-N-methylacetamide (100 mg, 0.3 mmol) gave an orange oil. The crude oil was then purified by flash column chromatography initially eluting 100% DCM, which gave a mixture of products, and the oil was re-purified eluting 8:2 pentane/EtOAc to afford *oxoacetamide* **P4** as a colourless oil (3.6 mg, 4%), R_F 0.38 (8:2 pentane/EtOAc); δ_H (500 MHz, $CDCl_3$): 7.81 (2H, d, *J* 8.4, acetamide Ar-3H), 7.44 (2H, d, *J* 8.4, acetamide Ar-2H), 7.24 (2H, d, *J* 8.5, oxo Ar-3H), 7.06 (2H, d, *J* 8.5, oxo Ar-2H) and 3.45 (3H, s, oxoacetamide-NCH_3). δ_C (125 MHz, $CDCl_3$): 189.3, 166.6, 141.3, 139.8, 134.3, 131.9, 130.9, 130.0,

129.5, 128.2 and 36.5. HRMS (ESI): $C_{15}H_{11}Cl_2NO_2$ requires $[M+Na]^+$, calculated 330.0064, found 330.0059.

3-(Ethoxymethyl)-1'H-spiro[cyclopropane-1,3'-indol]-2'-one, P7

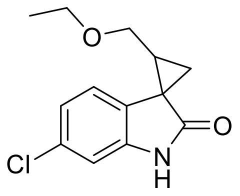

According to general procedure **A**, Rh_2piv_4 (1.3 mg, 2.1 µmol), 6-chloro-3-diazo-1H-indol-2-one (40 mg, 0.21 mmol) and allyl methyl ether (113 µL, 1.0 mmol) gave an orange oil. The crude oil was then purified by flash column chromatography eluting 9:1 DCM/Et_2O to afford *oxindole* **P7** as a colourless oil (7.3 mg, 14%), R_F 0.08 (9:1 DCM/Et_2O); δ_H (500 MHz, $CDCl_3$): 8.31 (1H, s, indol-2-one-NH), 6.98 (1H, dd, J 8.0 and 1.9, indol-2-one-5H), 6.94 (1H, d, J 1.5, indol-2-one-7H), 6.90 (1H, d, J 8.0, indol-2-one-4H), 3.77 (1H, dd, J 11.2 and 5.5, methyl-H_a), 3.64 (1H, dd, J 11.2 and 7.6, methyl-H_b), 3.45 (2H, q, J 7.0, ethoxy-1H_2), 2.24 (1H, dtd, J 13.1, 7.6 and 5.5, cyclopropane-3H), 1.95 (1H, dd, J 9.4 and 4.5, cyclopropane-2H_a), 1.57 (1H, dd, J 7.8 and 4.5, cyclopropane-2H_b) and 1.16 (3H, t, J 7.0, ethoxy-2H_3). δ_C (125 MHz, $CDCl_3$): 178.3, 142.3, 132.7, 126.9, 122.2, 121.8, 110.6, 67.4, 66.3, 32.0, 31.6, 22.0 and 15.2. HRMS (ESI): $C_{13}H_{14}ClNO_2$ requires $[M+H]^+$, calculated 252.0791, found 252.0777.

4. Implementation of high-throughput chemistry for Activity-Directed Synthesis Reaction Arrays

Activity-Directed Synthesis reactions were carried out in 0.75 mL shell vials (Chemglass CV-2100-0830) equipped with a teflon-coated stir bar (Biotage 0.2-0.5 mL magnetic stir bar #355545) and sealed using either a Freeslate 96-well reaction block or a Sigma-Aldrich Kitalysis 24-well reaction block (Z742107 Aldrich). Prior to the assembly of each reaction array the following stock solutions were made: diazo reaction solvent (1.25 M); catalyst in THF (25 mM); and co-substrate in DCM (6.25 M). Each reaction vial was charged with catalyst stock (8 µL) and the solvent allowed to evaporate to dryness, then DCM (84 µL) was added and the reaction block placed on a magnetic stirring plate. Each reaction vial was then sequentially charged with co-substrate stock (8 µL) and diazo stock (8 µL), then the plate sealed using a Teflon film and stirred. After 24 hours Quadrapure TU resin (30 mg) was added to each vial and left overnight to scavenge the catalyst. The solvent was then evaporated under a stream of nitrogen gas and the crude material dissolved in molecular biology grade DMSO (200 µL) to create a biological screening master stock (50 mM total product concentration) that was passed through a 96-well filter plate (Agilent Technologies: #200933-100) and stored at –20 °C.

Example 96-well reaction plate layout:

		1	2	3	4	5	6	7	8	9	10	11	12	Catalyst
D1	A	**S1**	**S2**	**S3**	**S4**	**S5**	**S6**	**S7**	**S8**	**S9**	**S10**	D1 Control	Rh_2piv_4 Control	**PIV**
D2	B	**S1**	**S2**	**S3**	**S4**	**S5**	**S6**	**S7**	**S8**	**S9**	**S10**	D2 Control	BLANK	**PIV**
D3	C	**S1**	**S2**	**S3**	**S4**	**S5**	**S6**	**S7**	**S8**	**S9**	**S10**	D3 Control	BLANK	**PIV**
D4	D	**S1**	**S2**	**S3**	**S4**	**S5**	**S6**	**S7**	**S8**	**S9**	**S10**	D4 Control	BLANK	**PIV**
D1	E	**S1**	**S2**	**S3**	**S4**	**S5**	**S6**	**S7**	**S8**	**S9**	**S10**	D1 Control	Rh_2pfb_4 Control	**PFB**
D2	F	**S1**	**S2**	**S3**	**S4**	**S5**	**S6**	**S7**	**S8**	**S9**	**S10**	D2 Control	BLANK	**PFB**
D3	G	**S1**	**S2**	**S3**	**S4**	**S5**	**S6**	**S7**	**S8**	**S9**	**S10**	D3 Control	BLANK	**PFB**
D4	H	**S1**	**S2**	**S3**	**S4**	**S5**	**S6**	**S7**	**S8**	**S9**	**S10**	D4 Control	BLANK	**PFB**

Example 384-well biological assay plate layout:

	1	2	3	4	5	6	7	8	9	10	11	12	13	14	15	16	17	18	19	20	21	22	23	24
A	A1	A1	A2	A2	A3	A3	A4	A4	A5	A5	A6	A6	A7	A7	A8	A8	A9	A9	A10	A10	A11	A11	PIV	PIV
B	A1 B	A1 B	A2 B	A2 B	A3 B	A3 B	A4 B	A4 B	A5 B	A5 B	A6 B	A6 B	A7 B	A7 B	A8 B	A8 B	A9 B	A9 B	A10 B	A10 B	A11 B	A11 B	PIV B	PIV B
C	B1	B1	B2	B2	B3	B3	B4	B4	B5	B5	B6	B6	B7	B7	B8	B8	B9	B9	B10	B10	B11	B11	neg	neg
D	B1 B	B1 B	B2 B	B2 B	B3 B	B3 B	B4 B	B4 B	B5 B	B5 B	B6 B	B6 B	B7 B	B7 B	B8 B	B8 B	B9 B	B9 B	B10 B	B10 B	B11 B	B11 B	B	B
E	C1	C1	C2	C2	C3	C3	C4	C4	C5	C5	C6	C6	C7	C7	C8	C8	C9	C9	C10	C10	C11	C11	neg	neg
F	C1 B	C1 B	C2 B	C2 B	C3 B	C3 B	C4 B	C4 B	C5 B	C5 B	C6 B	C6 B	C7 B	C7 B	C8 B	C8 B	C9 B	C9 B	C10 B	C10 B	C11 B	C11 B	B	B
G	D1	D1	D2	D2	D3	D3	D4	D4	D5	D5	D6	D6	D7	D7	D8	D8	D9	D9	D10	D10	D11	D11	neg	neg
H	D1 B	D1 B	D2 B	D2 B	D3 B	D3 B	D4 B	D4 B	D5 B	D5 B	D6 B	D6 B	D7 B	D7 B	D8 B	D8 B	D9 B	D9 B	D10 B	D10 B	D11 B	D11 B	B	B
I	E1	E1	E2	E2	E3	E3	E4	E4	E5	E5	E6	E6	E7	E7	E8	E8	E9	E9	E10	E10	E11	E11	PFB	PFB
J	E1 B	E1 B	E2 B	E2 B	E3 B	E3 B	E4 B	E4 B	E5 B	E5 B	E6 B	E6 B	E7 B	E7 B	E8 B	E8 B	E9 B	E9 B	E10 B	E10 B	E11 B	E11 B	PFB B	PFB B
K	F1	F1	F2	F2	F3	F3	F4	F4	F5	F5	F6	F6	F7	F7	F8	F8	F9	F9	F10	F10	F11	F11	pos	pos
L	F1 B	F1 B	F2 B	F2 B	F3 B	F3 B	F4 B	F4 B	F5 B	F5 B	F6 B	F6 B	F7 B	F7 B	F8 B	F8 B	F9 B	F9 B	F10 B	F10 B	F11 B	F11 B	pos B	pos B
M	G1	G1	G2	G2	G3	G3	G4	G4	G5	G5	G6	G6	G7	G7	G8	G8	G9	G9	G10	G10	G11	G11	pos	pos
N	G1 B	G1 B	G2 B	G2 B	G3 B	G3 B	G4 B	G4 B	G5 B	G5 B	G6 B	G6 B	G7 B	G7 B	G8 B	G8 B	G9 B	G9 B	G10 B	G10 B	G11 B	G11 B	pos B	pos B
O	H1	H1	H2	H2	H3	H3	H4	H4	H5	H5	H6	H6	H7	H7	H8	H8	H9	H9	H10	H10	H11	H11	pos	pos
P	H1 B	H1 B	H2 B	H2 B	H3 B	H3 B	H4 B	H4 B	H5 B	H5 B	H6 B	H6 B	H7 B	H7 B	H8 B	H8 B	H9 B	H9 B	H10 B	H10 B	H11 B	H11 B	pos B	pos B

5. Fluorescence Anisotropy Assay for the inhibition of the p53/*h*DM2 protein-protein interaction

5.1 *h*DM2 protein expression

The pet28a His_{10} – *h*DM2 (17-125) L33E construct[1d] was over-expressed in the *E.coli* strain Rosetta 2. An overnight starter culture (10 mL) was used to inoculate 2xYT medium (1 L) containing Kanamycin (50 µg/ml. Cultures were grown at 37 °C until the optical density of the cell suspension reached OD_{600} = 0.6 – 0.8, then the temperature was switched to 18 °C and protein expression induced by the addition of IPTG (1 mM). Induced cultures were grown at 18 °C overnight before harvesting by centrifugation for 10 minutes at 8655 xg. Cells were resuspended in lysis buffer (20 mM TRIS pH 8.0, 500 mM NaCl, 15 mM imidazole) and lysed by sonication in the presence of 10 µL of 1 U/ml^{-1} DNaseI per liter of over-expression culture, protease inhibitor cocktail tablet (Roche) and lysozyme. The cell lysate was cleared by centrifugation (Sorvall SS34 rotor, 17,000 rpm, 45 min, 8 °C) and the supernatant was filtered (0.22 µM syringe filter) before loaded onto a 5 ml HisTrap that had previously been equilibrated with lysis buffer. The HisTrap was washed with 10 column volumes (CV) of a wash buffer containing 20 mM TRIS pH 8.0, 500 mM NaCl and 15 mM imidazole, followed by 6 CV two further wash buffers containing 20 mM TRIS pH 8.0, 500 mM NaCl and 50 mM imidazole, and 6 CV 20 mM TRIS pH 8.0, 500 mM NaCl and 100 mM imidazole. The His-*h*DM2 fusion protein was then eluted from the HisTrap with an elution buffer containing 20 mM TRIS pH 8.0, 500 mM NaCl and 300 mM imidazole. The His-*h*DM2 fusion protein was dialysed overnight at 4 °C against 20 mM TRIS pH 8.0, 250 mM NaCl in the presence of TEV protease to remove the tag. To remove any uncleaved *h*DM2, the cleaved tag and the protease, the sample was reapplied to a HisTrap in 20 mM TRIS pH 8.0, 250 mM NaCl and the flow through containing the cleaved *h*DM2 was collected then concentrated (Amicon Ultra centrifugal filter, MWCO 10,000) to approximately 10 ml. The sample was then filtered before being loaded onto a Superdex 75 column (GE healthcare) equilibrated with 20 mM TRIS pH 8.0, 250 mM NaCl, 0.5 mM DTT, 2.5% Glycerol. The purified protein was concentrated and stored at –80 °C.

The quality and purity of the preparation was assessed by mass spec and circular dichroism spectroscopy. The activity of the protein was verified by testing its binding to fluorescently labelled p53 peptide in a fluorescence anisotropy assay (Figure S1). For ^{15}N labeled protein the expression was carried out using the same method but using M9 minimal media supplemented with $^{15}NH_4Cl$ as nitrogen source.

5.2 Fluorescence anisotropy assays

The fluorescein-labelled $p53_{15\text{-}31\ Flu}$ transactivation domain peptide (Ac-SQETFSDLWKLLPENNVC(Flu)-NH_2) was purchased from Peptide Synthetics. The assay was carried out using Perkin-Elmer 384-well Opti-plate assay plates (6007270). Fluorescence anisotropy assays were performed in a buffer containing 40 mM phosphate pH 7.5, 200 mM NaCl and 0.02 mg/mL bovine serum albumin (PBSA).

Results were collected using a Perkin-Elmer Envision 2103 Multilabel Reader using a 431 nm mirror, 480(104) nm excitation filter, and 535(208) and 535(209) nm emission filters after 2.5 or 24 hours of incubation at room temperature. Test well anisotropy values were then calculated using the blank corrected *S* and *P* channel values using the following formula:

Eq. 1: $$Intensity = (2\ \times P_{corrected}\ \times G\ factor) + S_{corrected}$$

Eq. 2: $$Anisotropy = \frac{S_{corrected} - G \times P_{corrected}}{Intensity}$$

The fraction of bound tracer was calculated using the following formula:

Eq. 3: $$Fraction\ Bound = \frac{(r - r_{\min})}{(\lambda(r - r_{max}) - (r - r_{min}))}$$

Where λ is the intensity of bound/unbound tracer ($\lambda = I_{bound}/I_{unbound}$) and r is anisotropy.

The fraction of ligand bound was then multiplied by the concentration of $p53_{15\text{-}31\ Flu}$ and fit to the model in equation 4 to obtain K_d.

Eq. 4: $$y = \frac{K_d + x + [FL] - \sqrt{(K_d + x + [FL])^2 - 4x[FL]}}{2}$$

Where y is the fraction bound of $p53_{15\text{-}31\ Flu}$ multiplied by 54.5 ($p53_{15\text{-}31\ Flu}$ concentration, nM), [FL] is $p53_{15\text{-}31\ Flu}$, and x is the concentration of *h*DM2. The observed K_d for the $p53_{15\text{-}31\ Flu}$:*h*DM2 binding was 180 ± 30 nM.

5.3 Binding of $p53_{15\text{-}31\ Flu}$ to *h*DM2

A serial dilution of *h*DM2 (0.0006 µM to 20.75 µM, final concentration) was added to a fixed concentration of $p53_{15\text{-}31\ Flu}$ (54.5 nM) and PBSA buffer (20 µL), to give a 60 µL total volume per assay well. Each dilution was performed in triplicate and the measured intensity of each well calculated using equation 1, then anisotropy was calculated using equation 2. The fraction bound of the tracer could also be determined using equation 3.

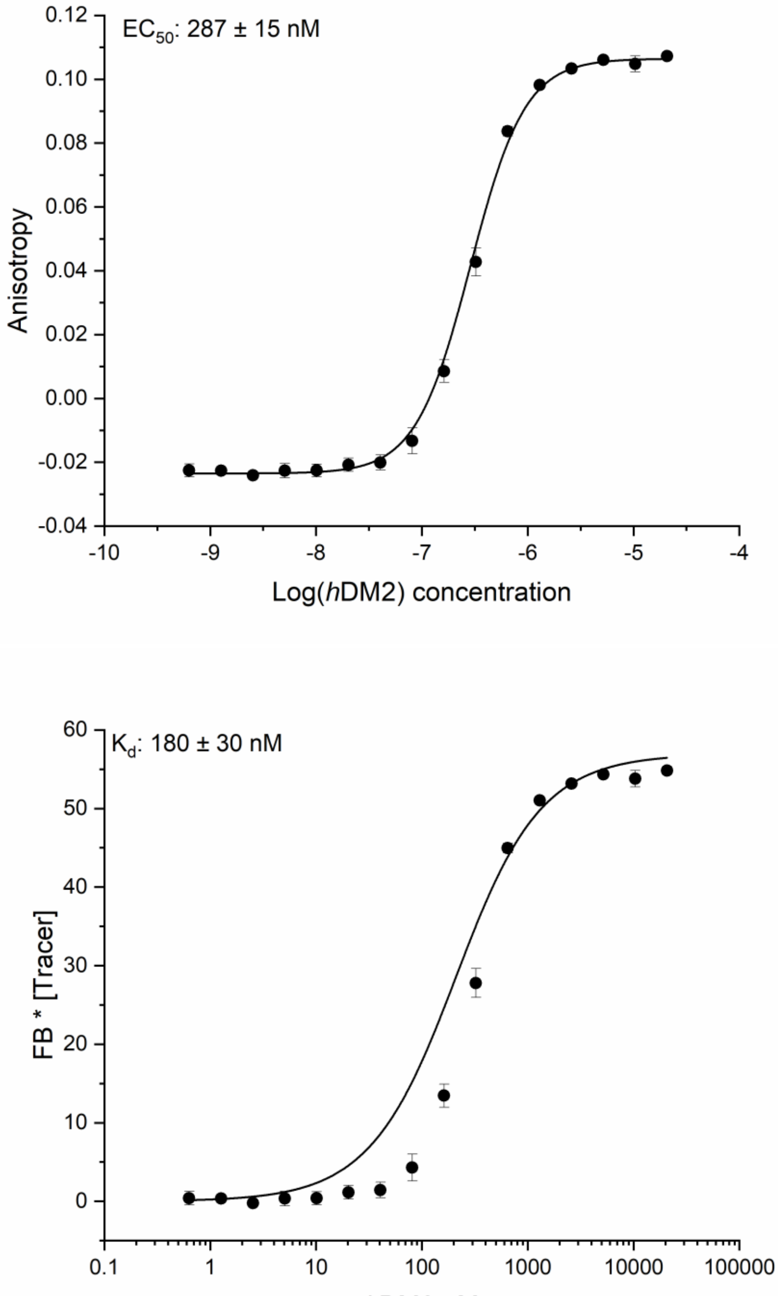

Figure S1. Fluorescence anisotropy titration of hDM2 (0.0006 µM to 20.75 µM) into fixed concentration the fluorescein-labelled p53 tracer (54.5 nM) in aqueous phosphate buffer (pH 7.5, 40 mM phosphate, 200 mM NaCl and 0.02 mg/mL Bovine Serum Albumin). FB = Fraction bound.

5.4 Inhibition of the $p53_{15\text{-}31\ Flu}$/*h*DM2 protein-protein interaction with Nutlin-3a

Nutlin-3a was serially diluted in DMSO and then diluted 33-fold in PBSA to give effective concentrations between 73 µM and 0.4 nM in 3% DMSO/PBSA. Each serial dilution was repeated in triplicate. An aliquot of each point (20 µL) was then added to a 384-well assay plate, followed by *h*DM2 (150 nM) and $p53_{15\text{-}31\ Flu}$ (25 nM), to give final concentrations of Nutlin-3a between 24 µM and 0.08 nM (Figure S2).

EC_{50} values were determined and curves were fit in Origin Pro 2019b using a non-linear curve fitting with the dose response fitting procedure (equation 5) and Levenberg Marquardt iteration algorithm.

Eq. 5: $$y = \frac{r_{min} + (r_{max} - r_{min})}{1 + 10^{((EC_{50} - x) \times p)}}$$

Where *p* is the Hill slope and EC_{50} is the concentration for half-maximal response from the baseline.

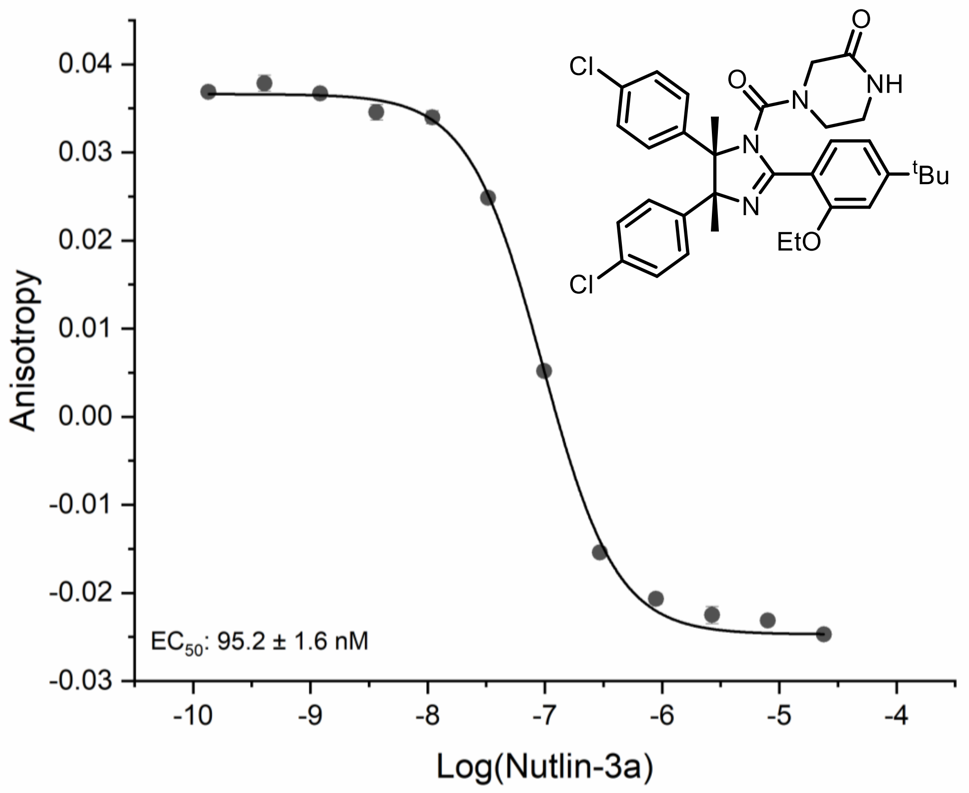

Figure S2. Dose response of Nutlin-3a (positive control) in the p53/hDM2 fluorescence anisotropy assay in pH 7.5 aqueous phosphate buffer (40 mM phosphate, 200 mM NaCl and 0.02 mg/mL Bovine Serum Albumin). Observed EC_{50}: 95.2 ± 1.6 nM, reported EC_{50}: 90 nM.[5]

5.5 Procedure for screening reaction mixtures at 20 µM:

An aliquot from the master stock of each reaction mixture (2 µL, 50 mM) was diluted into 48 µL DMSO to create a 2 mM total product concentration intermediate screening stock that was used for all subsequent reaction mixture screening. An aliquot of each 2 mM reaction mixture stock (4.9 µL) was then diluted into 155.1 µL PBSA buffer (pH 7.5, 40 mM phosphate, 200 mM NaCl and 0.02 mg/mL Bovine Serum Albumin, PBSA) to create a 60 µM 3% DMSO screening stock. 20 µL of each screening stock was then added to its corresponding well in a 384-well PerkinElmer Opti-plate (see example plate layouts in section 4). Each test well was then charged sequentially with 20 µL 450 nM *h*DM2 in pH 7.5 PBSA buffer and 20 µL 75 nM p53-tracer in pH 7.5 PBSA buffer. Each blank well was then charged with 20 µL 450 nM *h*DM2 in pH 7.5 PBSA buffer and 20 µL pH 7.5 PBSA buffer. The total volume of each well was 60 µL and the final concentrations of each reagent were:

- Reaction mixture: 20 µM (Total Product Concentration)
- *h*DM2: 150 nM
- p53-tracer: 25 nM

Percentage inhibition values were then calculated using Nutlin-3a (10 µM) as the positive control reference and a 1% DMSO blank well containing 150 nM *h*DM2 and 25 nM p53-tracer as the negative control reference.

$$\% \, Inhibition \; relative \; to \; 10 \; \mu M \; Nutlin-3a = \frac{DMSO \; Control \; Anisotropy - Sample \; Anisotropy}{Negative \; Control \; Anisotropy - Positive \; Control \; Anisotropy} \times 100$$

Round 1 HTS at 20 µM total product concentration:

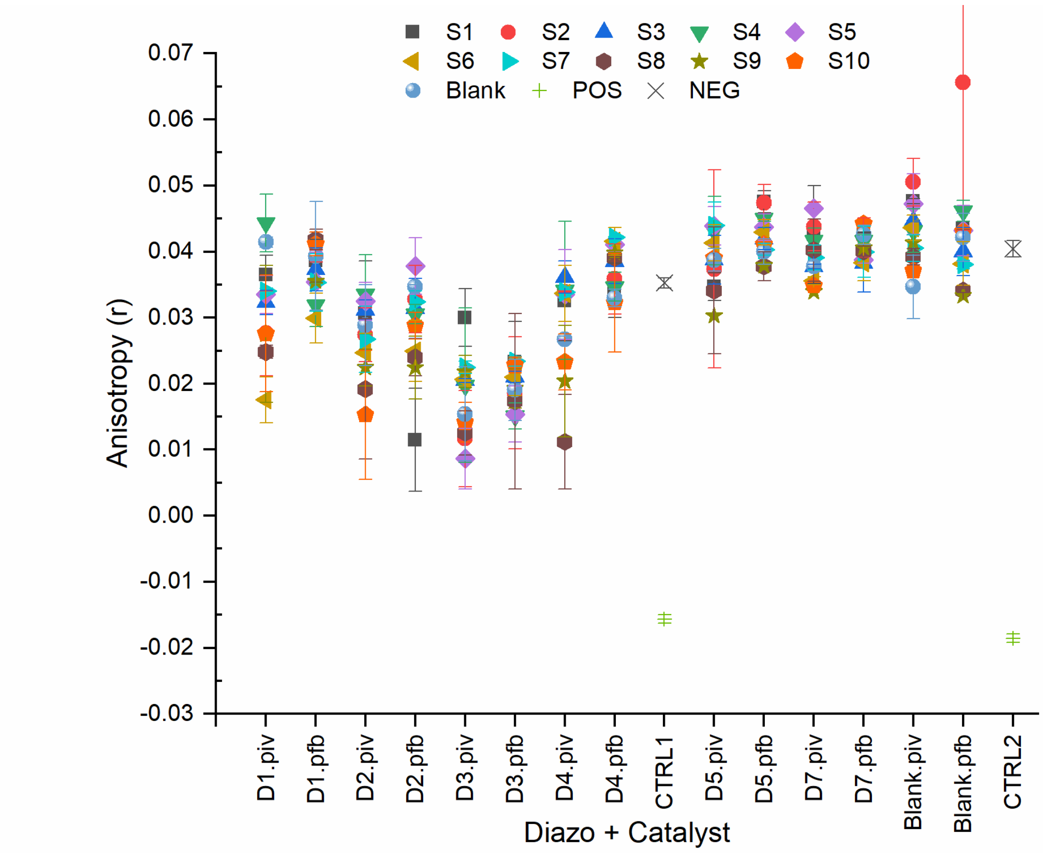

Reaction array 1 controls for individual reagents:

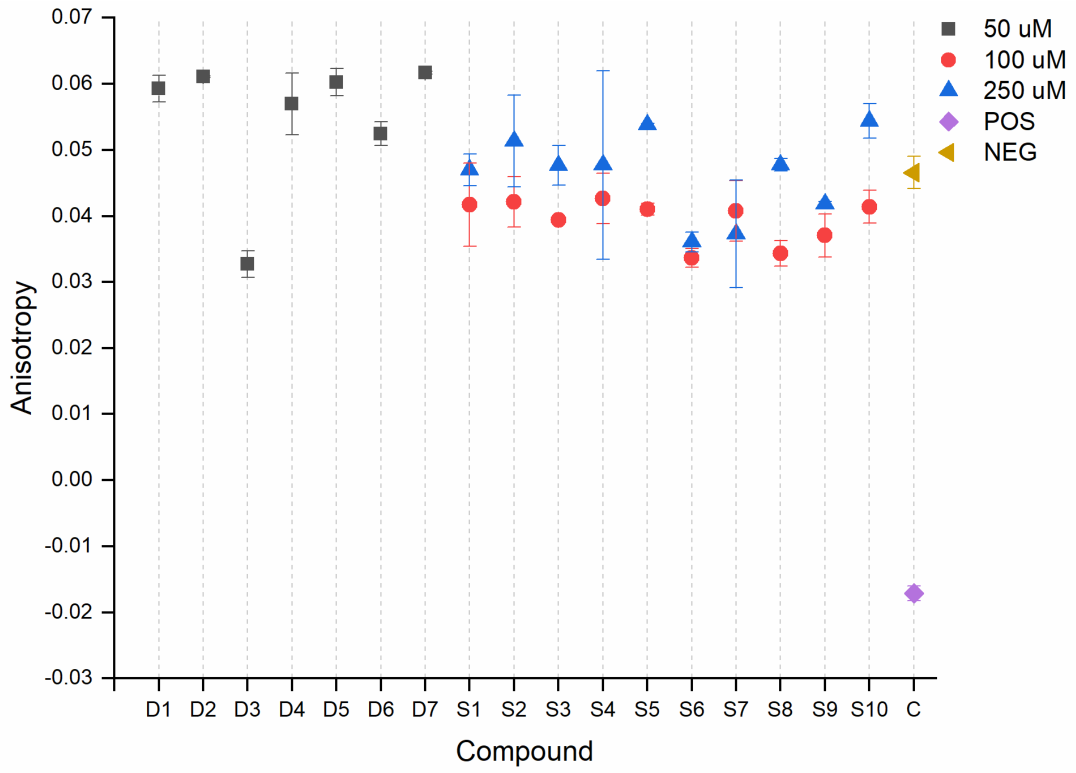

Round 2 HTS at 20 µM:

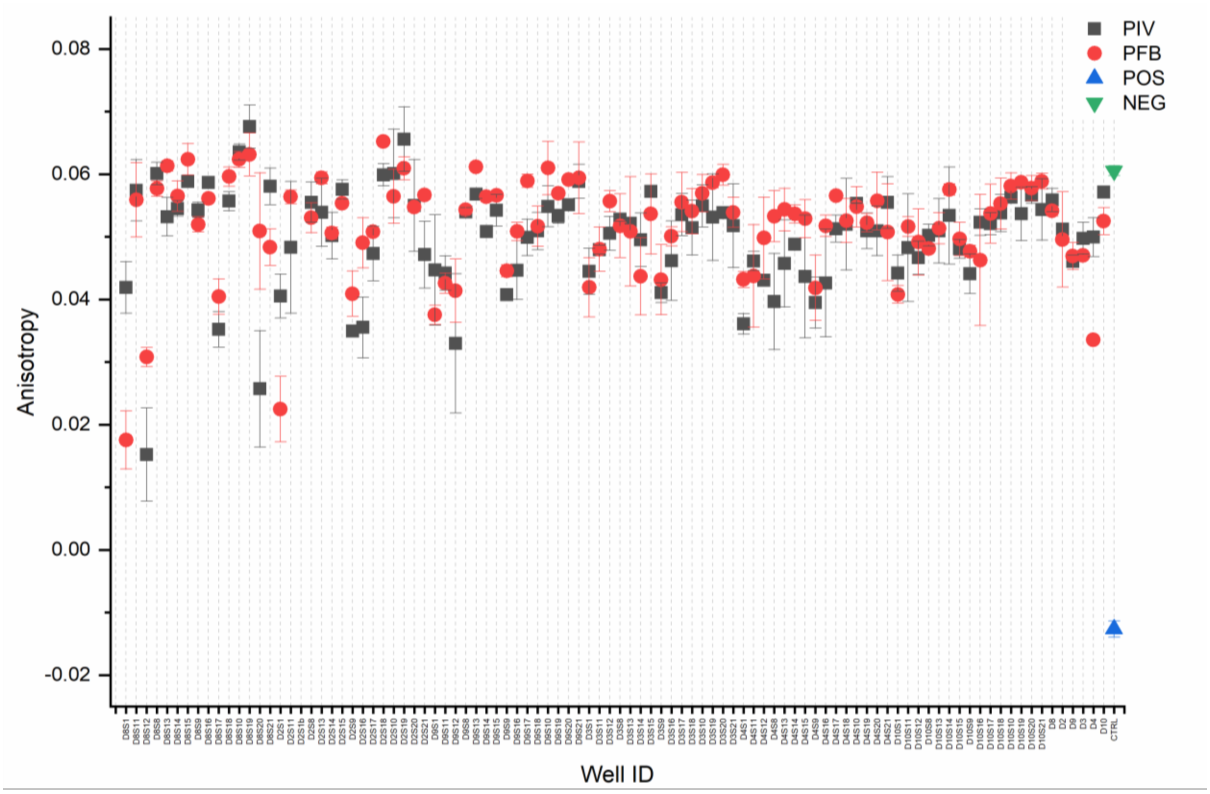

Reaction array 2 controls for individual reagents:

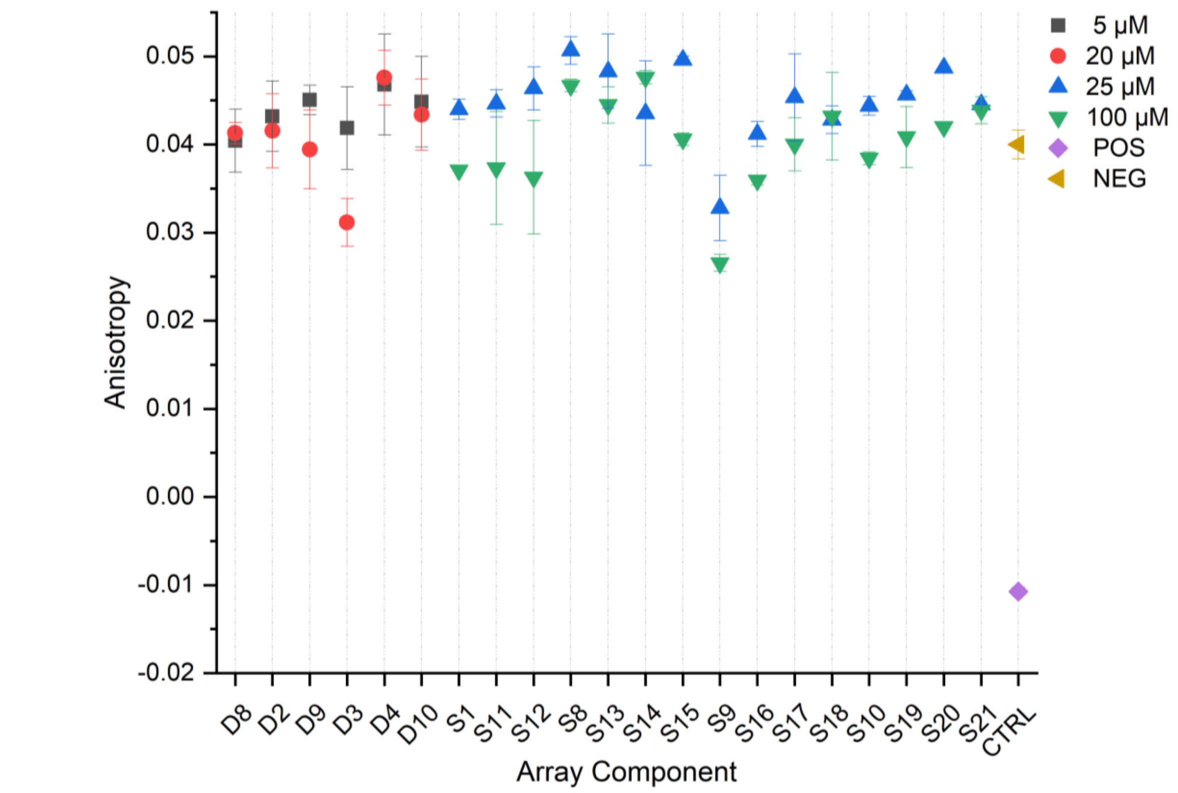

5.6 Determining IC_{50} values for isolated compounds

Pure compounds (**P1** – **P7** and **SI1** – **SI4**) were serially diluted in 100% DMSO (using 12 two-fold dilution steps) to achieve the correct effective concentrations (15.8 – 0.007 mM), then diluted 33-fold in pH 7.5 aqueous phosphate buffer (40 mM phosphate, 200 mM NaCl and 0.02 mg/mL Bovine Serum Albumin) to achieve a 3% DMSO intermediate stock solution (480 – 0.23 µM). The assay was then implemented similarly to the examples above to give final compound concentrations between 160 – 0.08 µM.

IC_{50} values were determined and curves were fit in Origin Pro 2019b using a non-linear curve fitting with the dose response fitting procedure and Levenberg Marquardt iteration algorithm.

P1:

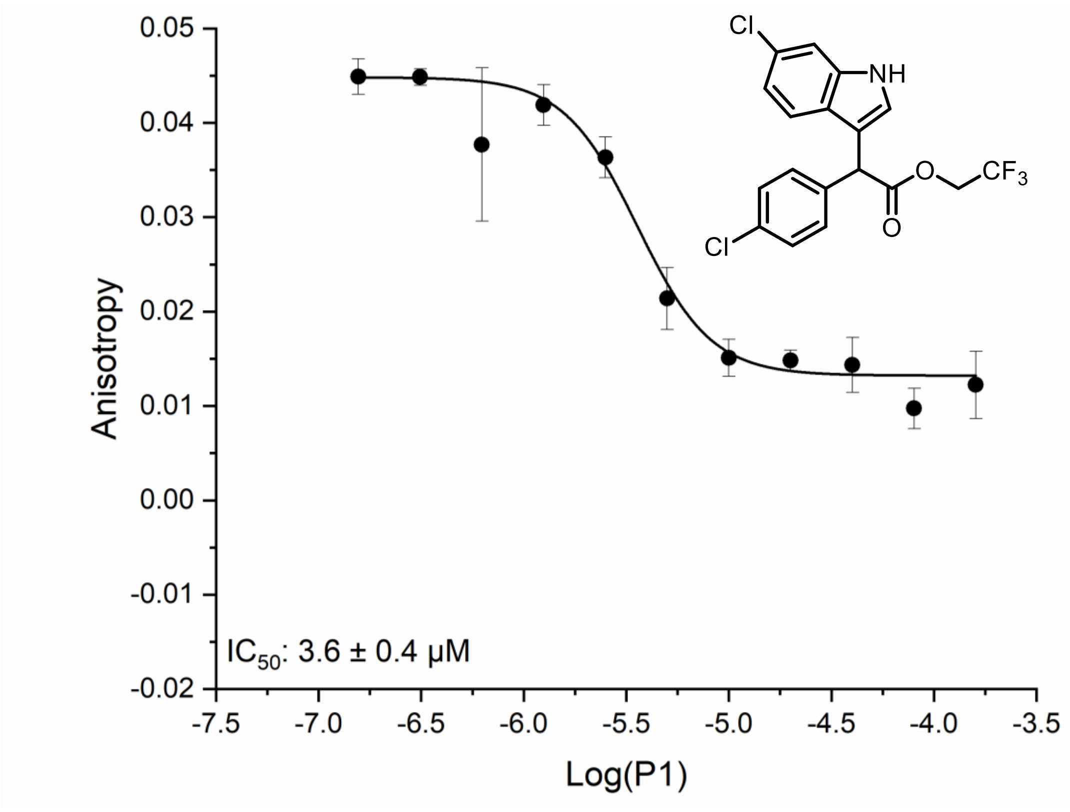

P2:

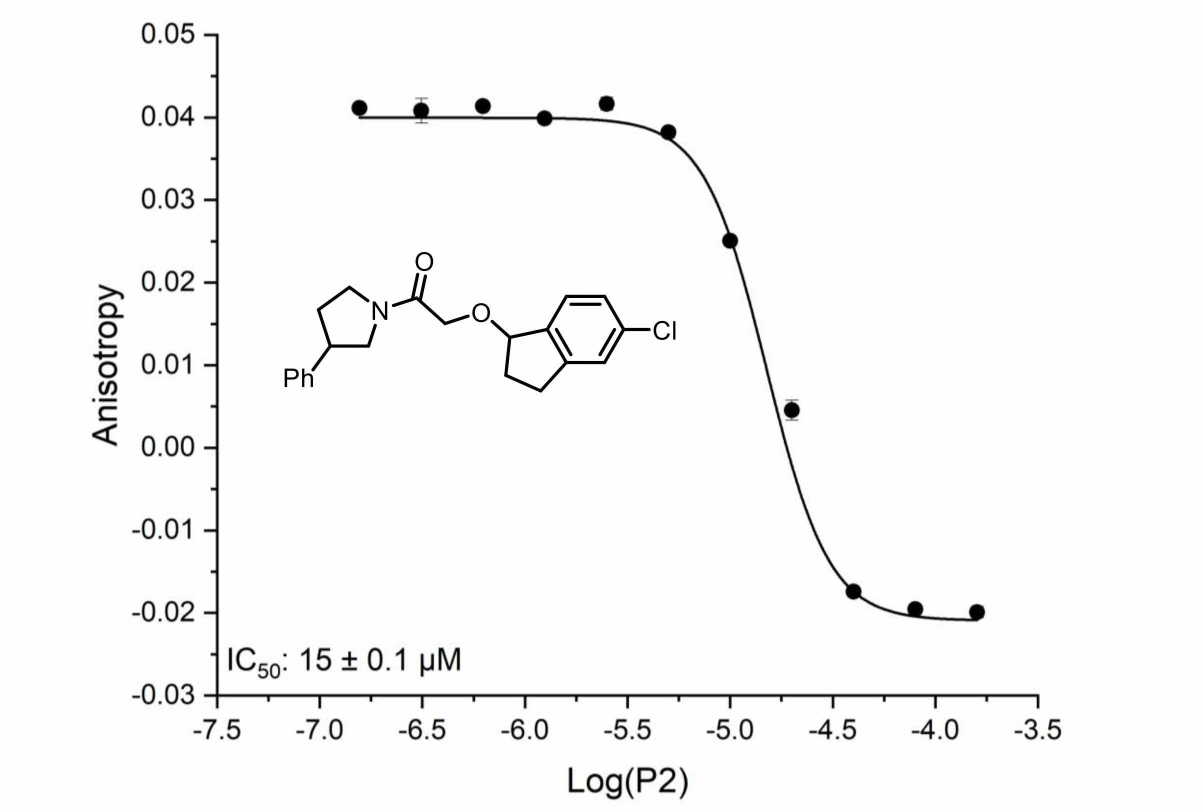

P2a and *ent*-**P2a**, **P2b** and *ent*-**P2b, SI1** and **SI2:**

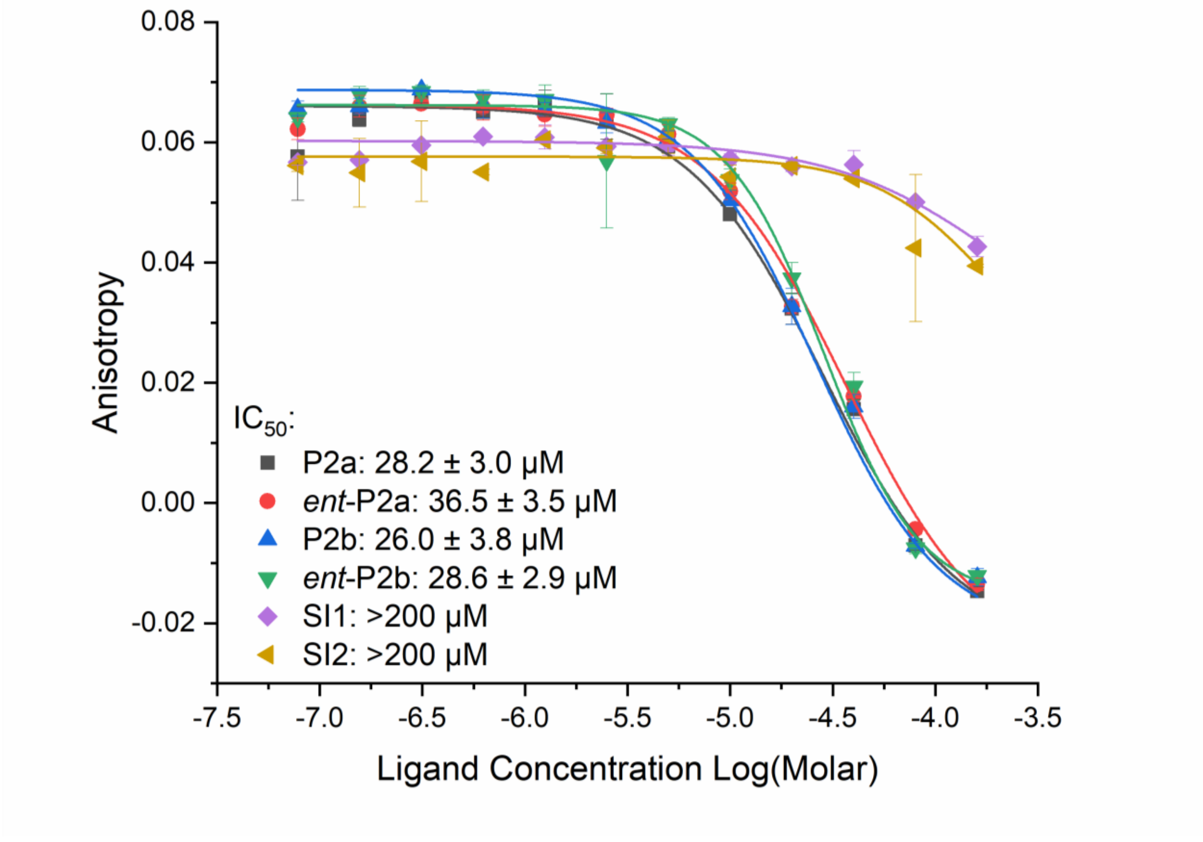

Table S1. Summary of measured IC_{50} values for **P2** diastereomers and analogues **SI1** and **SI2**.

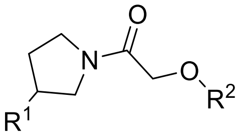

No.	R^1	R^2	Isomer	FA IC_{50} (μM)
**P2a**	Ph	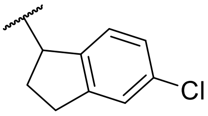	*S,R*	28.2 ± 3.0
**P2b**	Ph	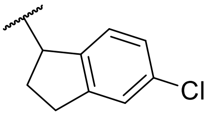	*S,S*	36.5 ± 3.5
ent-**P2a**	Ph	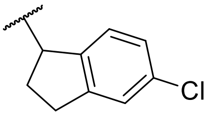	*R,S*	26.0 ± 3.8
ent-**P2b**	Ph	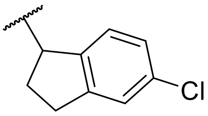	*R,R*	28.6 ± 2.9
**SI1**	Ph	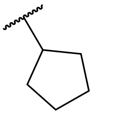	-	>200
**SI2**	H	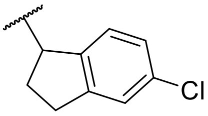	-	>200

P3

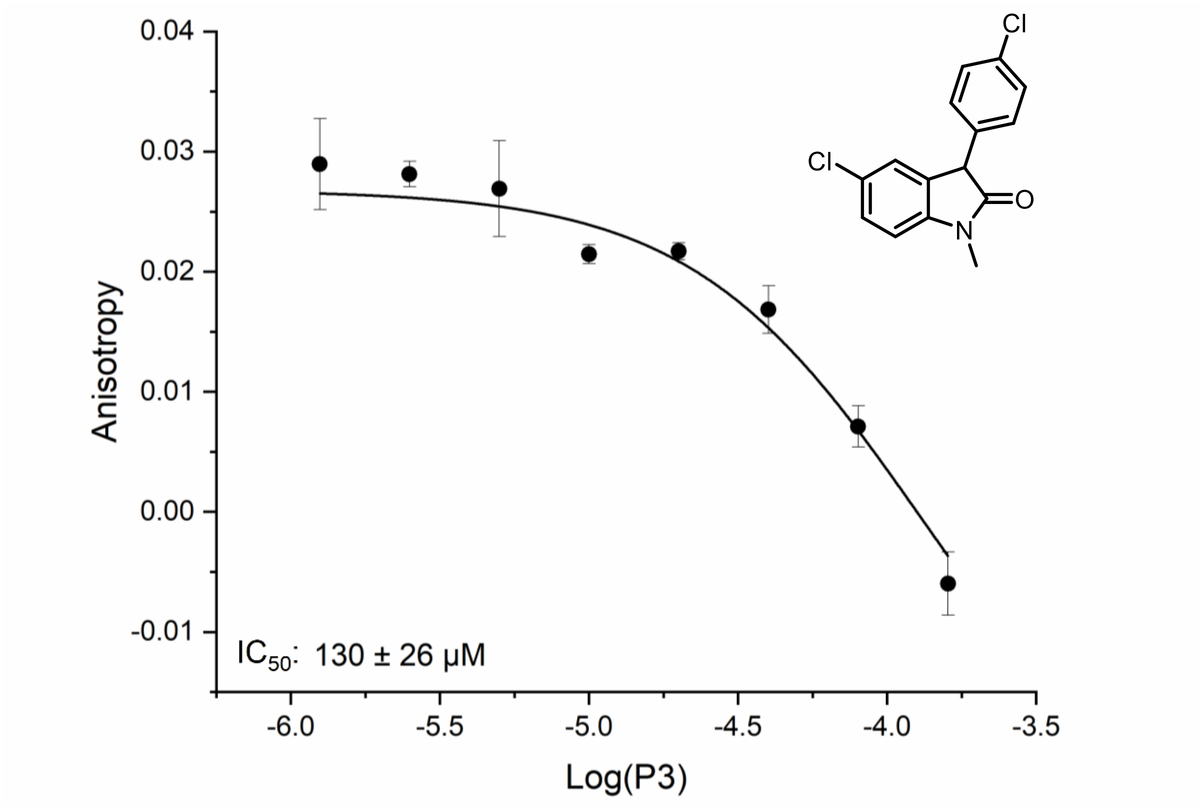

Due to poor compound solubility a full dose-response curve could not be obtained.

P4

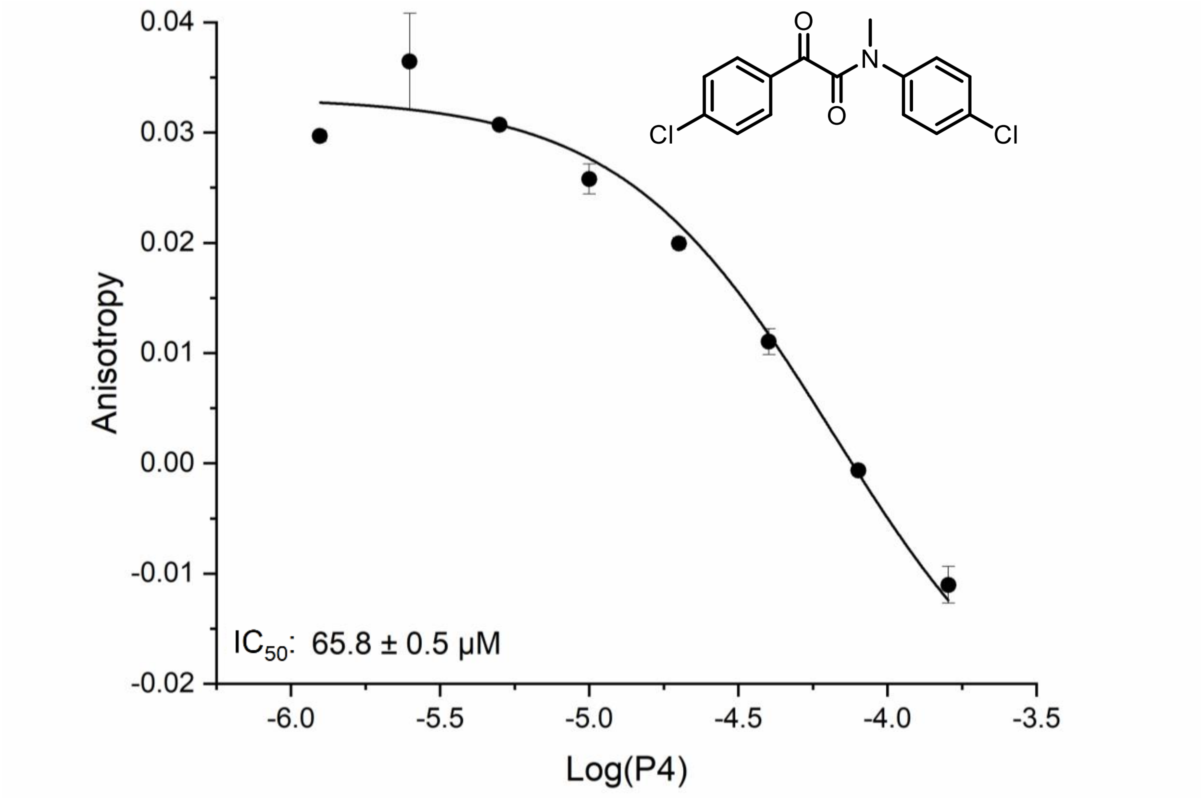

Due to poor compound solubility a full dose-response curve could not be obtained.

P5

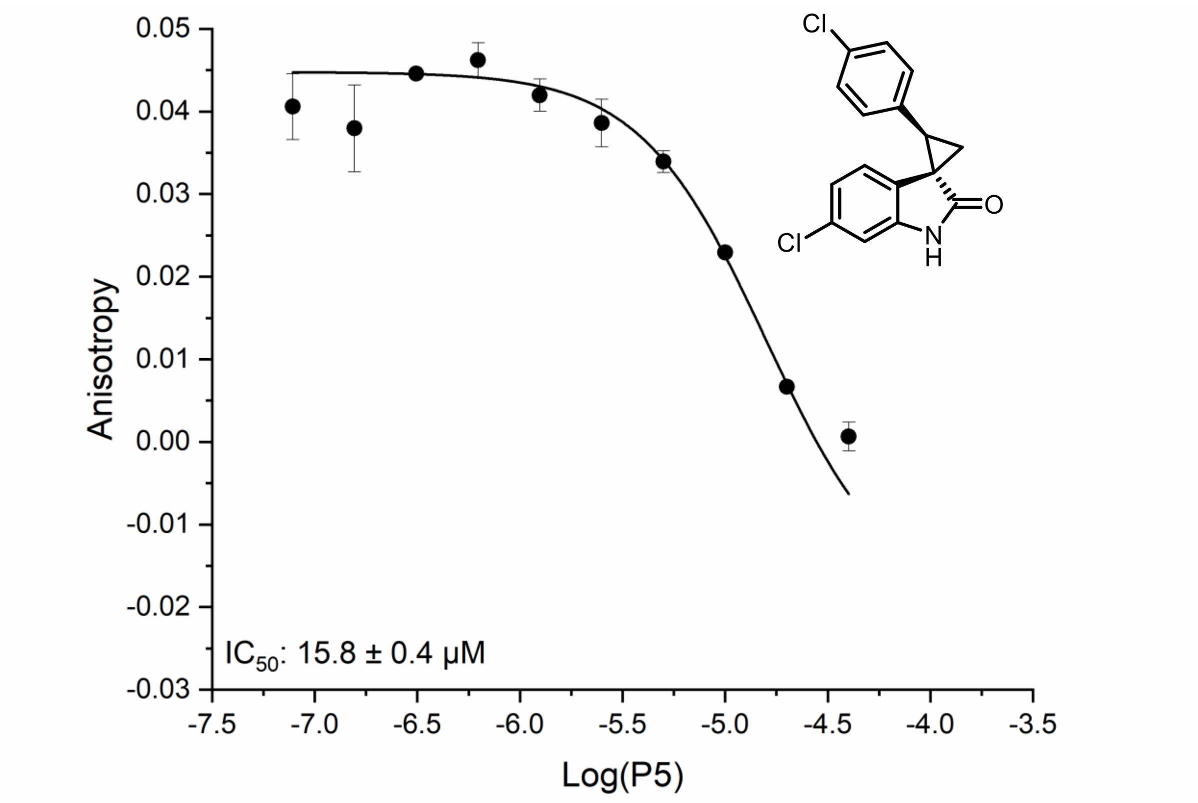

Due to poor compound solubility a full dose-response curve could not be obtained.

SI3

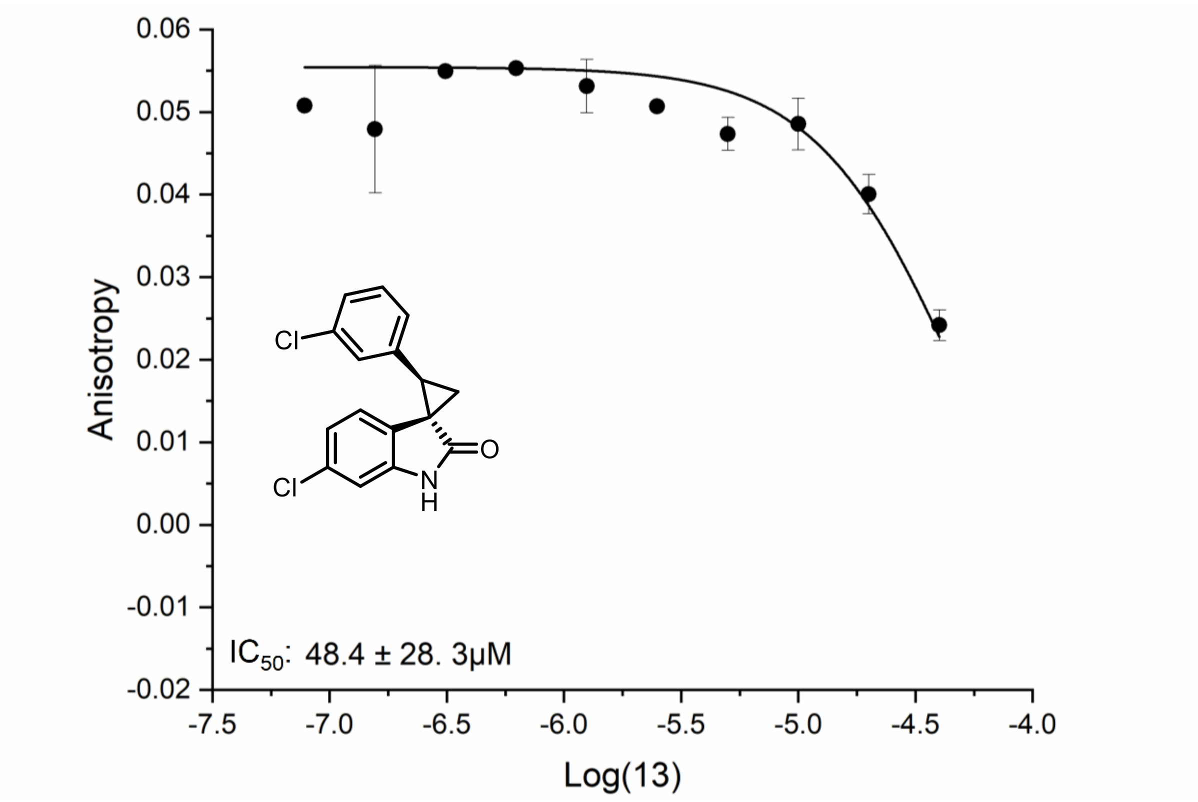

Due to poor compound solubility a full dose-response curve could not be obtained.

P6

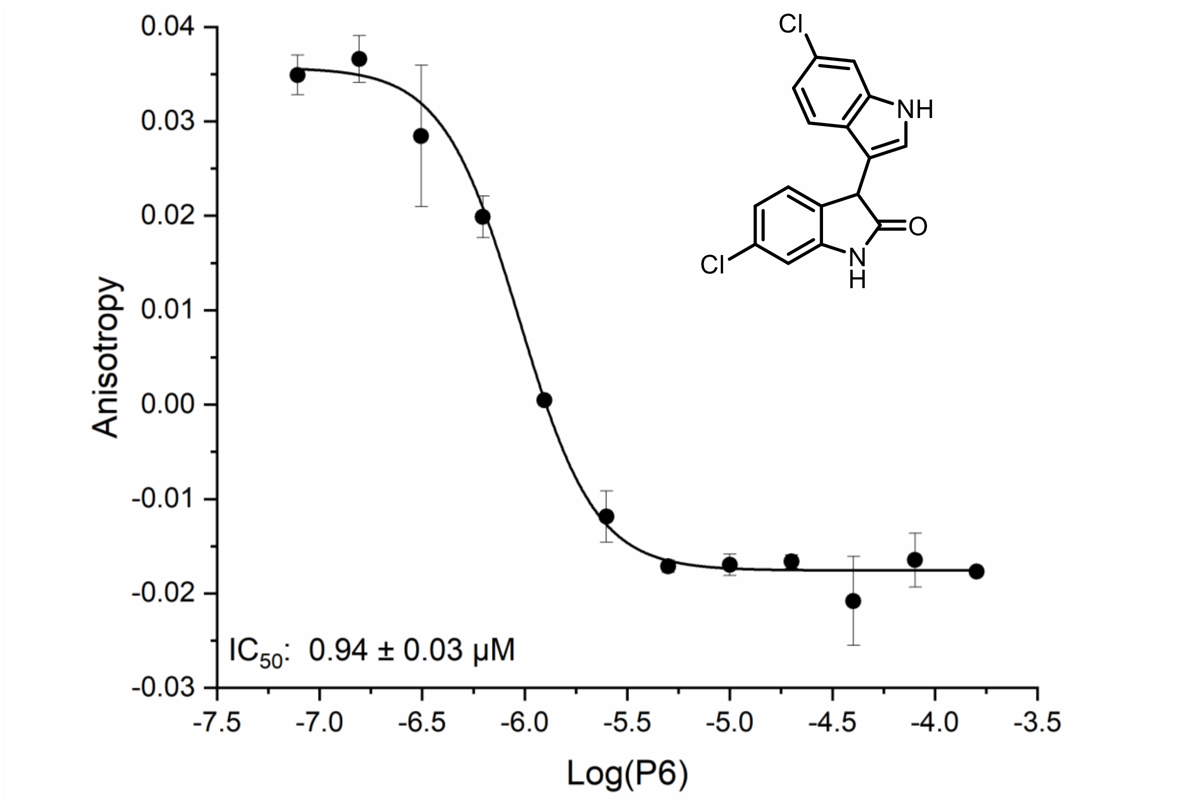
Anisotropy
Log(P6)
IC50: 0.94 ± 0.03 μM
Cl
NH
O
N
H

SI4

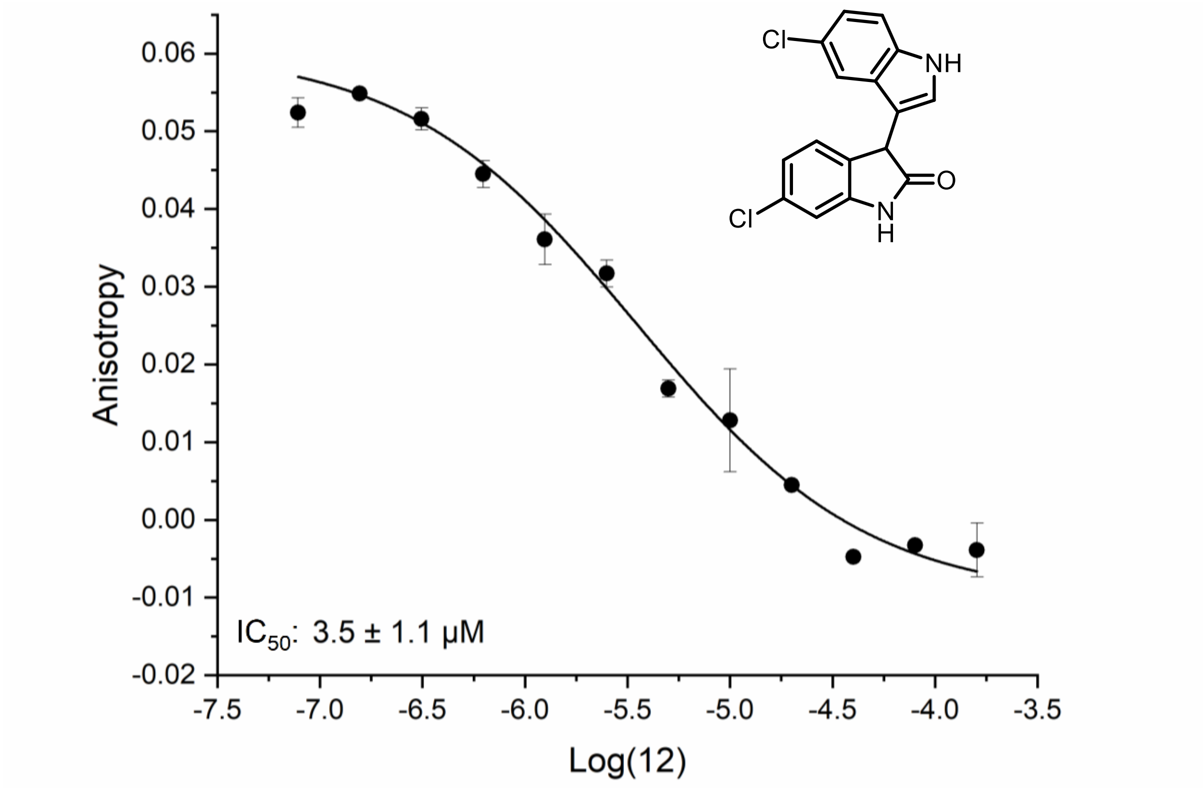
Anisotropy
Log(12)
IC50: 3.5 ± 1.1 μM
Cl
NH
O
N
H

P7

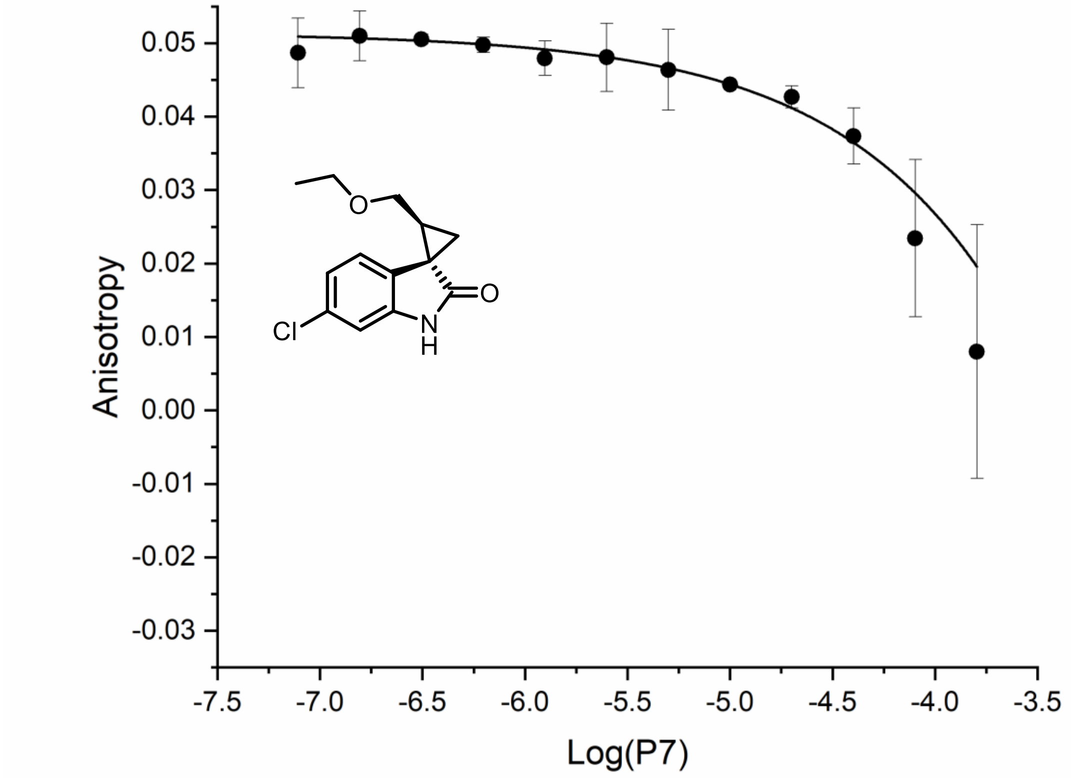

Due to poor compound solubility a full dose-response curve could not be obtained. Curve fitting failed and EC_{50} could not be determined.

6. NMR Measurements for K_d estimation by ^{15}N HSQC NMR

NMR titrations were performed by recording a series of $^{1}H/^{15}N$-HSQC experiments on a 750 MHz Oxford Magnet spectrometer (TCI-Cyroprobe, ^{1}H optimized triple resonance NMR 'inverse' probe) (^{1}H = 750 MHz and ^{15}N = 76 MHz) in pH 7.5 aqueous phosphate buffer containing 100 mM phosphate, 1 mM DTT and 2.5% glycerol with 50 µM ^{15}N-labelled $hDM2_{17\text{-}125}$, 10% D_2O and 1% DMSO. Temperature was maintained at 298 K throughout the experiments. Pure compounds were titrated into the ^{15}N-$hDM2_{17\text{-}125}$ sample in 0.5-, 1-, 1.5- and 2-molar equivalents relative to ^{15}N- $hDM2_{17\text{-}125}$ as standard and further molar equivalents of 4- and 6-times compound-to- $hDM2_{17\text{-}125}$ were added if the protein was not fully saturated. Data was processed using Topspin and analysed with Sparky.[6]

K_d values were obtained by plotting the observed chemical shift perturbation (csp) of the reporter peaks L54, L57, G58, M62, V75, V93, K94, H96 and K98 against the molar ratio of ligand. The csp of each reporter peak was calculated as the deviation from the free protein resonances using equation 6.

Eq.6: $$csp = \sqrt{(\omega_{2\,free} - \omega_{2\,bound})^2 + \frac{(\omega_{1\,free} - \omega_{1\,bound})^2}{10}}$$

Where ω_1 is the ^{15}N chemical shift and ω_2 is the ^1H chemical shift corresponding to the observed HSQC cross-peak for a given reporter residue.

K_d values for each reporter peak were then obtained by solving equation 7.

Eq. 7: $$\Delta = \Delta_o \frac{(K_d+[L]+[P]) - \sqrt{((K_d+[L]+[P])^2 - 4[P][L])}}{2[P]}$$

Where Δ is the observed csp, Δ_o is the maximum csp, and [P] and [L] are the protein and ligand concentrations respectively. The global K_d was then obtained from the average K_d for the combined reporter peaks as shown in equation 8.

Eq. 8: $$Global\ K_d = \frac{\sum Log_{10}(iK_d)}{n}$$

Where *i* is the reporter peak and n is the number of reporter peaks.

6.1 Spectra and fitting

P1 – due to intermediate and slow exchange chemical shift perturbation K_d could not be estimated using the reporter peaks outlined above.

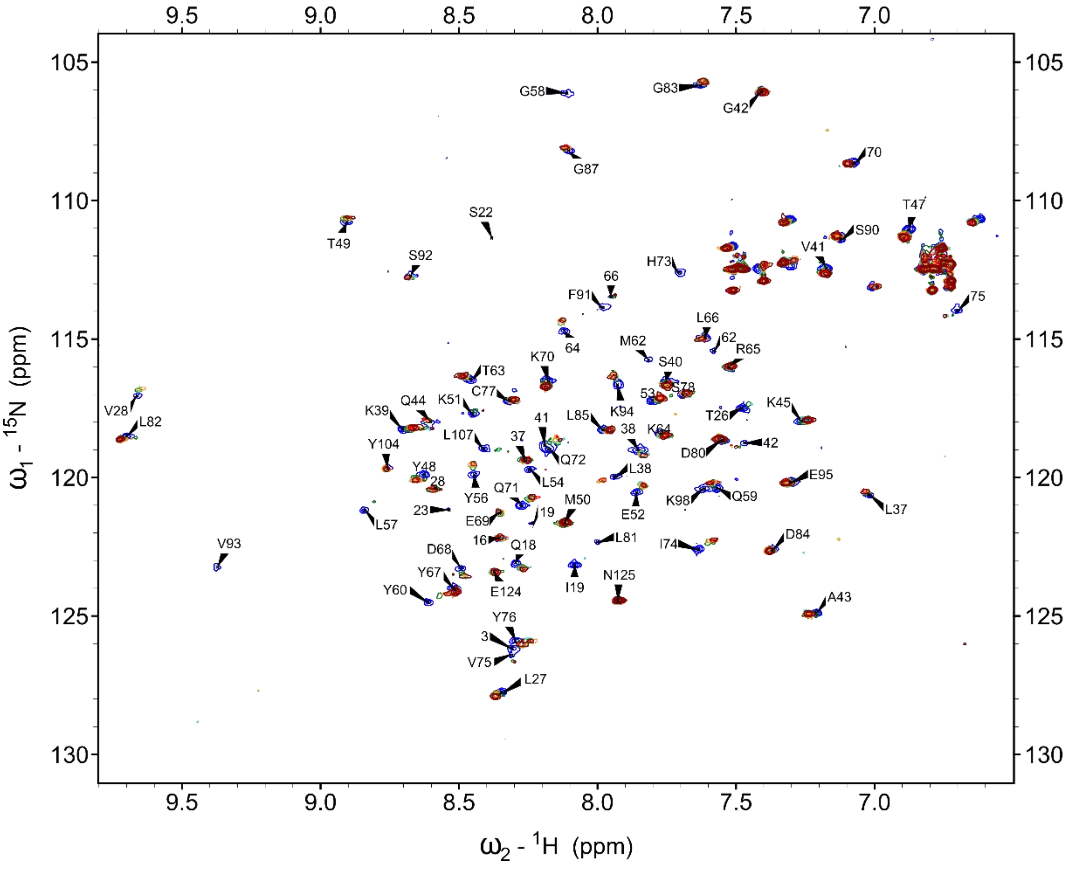

P2

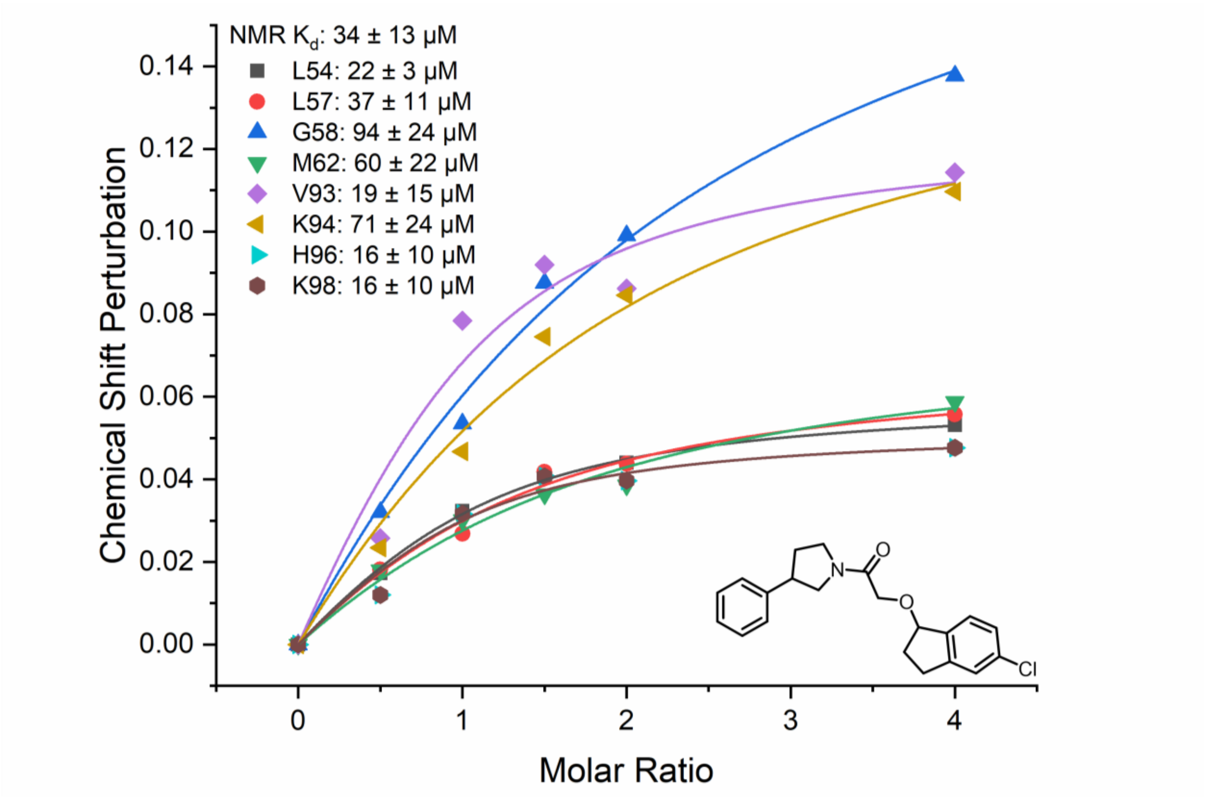
NMR K_d: 34 ± 13 μM
L54: 22 ± 3 μM
L57: 37 ± 11 μM
G58: 94 ± 24 μM
M62: 60 ± 22 μM
V93: 19 ± 15 μM
K94: 71 ± 24 μM
H96: 16 ± 10 μM
K98: 16 ± 10 μM
Chemical Shift Perturbation
Molar Ratio
0.14
0.12
0.10
0.08
0.06
0.04
0.02
0.00
0
1
2
3
4
N
O
O
Cl

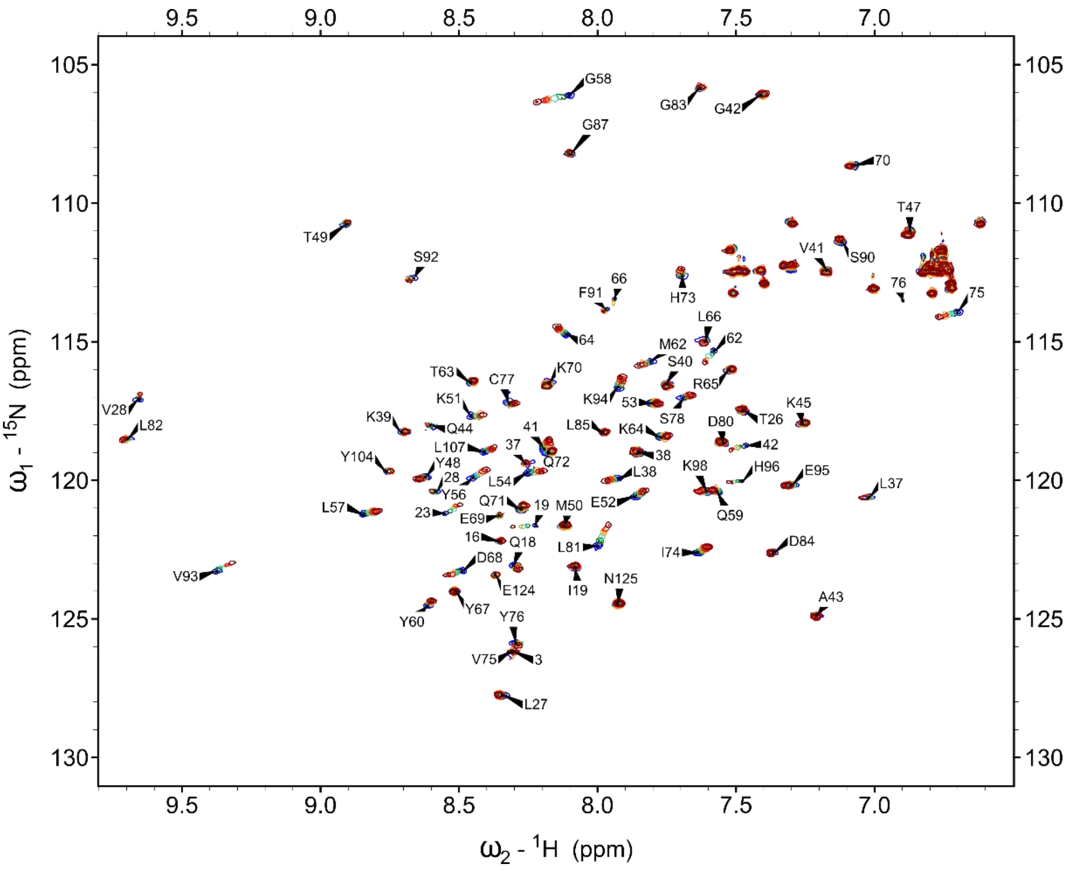
ω_1 - ^{15}N (ppm)
ω_2 - ^{1}H (ppm)
9.5
9.0
8.5
8.0
7.5
7.0
105
110
115
120
125
130
G58
G83
G42
G87
70
T47
T49
S92
V41
S90
66
F91
H73
76
75
L66
64
M62
62
S40
T63
C77
K70
R65
K94
53
K45
K51
T26
S78
D80
K39
Q44
L85
K64
41
42
V28
L82
L107
37
38
Y104
Y48
Q72
K98
H96
E95
28
L54
L38
Y56
L37
L57
Q71
19
M50
E52
Q59
23
E69
16
Q18
L81
I74
D84
D68
V93
N125
E124
I19
A43
Y67
Y60
Y76
V75
3
L27

P5

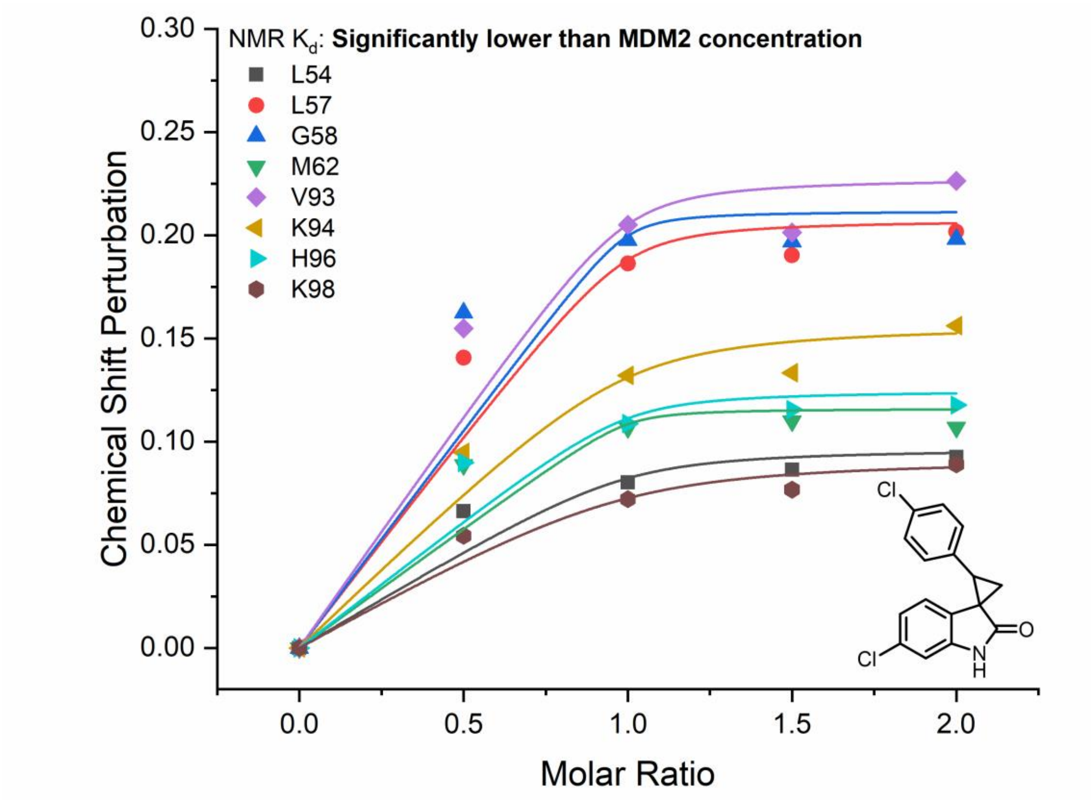
NMR K$_d$: Significantly lower than MDM2 concentration
L54
L57
G58
M62
V93
K94
H96
K98
Chemical Shift Perturbation
0.00
0.05
0.10
0.15
0.20
0.25
0.30
0.0
0.5
1.0
1.5
2.0
Molar Ratio
Cl
Cl
O
N
H

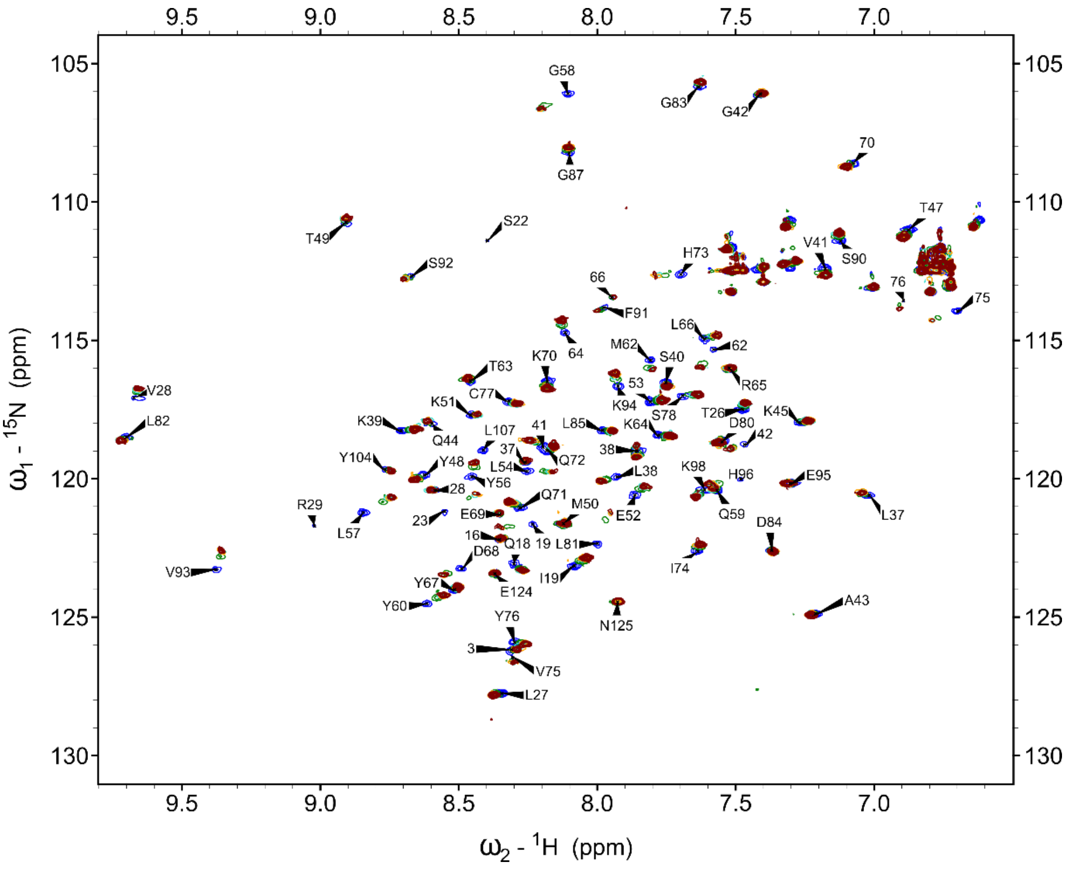
ω$_1$ - ^{15}N (ppm)
ω$_2$ - ^{1}H (ppm)
9.5
9.0
8.5
8.0
7.5
7.0
105
110
115
120
125
130
G58
G83
G42
G87
70
T47
S22
T49
S92
V41
S90
H73
66
76
75
F91
L66
M62
62
64
K70
S40
T63
53
R65
V28
C77
K51
K94
S78
T26
K45
L82
K39
L107
41
L85
K64
D80
Q44
42
Y104
Y48
37
Q72
38
L54
L38
K98
Y56
H96
E95
28
Q71
R29
M50
E52
Q59
L37
E69
23
L57
16
D84
Q18
19
L81
D68
V93
I74
I19
Y67
E124
A43
Y60
Y76
N125
3
V75
L27

P6

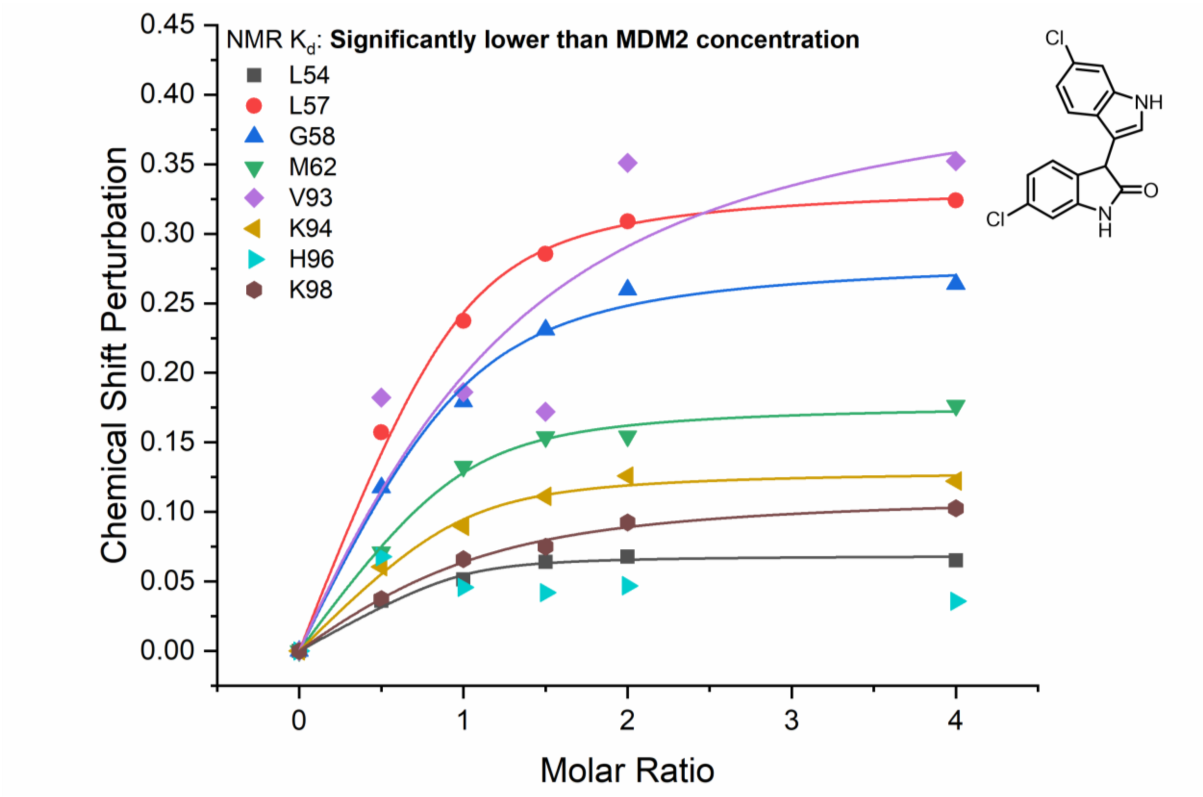

NMR K$_d$: Significantly lower than MDM2 concentration
L54
L57
G58
M62
V93
K94
H96
K98
Chemical Shift Perturbation
Molar Ratio
0.45
0.40
0.35
0.30
0.25
0.20
0.15
0.10
0.05
0.00
0
1
2
3
4
Cl
NH
O
N
H

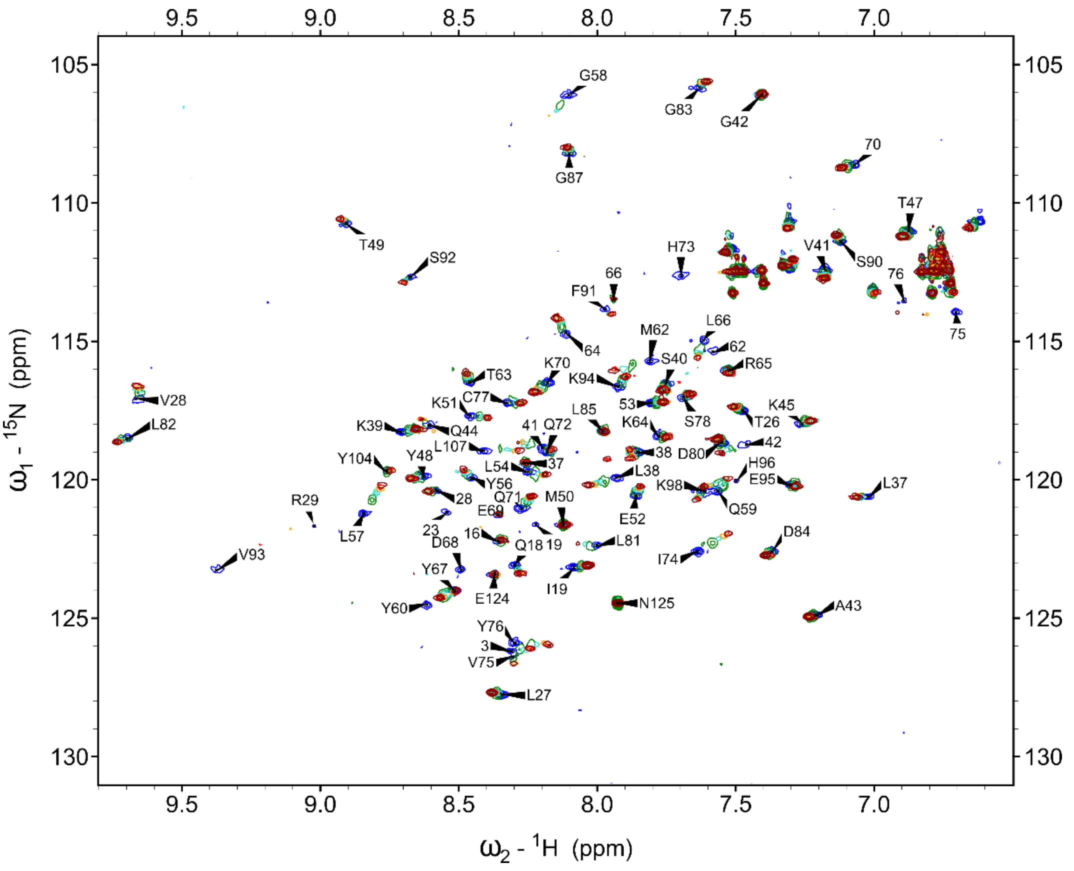

ω$_1$ - ^{15}N (ppm)
ω$_2$ - ^{1}H (ppm)
9.5
9.0
8.5
8.0
7.5
7.0
105
110
115
120
125
130
G58
G83
G42
70
G87
T47
T49
S92
H73
V41
S90
76
66
F91
75
M62
L66
64
S40
62
R65
T63
K70
K94
V28
L82
K51
C77
L85
53
K45
K39
Q44
41
Q72
K64
S78
T26
42
L107
38
D80
Y104
Y48
L54
37
H96
L38
E95
Y56
L37
28
K98
R29
Q71
M50
Q59
E69
E52
L57
23
D68
16
Q18
19
L81
D84
V93
I74
Y67
E124
I19
N125
A43
Y60
Y76
3
V75
L27

P4

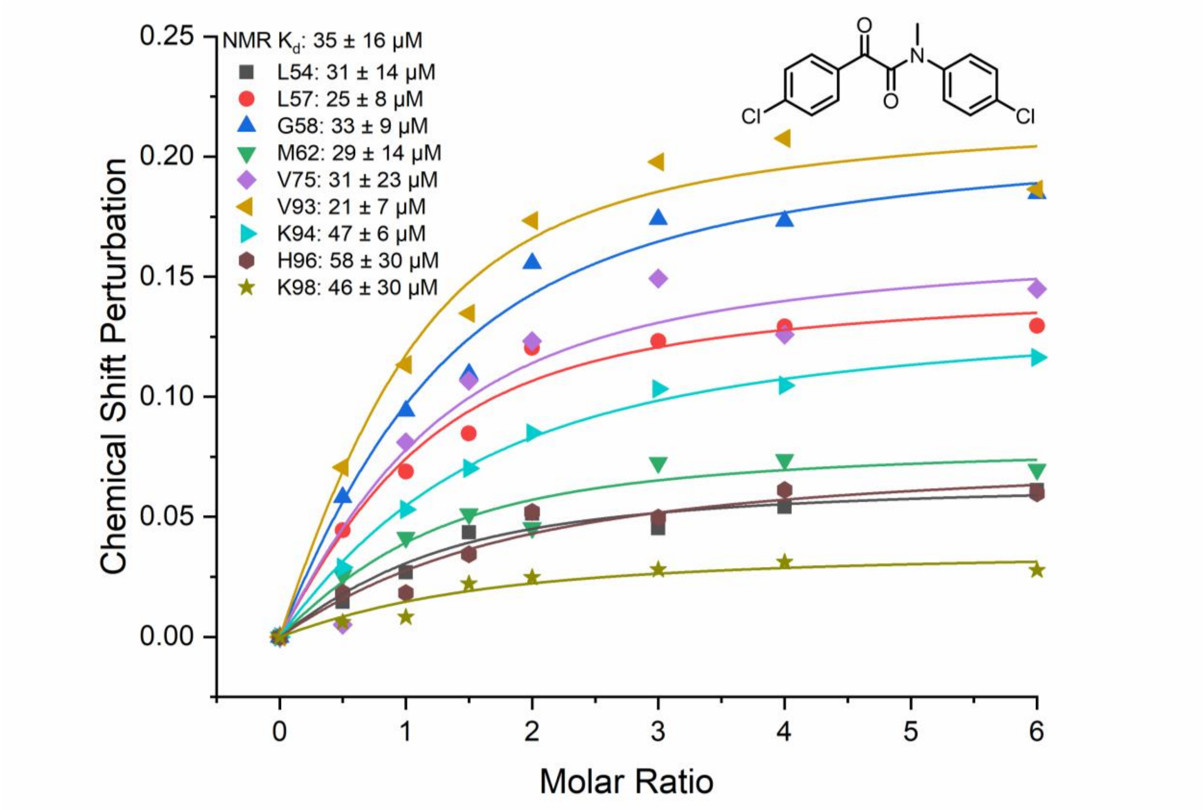
NMR K_d: 35 ± 16 μM
L54: 31 ± 14 μM
L57: 25 ± 8 μM
G58: 33 ± 9 μM
M62: 29 ± 14 μM
V75: 31 ± 23 μM
V93: 21 ± 7 μM
K94: 47 ± 6 μM
H96: 58 ± 30 μM
K98: 46 ± 30 μM
Chemical Shift Perturbation
Molar Ratio
Cl
Cl
O
O
N

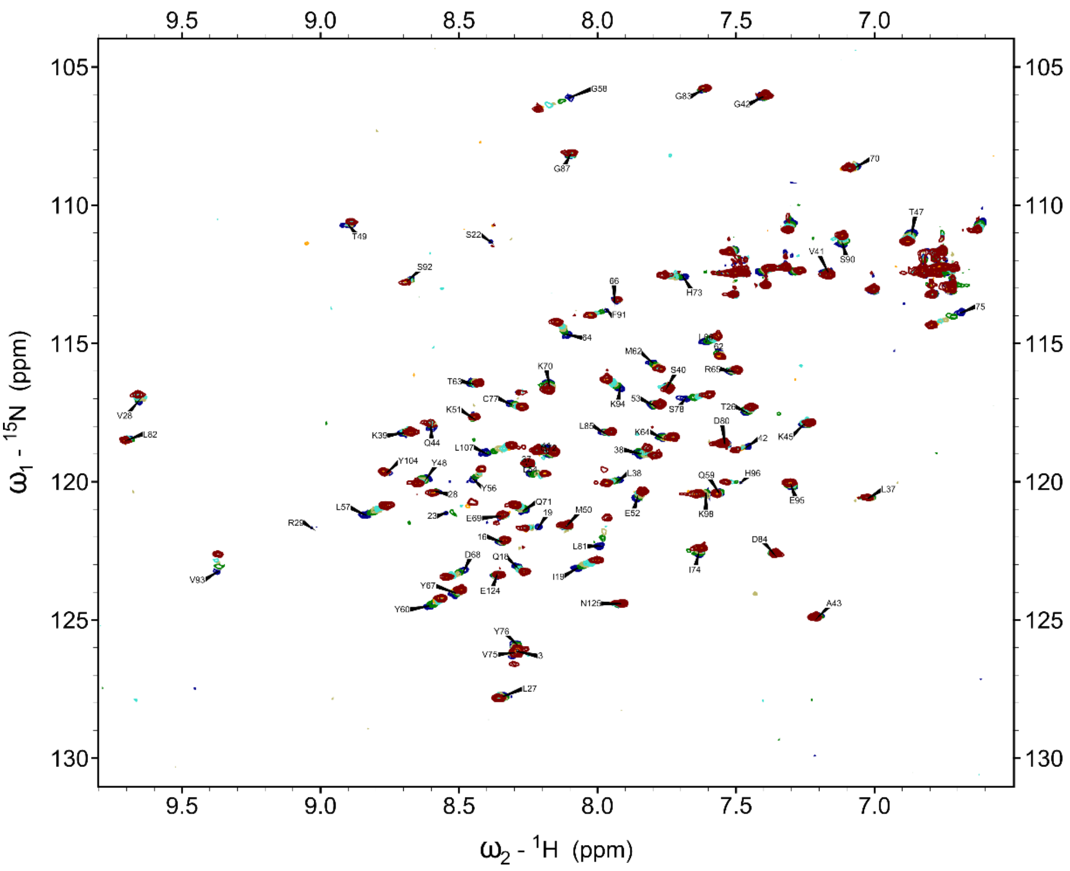
ω_1 - ^{15}N (ppm)
ω_2 - ^{1}H (ppm)

P3 – weak chemical shift perturbation observed up to a 6:1 molar ratio of **P3**/^{15}N-*h*DM2

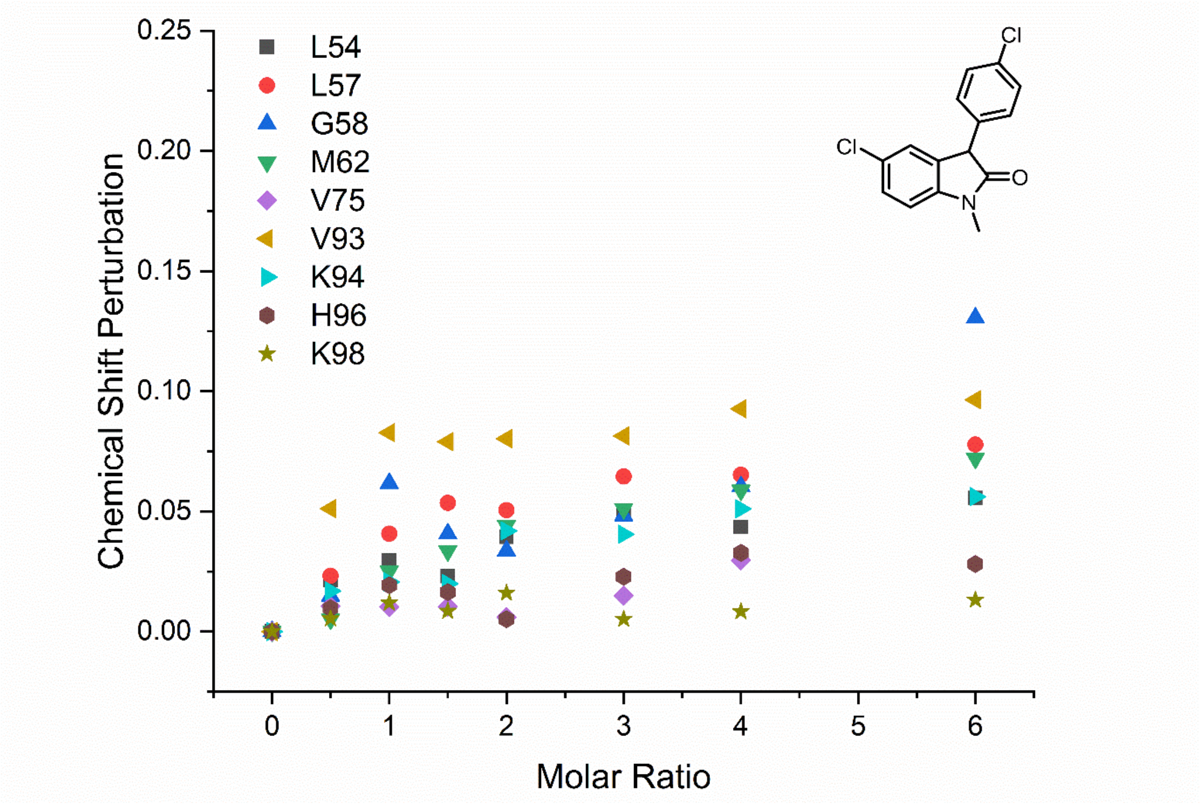

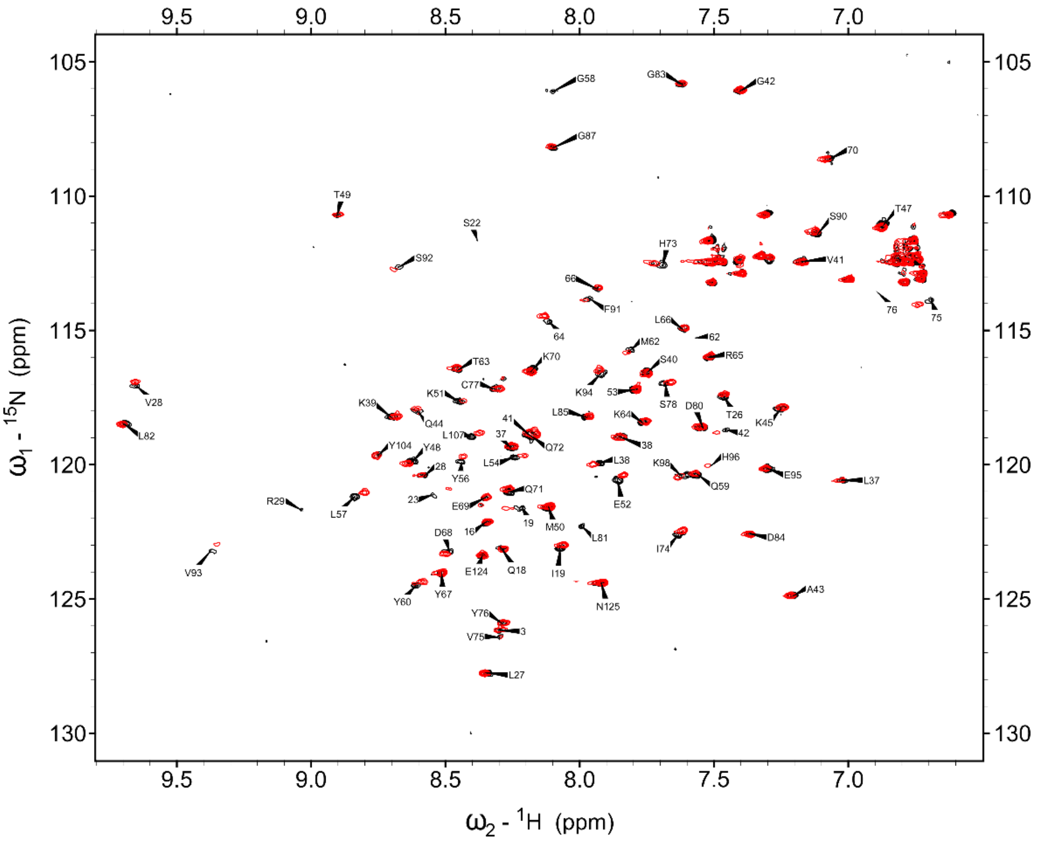

P7 – no chemical shift perturbation was observed up to a 6:1 molar ratio of **P7**/^{15}N-*h*DM2

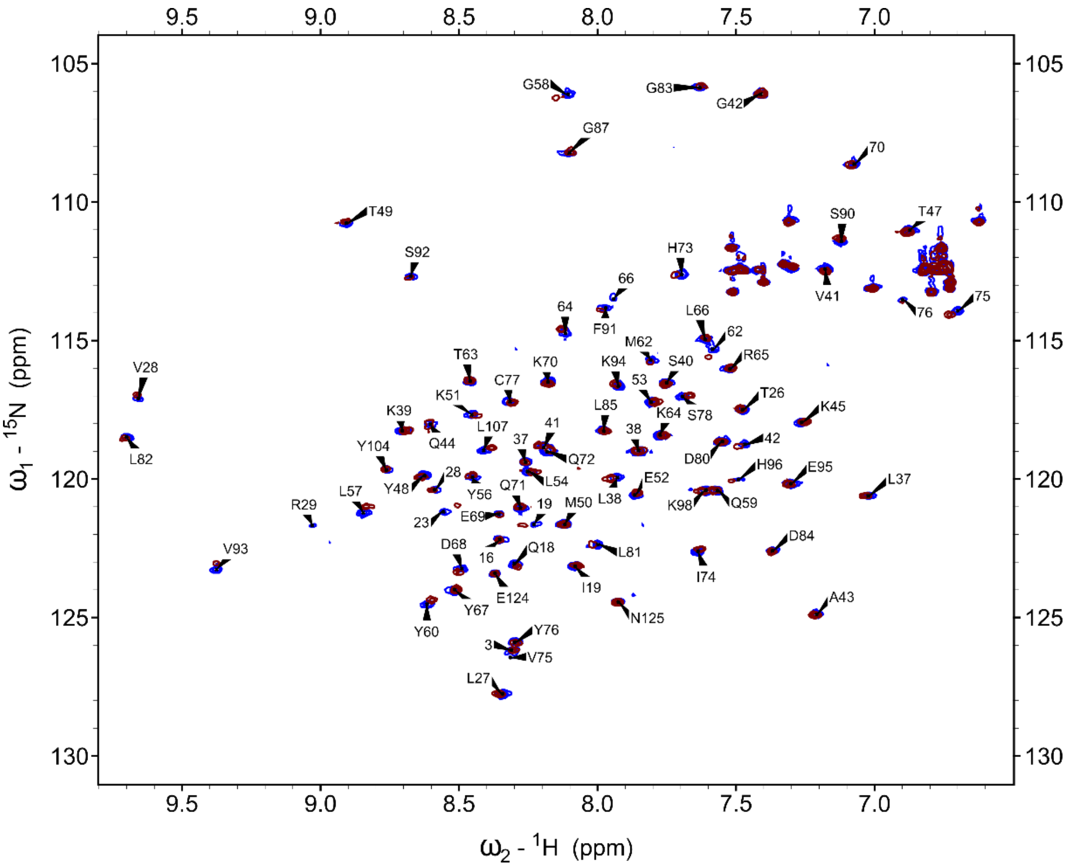

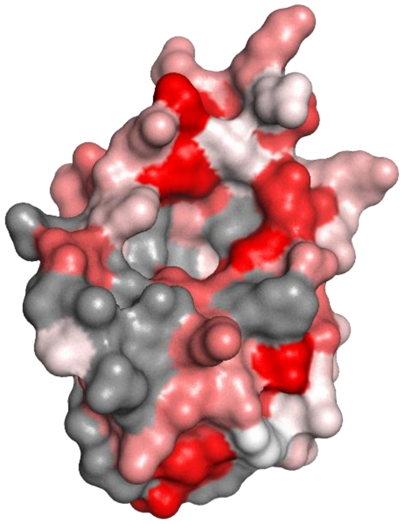

Figure S3. ^{15}N-H HSQC chemical shift perturbation of assigned peaks for 50 µM ^{15}N-labelled *h*DM2 on addition of Nutlin-3a (100 µM). Unassigned residues are highlighted in grey.

6.2 Counter-screening of P1, P2, P5 and P6 against MCL-1

^{15}N-labelled MCL-1 was expressed using the procedure reported by Wilson *et. al.*[1e,1f] using M9 minimal media enriched with $^{15}NH_4Cl$ as nitrogen source.

Single point NMR screens were performed by recording two ^{1}H/^{15}N-HSQC experiments on a 600 MHz Oxford Magnet spectrometer (QCI-P-Cyroprobe, ^{1}H optimized quadruple resonance NMR 'inverse' probe) (^{1}H = 600 MHz and ^{15}N = 61 MHz) in pH 7.4 aqueous buffer containing 100 mM phosphate, 1 mM DTT and 2.5% glycerol, with 50 µM ^{15}N-labelled MCL-1, 10% D_2O and 1% DMSO. Temperature was maintained at 298 K throughout the experiments. Pure compounds were added into the ^{15}N- MCL-1 sample as one 6 molar equivalent relative to ^{15}N- MCL-1 as standard. Data was processed using Topspin and analysed with Sparky.[6]

P1 – Black cross peaks: free ^{15}N- MCL-1 and red cross peaks: 6:1 molar ratio of **P1**/^{15}N- MCL-1.

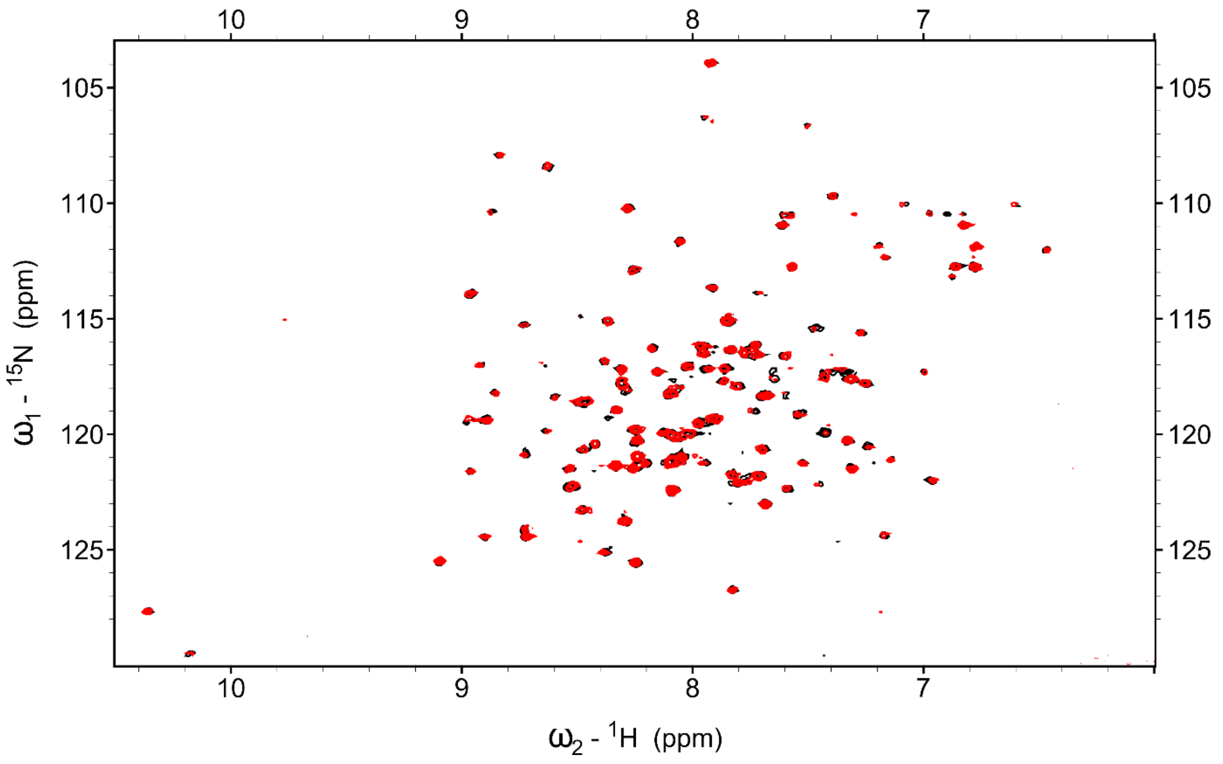

P2 – Black cross peaks: free ^{15}N- MCL-1 and red cross peaks: 6:1 molar ratio of **P2**/^{15}N- MCL-1.

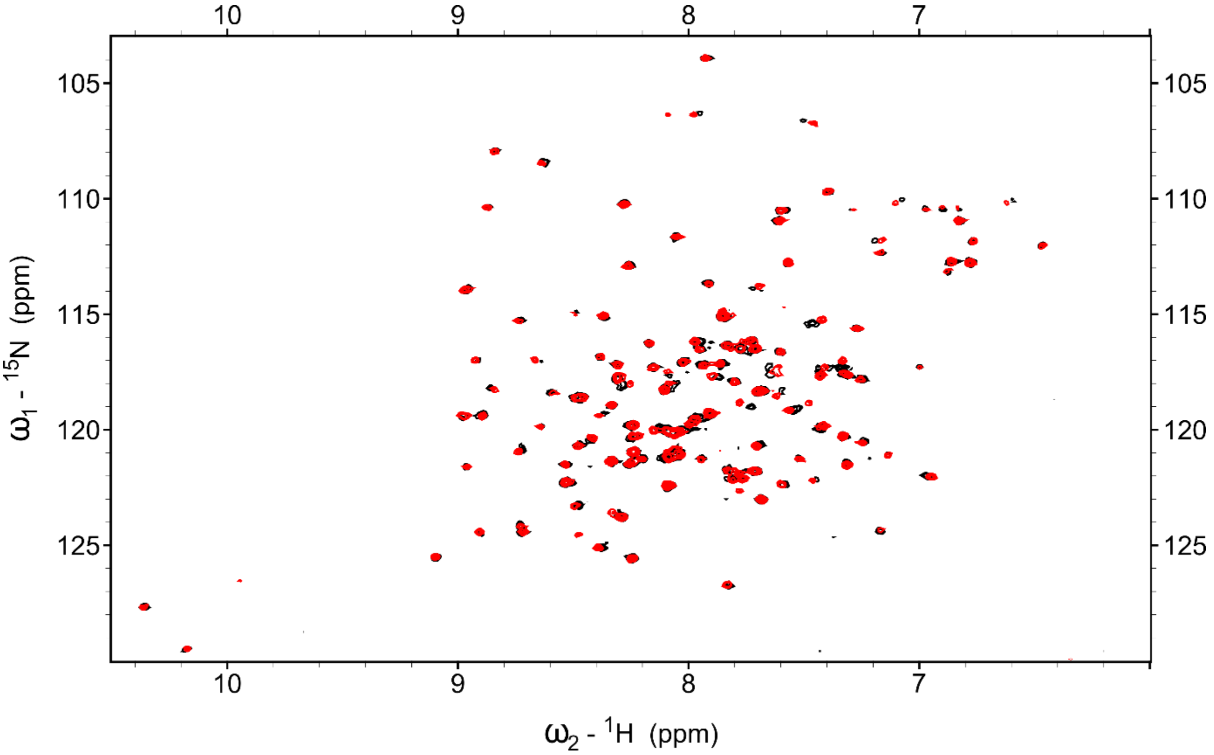

P5 – Black cross peaks: free ^{15}N- MCL-1 and red cross peaks: 6:1 molar ratio of **P5**/^{15}N- MCL-1.

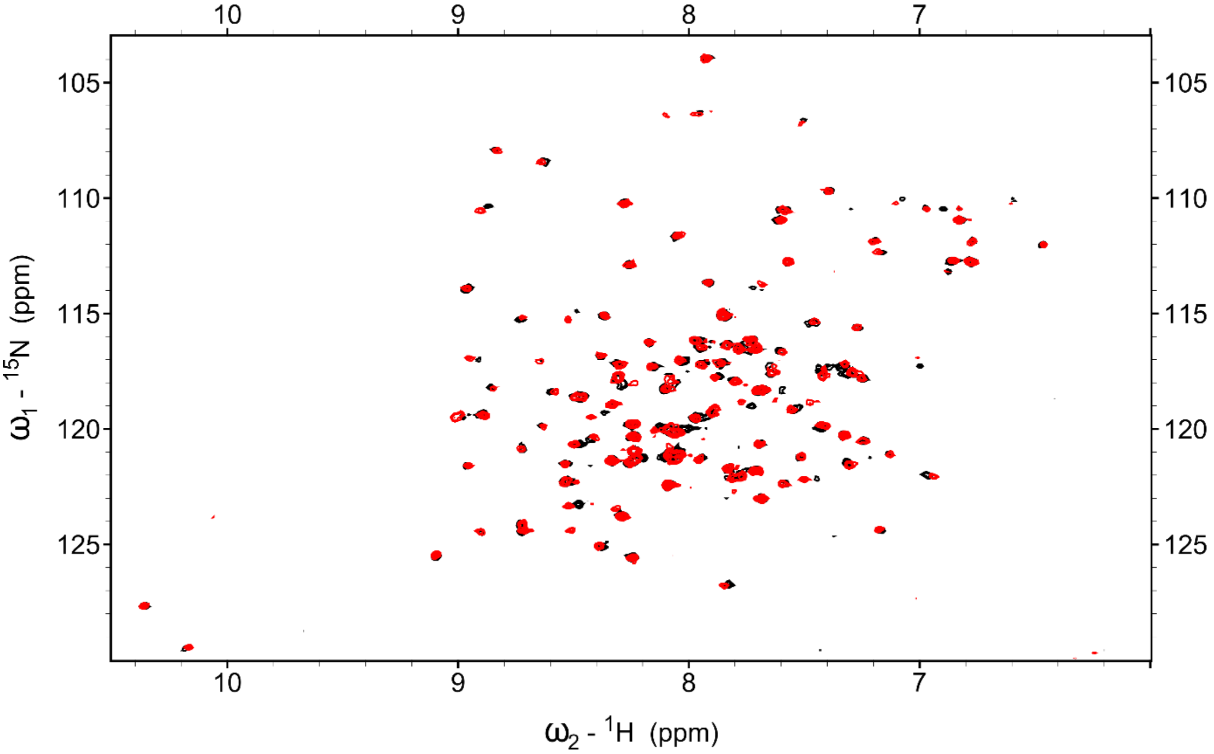

P6 – Black cross peaks: free ^{15}N- MCL-1 and red cross peaks: 6:1 molar ratio of **P6**/^{15}N- MCL-1.

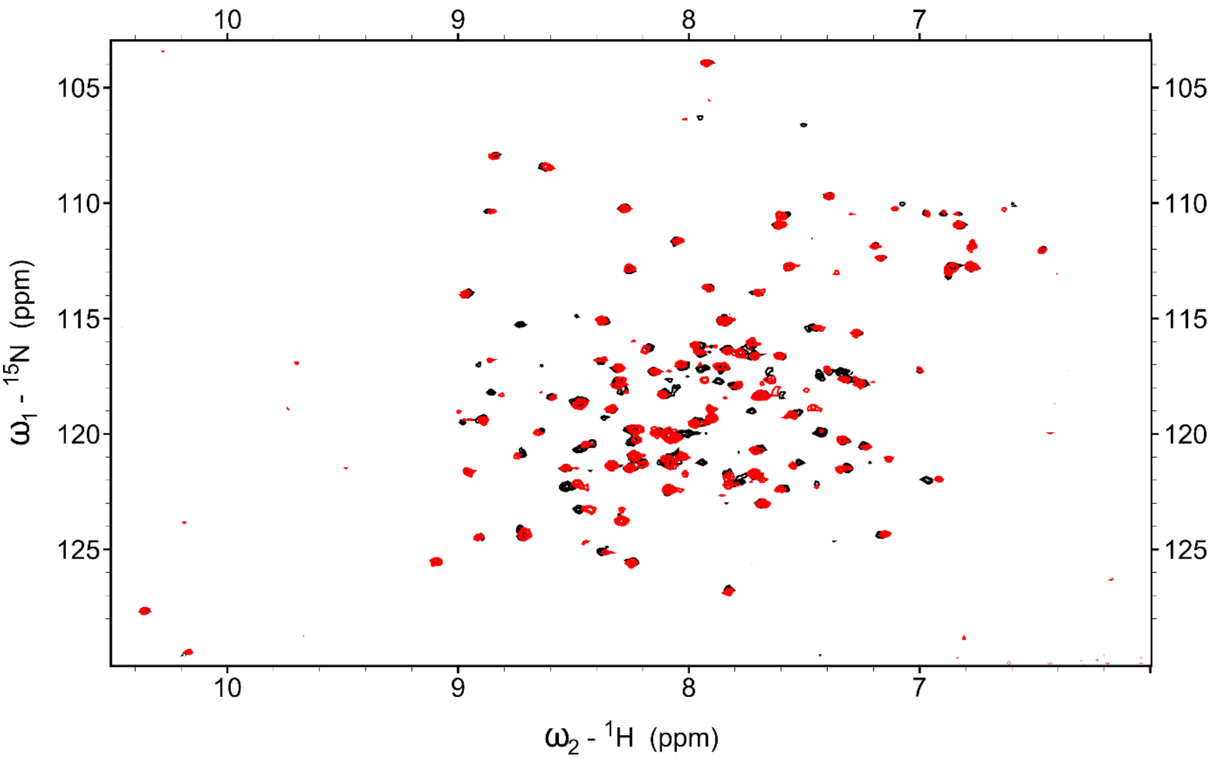

7. LC-MS Analysis of Reaction Mixtures

All 154 reactions from the round 1 reaction array were analysed by LC-MS to investigate how many combinations had produced a desired product (Figure S4). All samples were diluted to 1 mg/mL concentrations from the original 50 mM DMSO master stock, with respect to the initial diazo starting concentration. Reaction wells containing diazo and substrate were analysed for intermolecular product(s) and blank control wells were analysed for intramolecular product(s). Dark green squares indicate clear *m/z* for the desired product(s) and a clear corresponding UV peak(s). Light green squares indicate *m/z* for the desired product(s) and either weak or no corresponding UV peak(s). Blank squares indicate that no *m/z* was observed for the desired product(s).

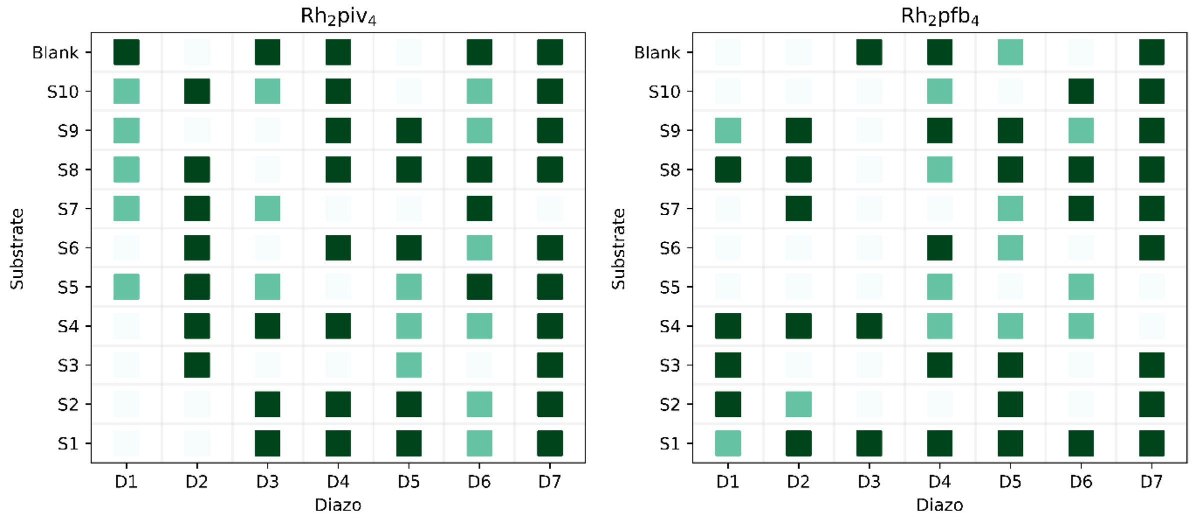

Figure S4. LC-MS heatmap for reaction array one.

Overall, 112 out of 154 reactions (73%) showed the presence of an expected product *m/z*, by LC-MS, of which 78 reactions also showed distinct UV peaks. For all 70 combinations of diazo and co-substrate, excluding blank intramolecular controls, only 8 combinations out of 70 (11%) failed to give detectable product *m/z* when considering reactions across both catalysts. This demonstrates considerable sampling of the available chemical space across the first reaction array.

Table S2. LC-MS data from the round 1 reaction array

Diazo/ Substrate	Catalyst	Formula	Adduct	Expected	Found	Peak Intensity	UV peak?
D1S1	Rh_2piv_4	C19H16Cl2N2O2	-	374.06	-	-	N
D1S2	Rh_2piv_4	C17H15Cl2NO3	-	351.04	-	-	N
D1S3	Rh_2piv_4	C25H22Cl3NO2	-	473.07	-	-	N
D1S4	Rh_2piv_4	C18H21ClN2O4	-	364.12	-	-	N
D1S5	Rh_2piv_4	C21H25ClN2O3	H	389.16	389.16	5x10^6	N
D1S6	Rh_2piv_4	C20H18ClNO3	-	355.10	-	-	N
D1S7	Rh_2piv_4	C22H23ClN2O2	H	383.15	383.15	1x10^7	N
D1S8	Rh_2piv_4	C20H19Cl2NO3	H	392.07	392.08	5X10^6	N
D1S9	Rh_2piv_4	C20H17Cl2NO2	H	374.06	374.07	5x10^6	N
D1S10	Rh_2piv_4	C15H18ClNO4S	H	344.07	344.07	3x10^6	N
D1blank	Rh_2piv_4	C11H10ClNO2	2M + 2H	448.10	448.64	7.5x10^7	Y
D1S1	Rh_2pfb_4	C19H16Cl2N2O2	H	375.06	375.07	1x10^7	N
D1S2	Rh_2pfb_4	C17H15Cl2NO3	H	352.04	352.24	4x10^7	Y
D1S3	Rh_2pfb_4	C25H22Cl3NO2	Na	496.06	498.1	4x10^7	Y
D1S4	Rh_2pfb_4	C18H21ClN2O4	H	365.12	365.03	1x10^7	Y
D1S5	Rh_2pfb_4	C21H25ClN2O3	-	388.16	-	-	N
D1S6	Rh_2pfb_4	C20H18ClNO3	-	355.10	-	-	N
D1S7	Rh_2pfb_4	C22H23ClN2O2	-	382.15	-	-	N
D1S8	Rh_2pfb_4	C20H19Cl2NO3	Na	414.06	413.97	3x10^6	Y
D1S9	Rh_2pfb_4	C20H17Cl2NO2	H	374.06	374.07	2x10^7	N
D1S10	Rh_2pfb_4	C15H18ClNO4S	-	343.07	-	-	N
D1blank	Rh_2pfb_4	C11H10ClNO2	-	223.04	-	-	N
D2S1	Rh_2piv_4	C18H12Cl2F3NO2	H	402.02	401.98	3x10^6	N
D2S2	Rh_2piv_4	C16H11Cl2F3O3	H	379.00	379.01	7.5x10^6	N
D2S3	Rh_2piv_4	C24H18Cl3F3O2	-H	499.02	498.86	7.5X10^7	Y
D2S4	Rh_2piv_4	C17H17ClF3NO4	H	392.08	392.02	3x10^7	Y
D2S5	Rh_2piv_4	C20H21ClF3NO3	-H	414.11	414.11	6x10^7	Y
D2S6	Rh_2piv_4	C19H14ClF3O3	Na	405.04	404.80	1x10^7	Y
D2S7	Rh_2piv_4	C21H19ClF3NO2	H	409.11	410.02	7.5x10^8	Y
D2S8	Rh_2piv_4	C19H15Cl2F3O3	Na	441.02	440.97	6x10^6	Y
D2S9	Rh_2piv_4	C19H13Cl2F3O2	H	401.02	400.93	2x10^6	N
D2S10	Rh_2piv_4	C14H14ClF3O4S	-H	369.01	368.85	2x10^7	Y
D2blank	Rh_2piv_4	C20H12Cl2F6O4	-H	498.99	498.99	1x10^6	N
D2S1	Rh_2pfb_4	C18H12Cl2F3NO2	-H	400.01	399.92	1x10^8	Y
D2S2	Rh_2pfb_4	C16H11Cl2F3O3	Na	400.99	400.89	1.5x10^6	N
D2S3	Rh_2pfb_4	C24H18Cl3F3O2	-	500.03	-	-	N
D2S4	Rh_2pfb_4	C17H17ClF3NO4	H	392.08	392.09	5x10^7	Y
D2S5	Rh_2pfb_4	C20H21ClF3NO3	-	415.12	-	-	N
D2S6	Rh_2pfb_4	C19H14ClF3O3	-	382.06	-	-	N
D2S7	Rh_2pfb_4	C21H19ClF3NO2	H	410.11	410.02	7.5x10^8	Y
D2S8	Rh_2pfb_4	C19H15Cl2F3O3	Na	441.02	440.96	2x10^6	Y

D2S9	Rh_2pfb_4	C19H13Cl2F3O2	H	401.02	400.92	1x10^7	Y
D2S10	Rh_2pfb_4	C14H14ClF3O4S	-	370.03	-	-	N
D2blank	Rh_2pfb_4	C20H12Cl2F6O4	-	500.00	-	-	N
D3S1	Rh_2piv_4	C23H17Cl3N2O	H	445.04	444.93	2.5X10^7	Y
D3S2	Rh_2piv_4	C21H16Cl3NO2	H	422.02	421.90	2x10^8	Y
D3S3	Rh_2piv_4	C29H23Cl4NO	-	541.05	-	-	N
D3S4	Rh_2piv_4	C22H22Cl2N2O3	H	433.10	432.98	2x10^8	Y
D3S5	Rh_2piv_4	C25H26Cl2N2O2	K	495.10	495.07	1.25x10^7	N
D3S6	Rh_2piv_4	C24H19Cl2NO2	-	423.08	-	-	N
D3S7	Rh_2piv_4	C26H24Cl2N2O	H	451.13	451.13	2x10^7	N
D3S8	Rh_2piv_4	C24H20Cl3NO2	-	459.06	-	-	N
D3S9	Rh_2piv_4	C24H18Cl3NO	-	441.05	-	-	N
D3S10	Rh_2piv_4	C19H19Cl2NO3S	-H	410.05	410.04	2x10^5	N
D3blank	Rh_2piv_4	C15H11Cl2NO	-H	290.01	289.71	5x10^6	Y
D3S1	Rh_2pfb_4	C23H17Cl3N2O	H	445.04	444.95	7.5x10^7	Y
D3S2	Rh_2pfb_4	C21H16Cl3NO2	-	419.02	-	-	N
D3S3	Rh_2pfb_4	C29H23Cl4NO	-	541.05	-	-	N
D3S4	Rh_2pfb_4	C22H22Cl2N2O3	H	433.10	432.97	4x10^8	Y
D3S5	Rh_2pfb_4	C25H26Cl2N2O2	-	456.14	-	-	N
D3S6	Rh_2pfb_4	C24H19Cl2NO2	-	423.08	-	-	N
D3S7	Rh_2pfb_4	C26H24Cl2N2O	-	450.13	-	-	N
D3S8	Rh_2pfb_4	C24H20Cl3NO2	-	459.06	-	-	N
D3S9	Rh_2pfb_4	C24H18Cl3NO	-	441.05	-	-	N
D3S10	Rh_2pfb_4	C19H19Cl2NO3S	-	411.05	-	-	N
D3blank	Rh_2pfb_4	C15H11Cl2NO	H	292.02	292.02	4x10^7	Y
D3S1	Rh_2piv_4	C20H19ClN2O	H	339.12	339.02	1x10^8	Y
D3S2	Rh_2piv_4	C18H18ClNO2	H	316.10	315.96	1.5x10^8	Y
D3S3	Rh_2piv_4	C26H25Cl2NO	-	437.13	-	-	N
D4S4	Rh_2piv_4	C19H24N2O3	NH_4	347.18	347.08	1x10^7	Y
D4S5	Rh_2piv_4	C22H28N2O2	-	352.2151	-	-	N
D4S6	Rh_2piv_4	C21H21NO2	H	320.16	320.01	7x10^7	Y
D4S7	Rh_2piv_4	C23H26N2O	-	346.21	-	-	N
D4S8	Rh_2piv_4	C21H22ClNO2	2M + Na	733.26	733.19	2x10^8	Y
D4S9	Rh_2piv_4	C21H20ClNO	H	338.12	338.03	1.7x10^8	Y
D4S10	Rh_2piv_4	C16H21NO3S	H	308.12	307.97	2x10^7	Y
D4blank	Rh_2piv_4	C24H26N2O2	H	375.20	375.1	3x10^8	Y
D4S1	Rh_2pfb_4	C20H19ClN2O	H	339.12	339.01	1x10^8	Y
D4S2	Rh_2pfb_4	C18H18ClNO2	-	315.10	-	-	N
D4S3	Rh_2pfb_4	C26H25Cl2NO	Na	460.12	460.23	1x10^7	Y
D4S4	Rh_2pfb_4	C19H24N2O3	H	329.18	329.19	3x10^7	N
D4S5	Rh_2pfb_4	C22H28N2O2	-H	351.22	351.21	5x10^5	N
D4S6	Rh_2pfb_4	C21H21NO2	H	320.16	320.05	8x10^6	Y
D4S7	Rh_2pfb_4	C23H26N2O	-	346.20	-	-	N
D4S8	Rh_2pfb_4	C21H22ClNO2	2M + Na	733.26	733.21	2x10^7	N

D4S9	Rh_2pfb_4	C21H20ClNO	H	338.12	338.04	4x10^7	Y
D4S10	Rh_2pfb_4	C16H21NO3S	H	308.12	308.13	1x10^7	N
D4blank	Rh_2pfb_4	C24H26N2O2	H	375.20	375.08	3x10^8	Y
D5S1	Rh_2piv_4	C16H19ClN2O2	H	307.11	306.94	1x10^8	Y
D5S2	Rh_2piv_4	C14H18ClNO3	H	284.10	283.88	3x10^7	Y
D5S3	Rh_2piv_4	C22H25Cl2NO2	H	406.13	406.13	5x10^6	N
D5S4	Rh_2piv_4	C15H24N2O4	H	297.17	297.18	4x10^6	N
D5S5	Rh_2piv_4	C18H28N2O3	NH_4	338.24	338.23	1x10^7	N
D5S6	Rh_2piv_4	C17H21NO3	H	288.15	287.94	1x10^7	Y
D5S7	Rh_2piv_4	C19H26N2O2	-	314.30	-	-	N
D5S8	Rh_2piv_4	C17H22ClNO3	2M + Na	669.25	669.24	1x10^8	Y
D5S9	Rh_2piv_4	C17H20ClNO2	H	306.12	305.94	3x10^7	Y
D5S10	Rh_2piv_4	C12H21NO4S	-	275.12	-	-	N
D5blank	Rh_2piv_4	C8H13NO2	-	155.09	-	-	N
D5S1	Rh_2pfb_4	C16H19ClN2O2	H	307.11	306.92	3x10^7	Y
D5S2	Rh_2pfb_4	C14H18ClNO3	H	284.10	283.86	2x10^7	Y
D5S3	Rh_2pfb_4	C22H25Cl2NO2	Na	428.12	428.22	2x10^6	Y
D5S4	Rh_2pfb_4	C15H24N2O4	H	297.17	297.18	5x10^6	N
D5S5	Rh_2pfb_4	C18H28N2O3	-	320.21	-	-	N
D5S6	Rh_2pfb_4	C17H21NO3	H	288.15	288.16	5x10^6	N
D5S7	Rh_2pfb_4	C19H26N2O2	H	315.20	315.21	1x10^7	N
D5S8	Rh_2pfb_4	C17H22ClNO3	2M + Na	669.25	669.24	5x10^7	Y
D5S9	Rh_2pfb_4	C17H20ClNO2	H	306.12	305.93	2.5x10^6	Y
D5S10	Rh_2pfb_4	C12H21NO4S	-	275.12	-	-	N
D5blank	Rh_2pfb_4	C8H13NO2	2M + H	311.20	311.04	1.5X10^7	N
D6S1	Rh_2piv_4	C17H14ClNO2	-H	298.07	298.06	1x10^6	N
D6S2	Rh_2piv_4	C15H13ClO3	H	277.06	277.06	3x10^6	N
D6S3	Rh_2piv_4	C23H20Cl2O2	-	398.08	-	-	N
D6S4	Rh_2piv_4	C16H19NO4	H	290.13	290.14	5x10^6	N
D6S5	Rh_2piv_4	C19H24NO3	K	353.14	353.05	2x10^7	Y
D6S6	Rh_2piv_4	C18H16O3	H	281.11	281.12	7.5x10^6	N
D6S7	Rh_2piv_4	C20H22NO2	H	309.17	309.17	1.5x10^8	Y
D6S8	Rh_2piv_4	C18H17ClO3	H	317.09	317.06	3x10^7	Y
D6S9	Rh_2piv_4	C18H15ClO2	H	299.08	299.08	7.5x10^6	N
D6S10	Rh_2piv_4	C13H16O4S	H	269.08	269.08	3x10^7	N
D6blank	Rh_2piv_4	C18H16O4	H	297.11	297.11	7.5x10^7	Y
D6S1	Rh_2pfb_4	C17H14ClNO2	H	300.07	299.94	1x10^7	Y
D6S2	Rh_2pfb_4	C15H13ClO3	-	276.06	-	-	N
D6S3	Rh_2pfb_4	C23H20Cl2O2	-	398.08	-	-	N
D6S4	Rh_2pfb_4	C16H19NO4	H	290.13	290.14	5x10^6	N
D6S5	Rh_2pfb_4	C19H24NO3	K	353.14	353.11	1x10^7	N
D6S6	Rh_2pfb_4	C18H16O3	-	280.11	-	-	N
D6S7	Rh_2pfb_4	C20H22NO2	H	309.17	309.17	1.5x10^8	Y
D6S8	Rh_2pfb_4	C18H17ClO3	2M + Na	655.16	655.05	6x10^7	Y

D6S9	Rh_2pfb_4	C18H15ClO2	H	299.08	299.08	4x10^7	N
D6S10	Rh_2pfb_4	C13H16O4S	H	269.08	269.00	3x10^7	Y
D6blank	Rh_2pfb_4	C18H16O4	-	296.10	-	-	N
D7S1	Rh_2piv_4	C14H12ClN3O2	H	290.06	289.93	2x10^7	Y
D7S2	Rh_2piv_4	C12H11ClN2O3	H	267.05	266.89	6x10^6	Y
D7S3	Rh_2piv_4	C20H18Cl2N2O2	H	389.08	389.08	6x10^6	Y
D7S4	Rh_2piv_4	C13H17N3O4	Na	302.11	302.01	2x10^6	Y
D7S5	Rh_2piv_4	C16H21N3O3	-H	302.16	302.01	1x10^6	Y
D7S6	Rh_2piv_4	C15H14N2O3	H	271.10	270.93	3x10^7	Y
D7S7	Rh_2piv_4	C17H20N3O2	NH_4	316.19	316.10	2x10^6	N
D7S8	Rh_2piv_4	C15H15ClN2O3	H	307.08	306.96	5x10^7	Y
D7S9	Rh_2piv_4	C15H13ClN2O2	H	289.07	288.93	4x10^7	Y
D7S10	Rh_2piv_4	C10H14N2O4S	H	259.07	258.87	6x10^6	Y
D7blank	Rh_2piv_4	C12H12N4O4	H	276.09	276.95	8x10^6	Y
D7S1	Rh_2pfb_4	C14H12ClN3O2	H	290.06	289.92	1.5x10^7	Y
D7S2	Rh_2pfb_4	C12H11ClN2O3	H	267.05	266.89	1x10^7	Y
D7S3	Rh_2pfb_4	C20H18Cl2N2O2	H	389.07	389.06	1.5x10^7	Y
D7S4	Rh_2pfb_4	C13H17N3O4	-H	278.12	278.11	4x10^6	N
D7S5	Rh_2pfb_4	C16H21N3O3	H	304.16	304.17	4x10^6	N
D7S6	Rh_2pfb_4	C15H14N2O3	H	271.10	270.91	1x10^7	Y
D7S7	Rh_2pfb_4	C17H19N3O2	H	298.15	298.06	1.5x10^7	Y
D7S8	Rh_2pfb_4	C15H15ClN2O3	2M + Na	635.14	635.04	1x10^7	Y
D7S9	Rh_2pfb_4	C15H13ClN2O2	H	289.07	288.92	1.5x10^7	Y
D7S10	Rh_2pfb_4	C10H14N2O4S	H	259.07	258.91	3x10^6	Y
D7blank	Rh_2pfb_4	C12H12N4O4	H	277.09	276.95	7x10^7	Y

8. Docking of ADS Ligands and similarity analysis

8.1 Molecular docking

Docking of compounds into the binding site (PDB IDs: 6Q9H and 4HG7) was performed using MOE (Molecular Operating Environment)[7] from the Chemical Computing Group (Montreal, Canada) (Figure S5). Novel molecules were docked as database and 10 conformations were generated for each molecule using the Amber10:EHT forcefield, using an aromatic pharmacophore in Leu26 and Trp23 hot spots. Among these, the conformations with the lowest docking scores were chosen to study the likely binding orientations of the ligands and each complex was assessed and ranked by the London ΔG energy scoring function.

P2

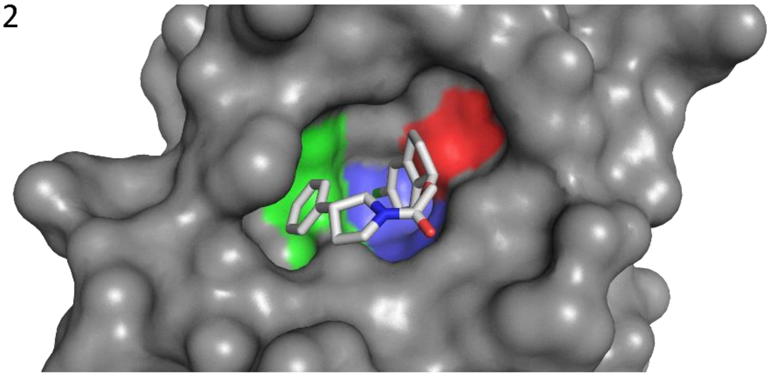

P4

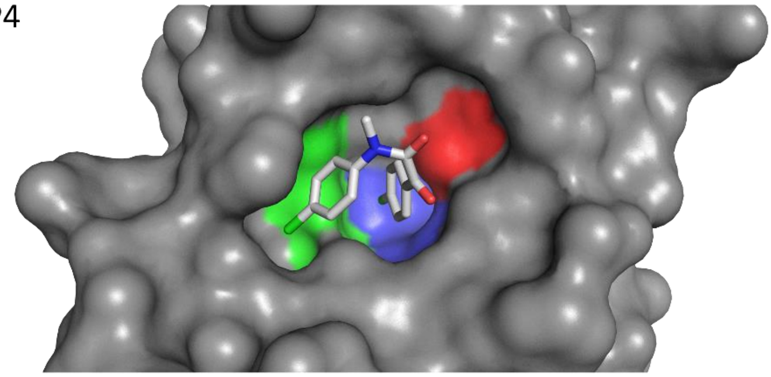

P5

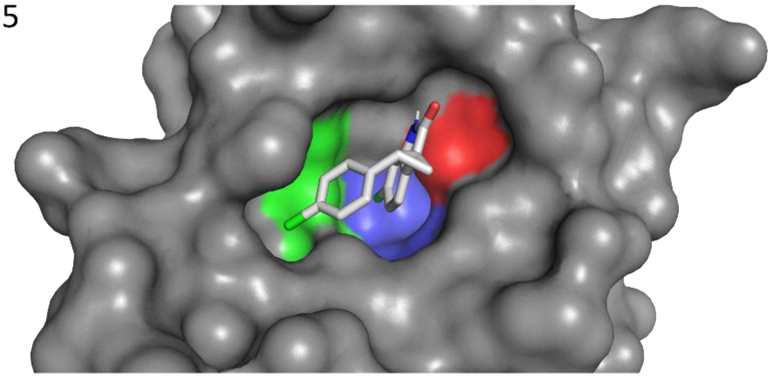

P6

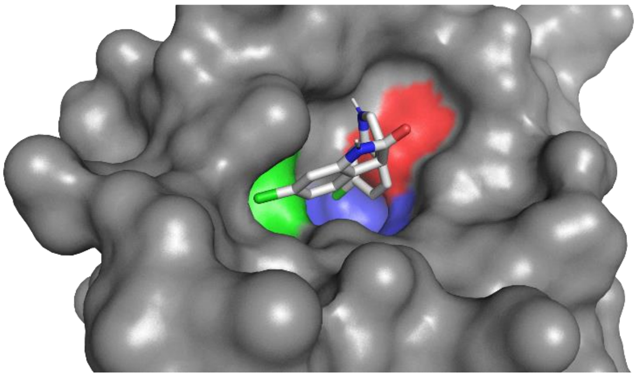

Figure **S5**. **P2**, **P4**, **P5** and **P6** docked into the binding site (PDB: 6Q9H); the subpockets targeted by p53 hotspot residues F19 (red), W23 (blue) and L26 (green) are shown.

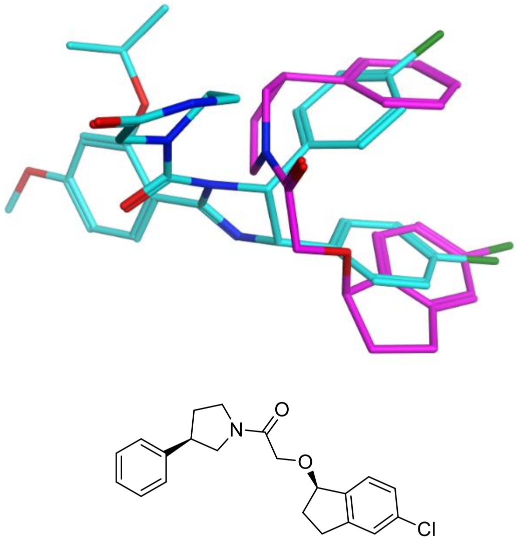

(*R*,*R*)-**P2**

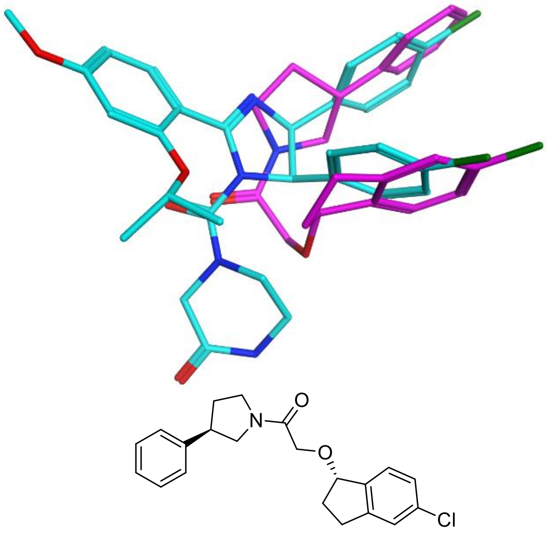

(*S*,*R*)-**P2**

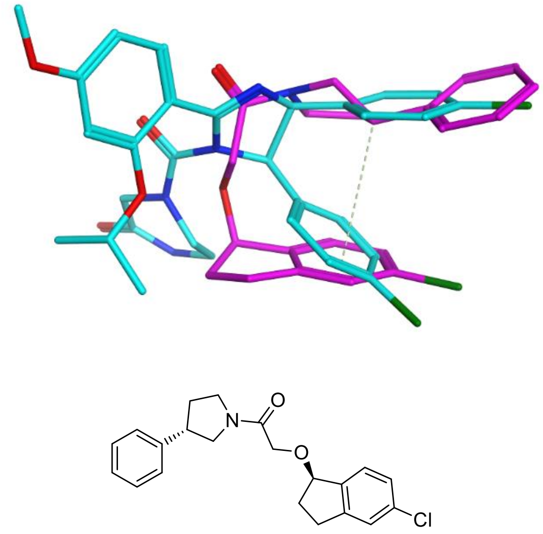

(*R*,*S*)-**P2**

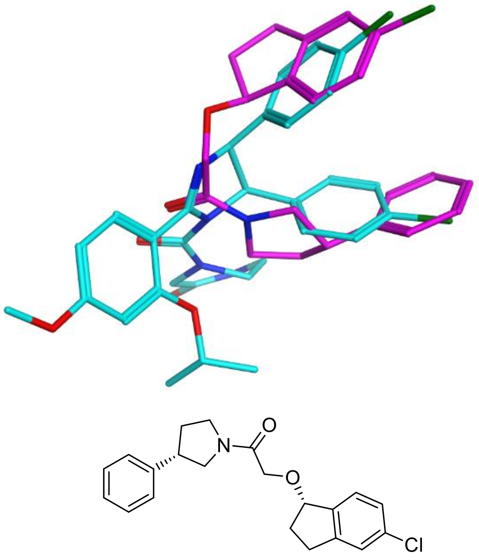

(*S*,*S*)-**P2**

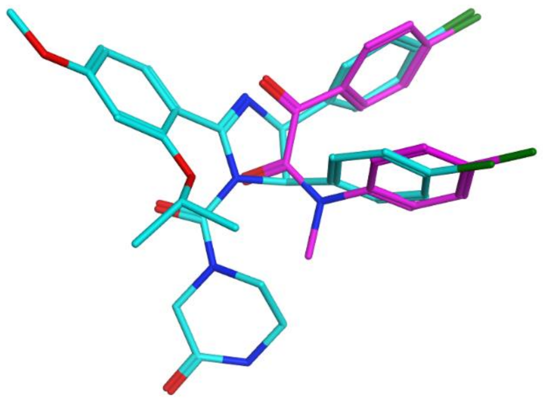

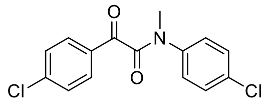

P4

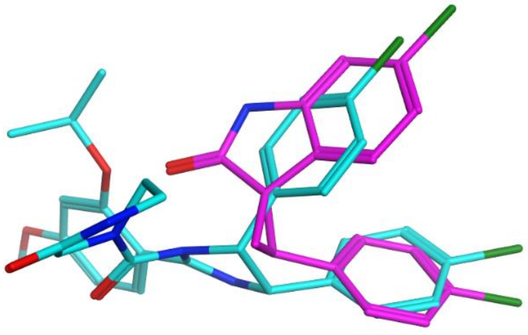

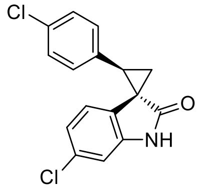

(1*S*,2*R*)-**P5**

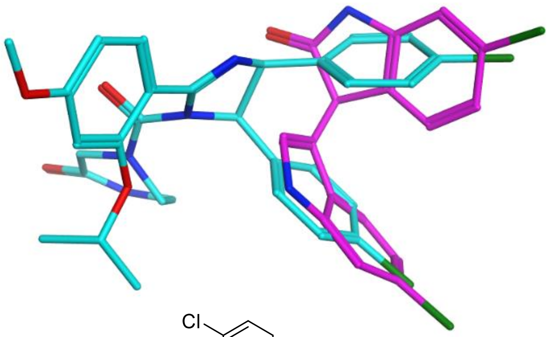

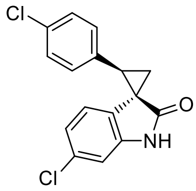

(1*R*,2*R*)-**P5**

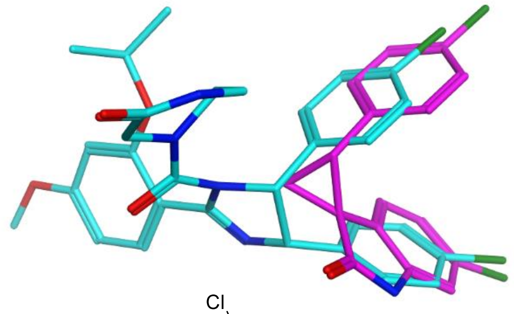

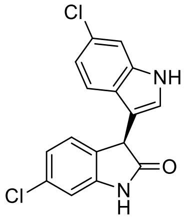

(*R*)-**P6**

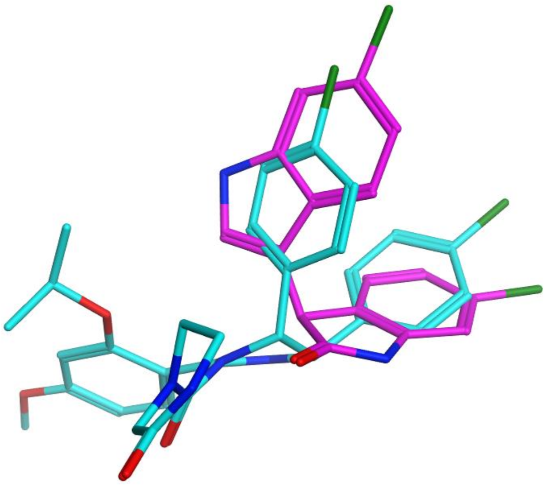

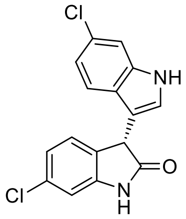

(*S*)-**P6**

Figure **S6**. Overlay of docked poses of MDM2 binders and Nutlin-3a (PDB ID: 4HG7)

8.2 X-ray structures of AM-8735 and MI-77301

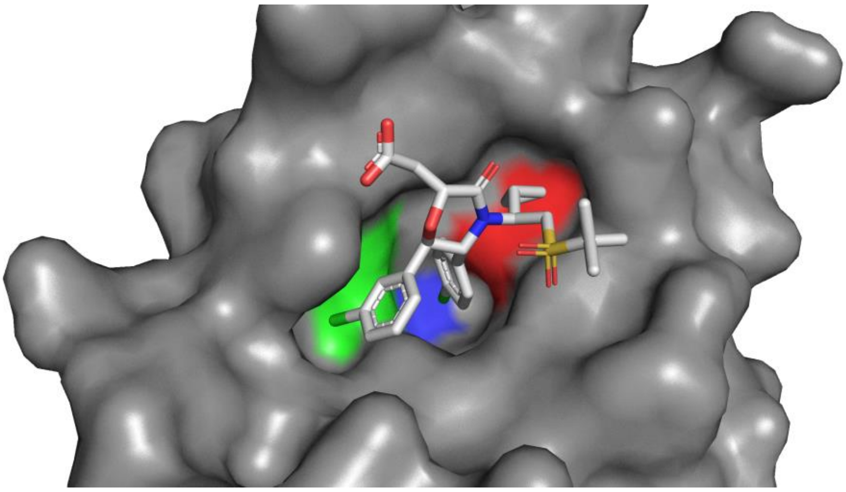

Figure **S7**. X-ray crystal structure of AM-8735 bound to *h*DM2 (PDB: 4OBA); the subpockets targeted by p53 hotspot residues F19 (red), W23 (blue) and L26 (green) are shown.[8]

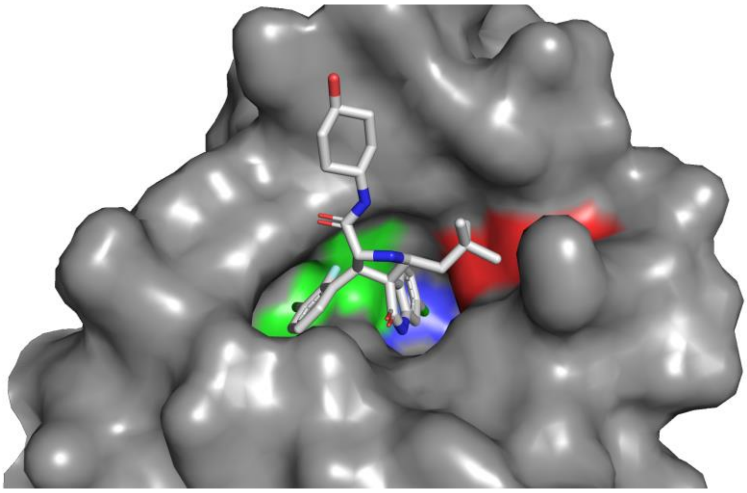

Figure **S8**. X-ray crystal structure of MI-77301 bound to *h*DM2 (PDB: 5TRF); the subpockets targeted by p53 hotspot residues F19 (red), W23 (blue) and L26 (green) are shown.[9]

8.3 Similarity analysis

1769 compounds with annotated activity towards *h*DM2 (referred to as MDM2 within ChEMBL) were obtained from the ChEMBL database (accessed: 16/01/2020). Subsequent processing removed duplicate molecules leaving 1314 compounds (see accompanying Excel spreadsheet) which were then used for further analysis. The Morgan molecular fingerprint was then computed for each molecule using RDKit[10] and the pairwise Tanimoto similarity scores calculated.

Table **S3**. Tanimoto similarity analysis comparing ADS products **P1**-**P6** to 1314 known hDM2 ligands from the ChEMBL database.

Metric	**P1**	**P2**	**P3**	**P4**	**P5**	**P6**
Mean	0.37	0.34	0.36	0.26	0.43	0.44
Median	0.38	0.34	0.36	0.25	0.44	0.45
Minimum similarity	0.12	0.12	0.13	0.13	0.13	0.12
Maximum similarity	0.50	0.48	0.46	0.37	0.61	0.51

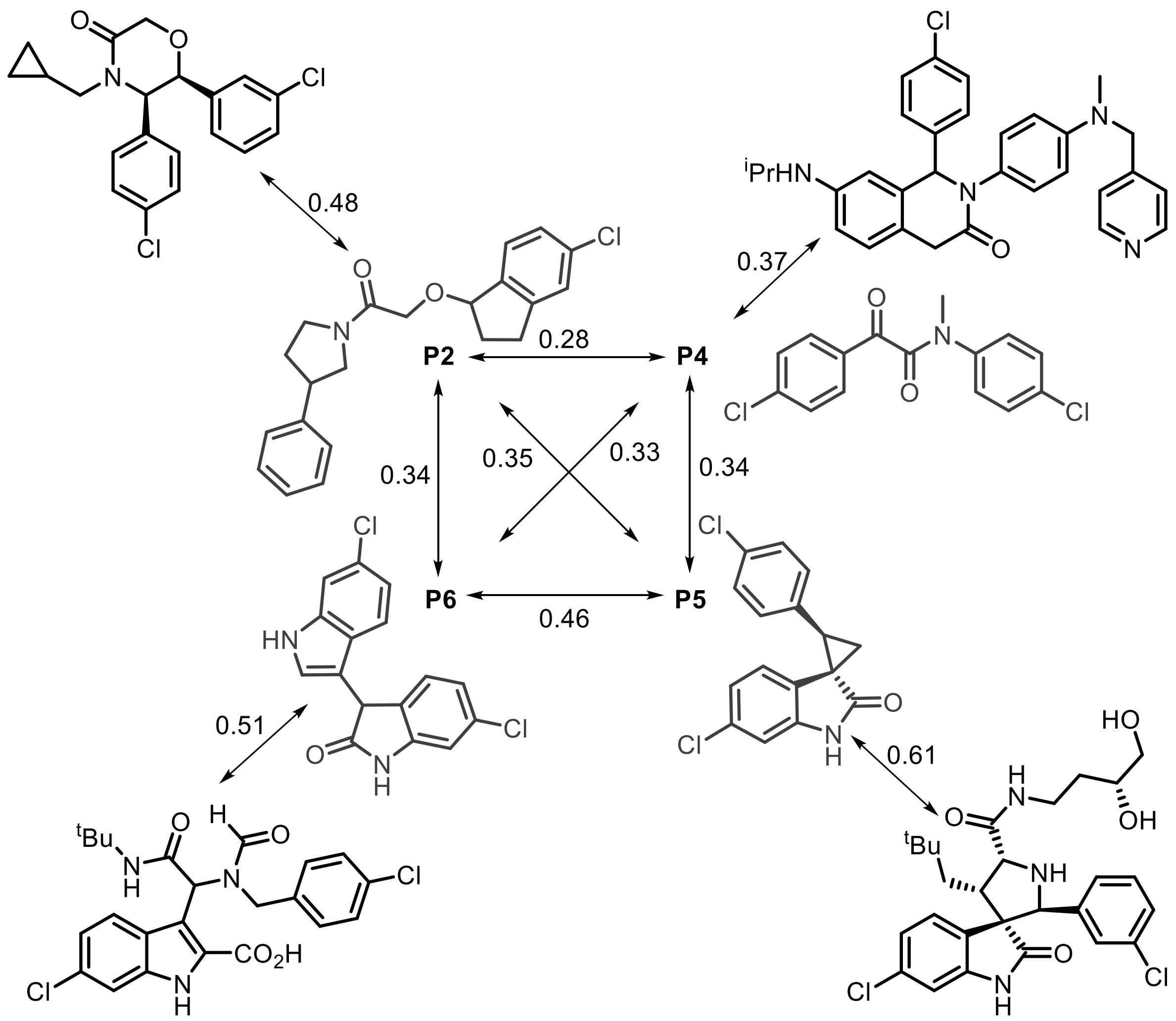

Figure S9. Molecular similarities of the p53/*h*DM2 PPI inhibitors **P2**, **P4**, **P5** and **P6** and their nearest neighbour *h*DM2 ligands in ChEMBL.

9. Spectra

D9

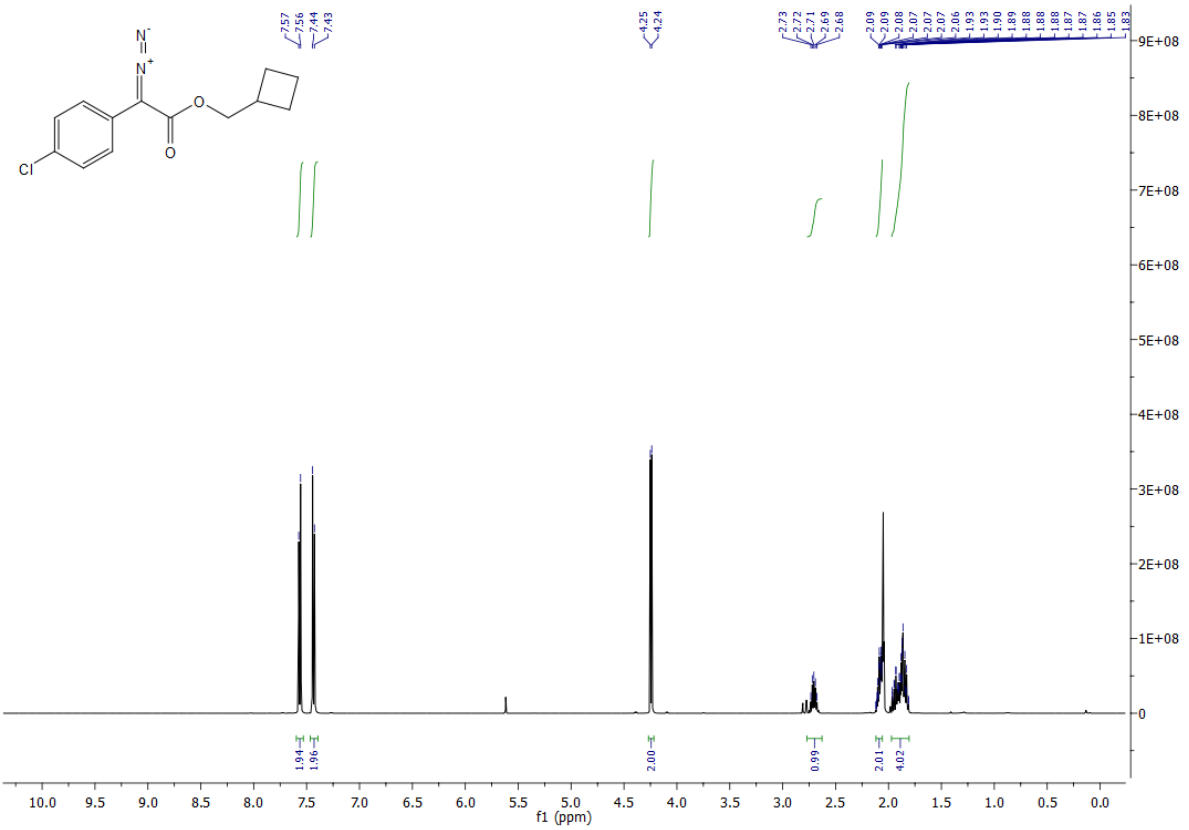

7.57
7.56
7.44
7.43
4.25
4.24
2.73
2.72
2.71
2.69
2.68
1.94
1.96
2.00
0.99
2.01
4.02
f1 (ppm)

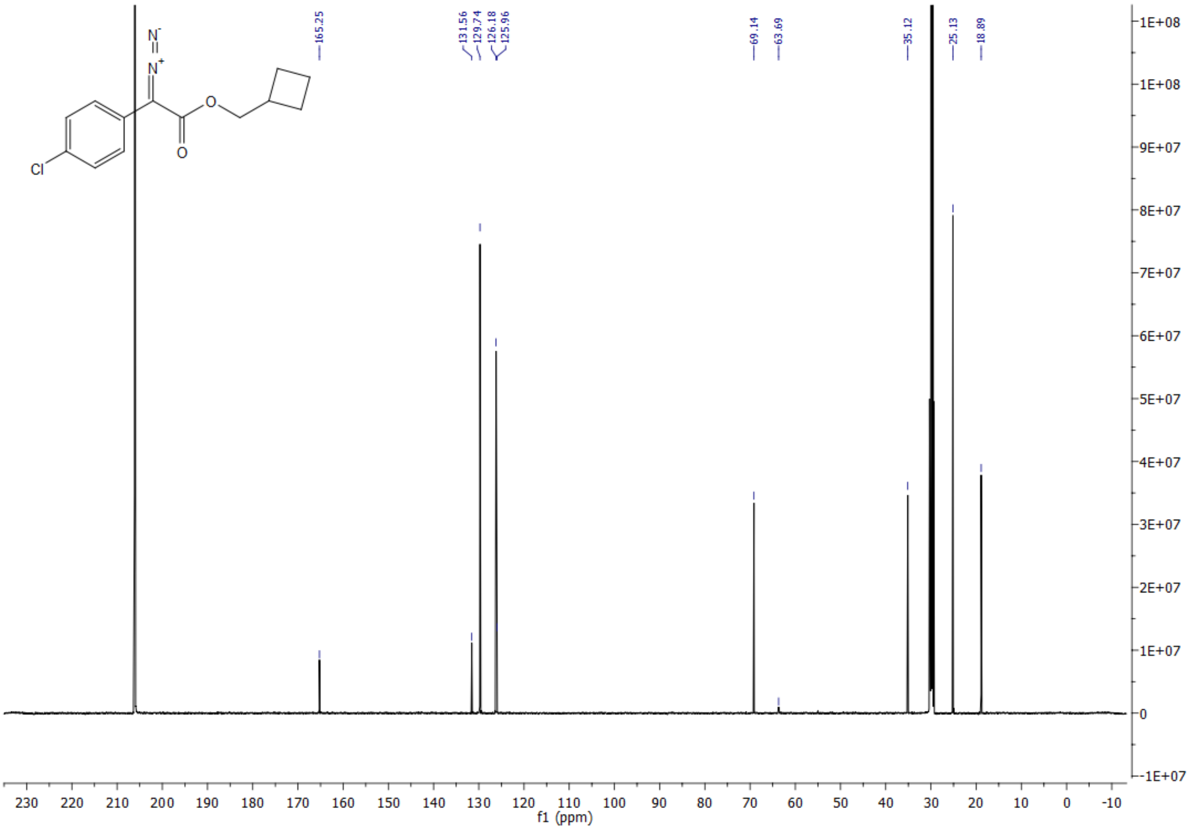

165.25
131.56
129.74
126.18
125.96
69.14
63.69
35.12
25.13
18.89
f1 (ppm)

D10

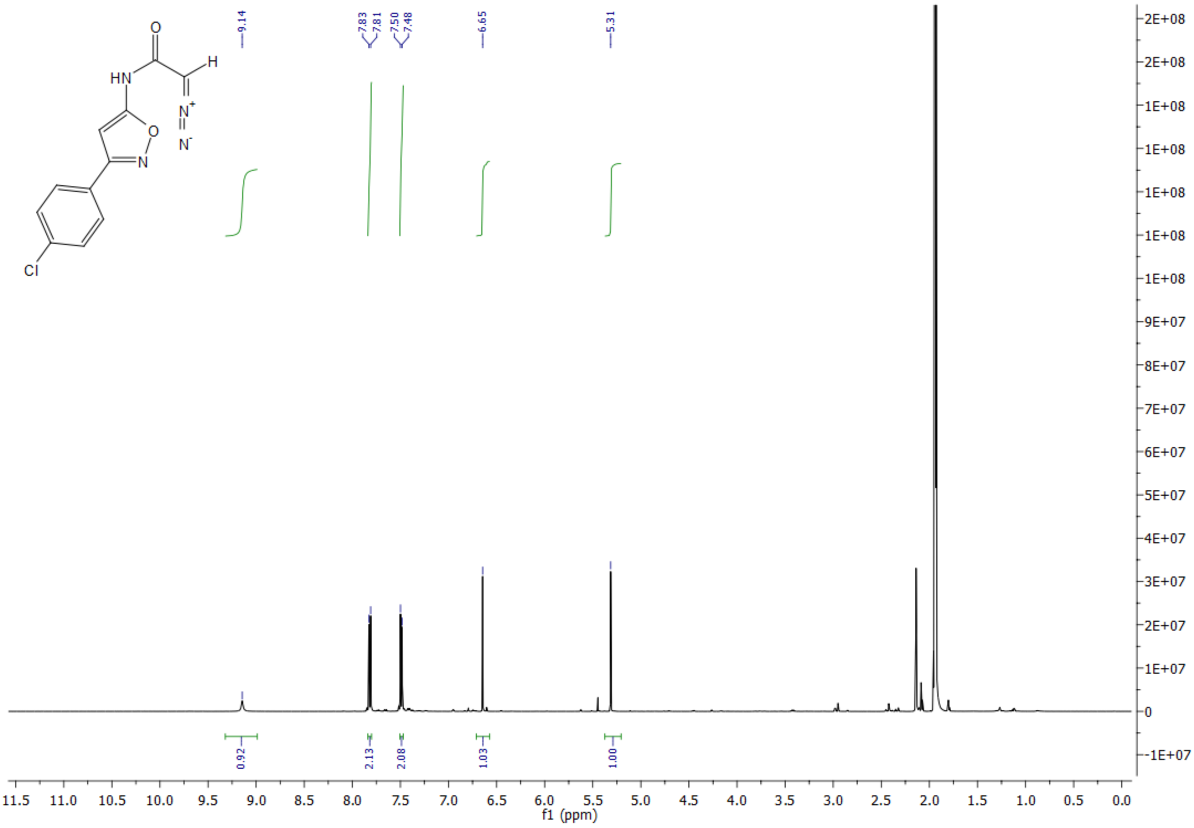

9.14
7.83
7.81
7.50
7.48
6.65
5.31
0.92
2.13
2.08
1.03
1.00
f1 (ppm)
HN
O
H
N+
N-
O
N
Cl

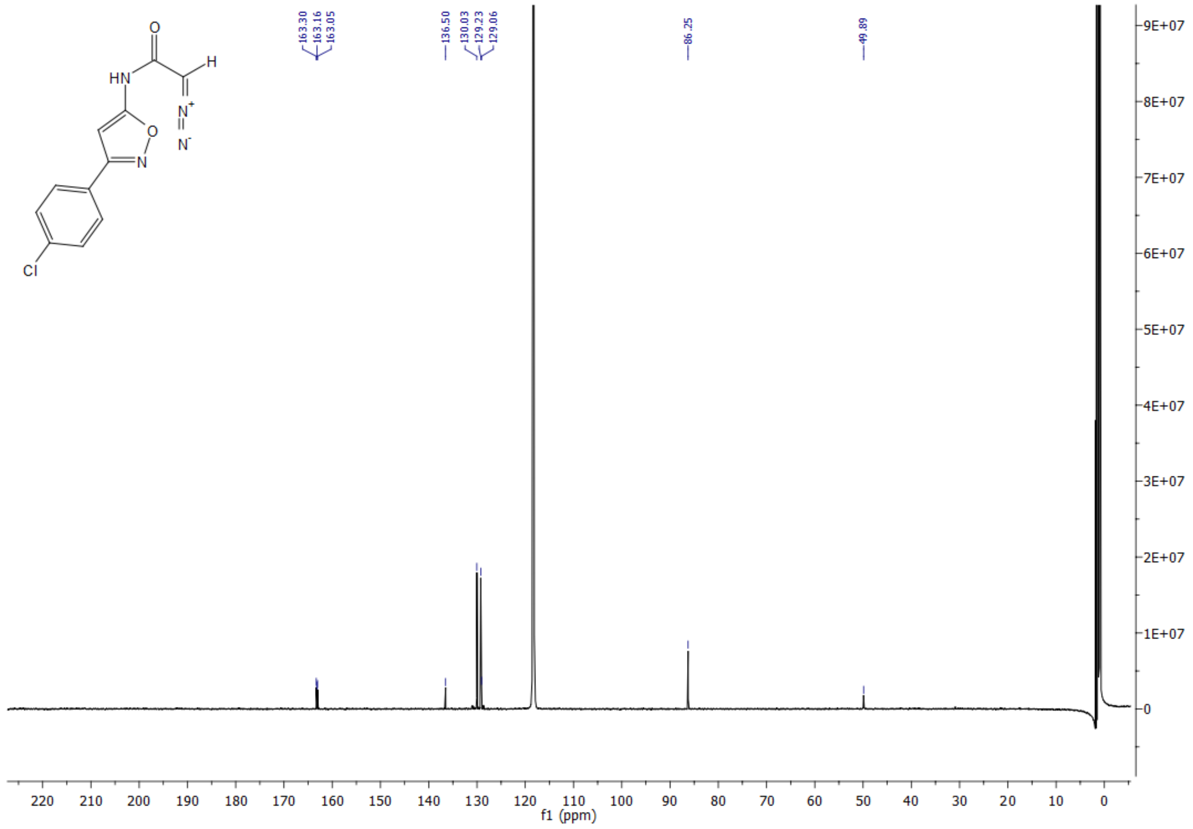

163.30
163.16
163.05
136.50
130.03
129.23
129.06
86.25
49.89
f1 (ppm)
HN
O
H
N+
N-
O
N
Cl

S5

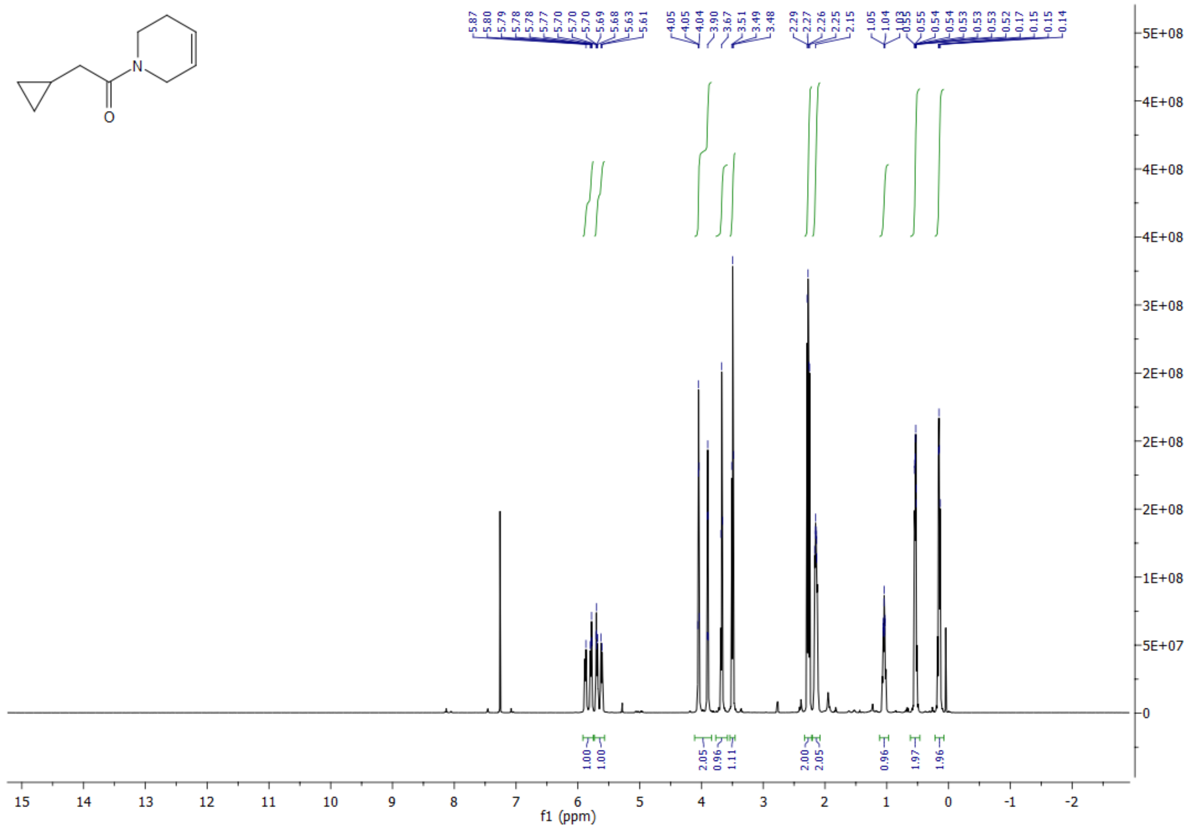
N
O
5.87
5.80
5.79
5.78
5.78
5.77
5.77
5.70
5.70
5.70
5.69
5.68
5.63
5.61
4.05
4.05
4.04
3.90
3.67
3.51
3.49
3.48
2.29
2.27
2.26
2.25
2.15
1.05
1.04
1.03
0.55
0.55
0.54
0.54
0.53
0.53
0.53
0.52
0.17
0.15
0.15
0.14
1.00
1.00
2.05
0.96
1.11
2.00
2.05
0.96
1.97
1.96
f1 (ppm)

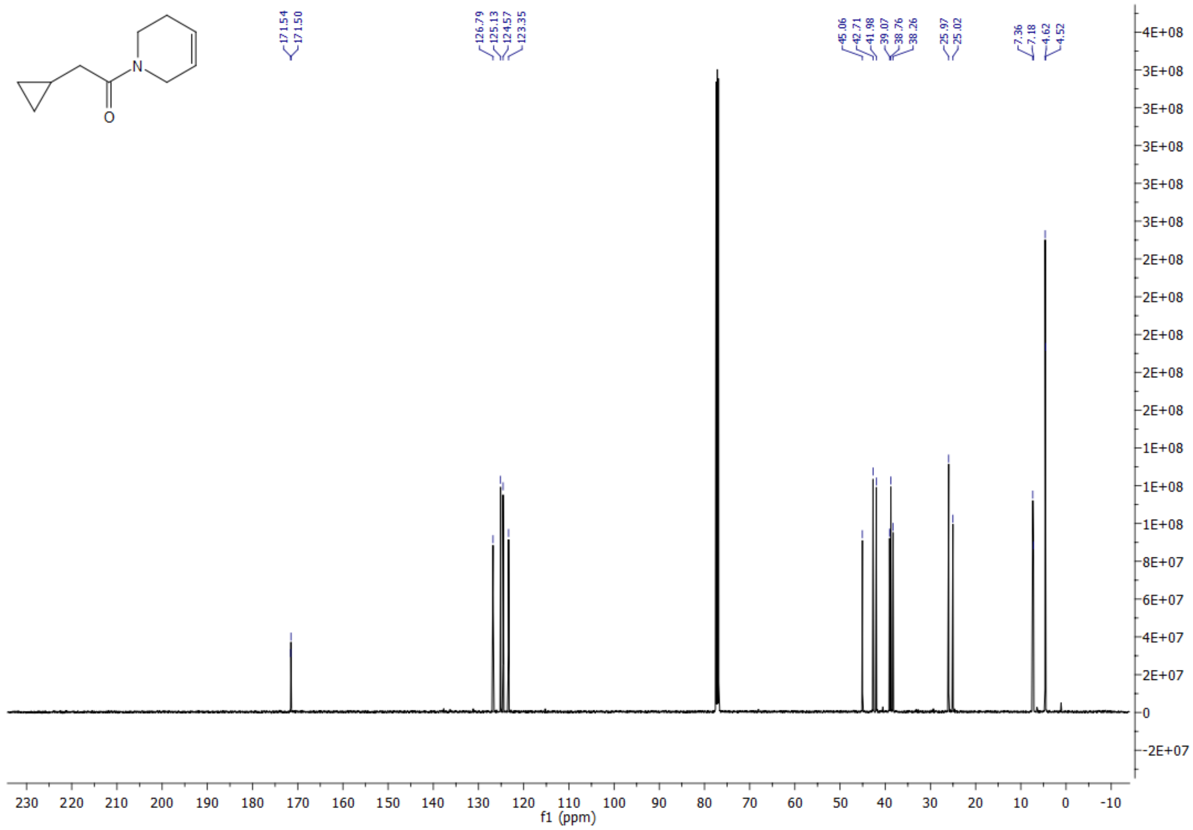
N
O
171.54
171.50
126.79
125.13
124.57
123.35
45.06
42.71
41.98
39.07
38.76
38.26
25.97
25.02
7.36
7.18
4.62
4.52
f1 (ppm)

P2

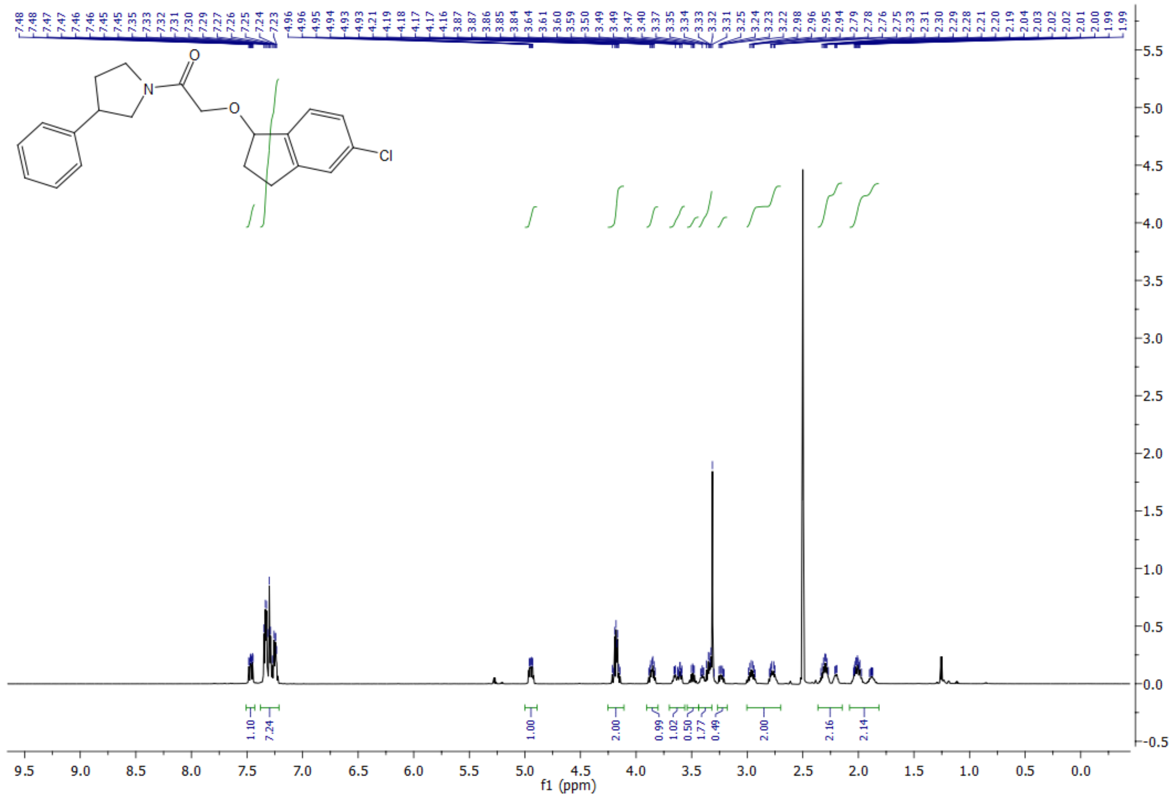

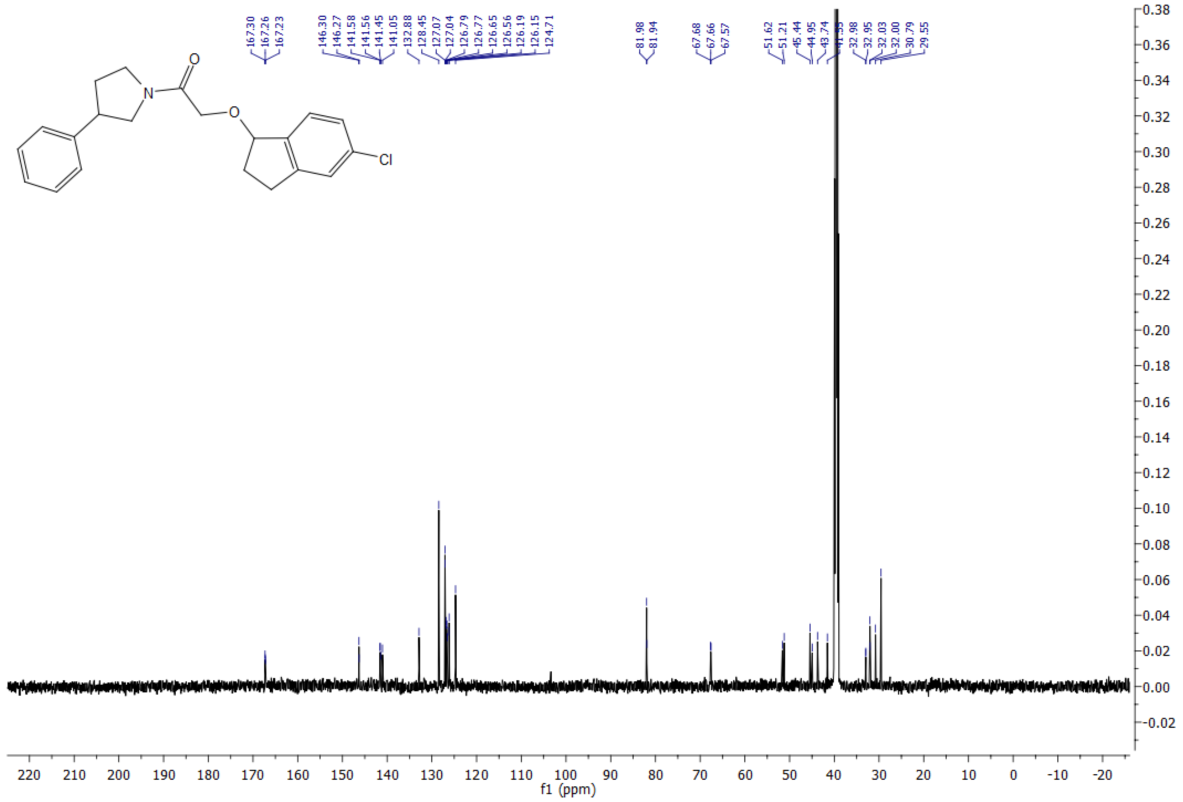

P2a

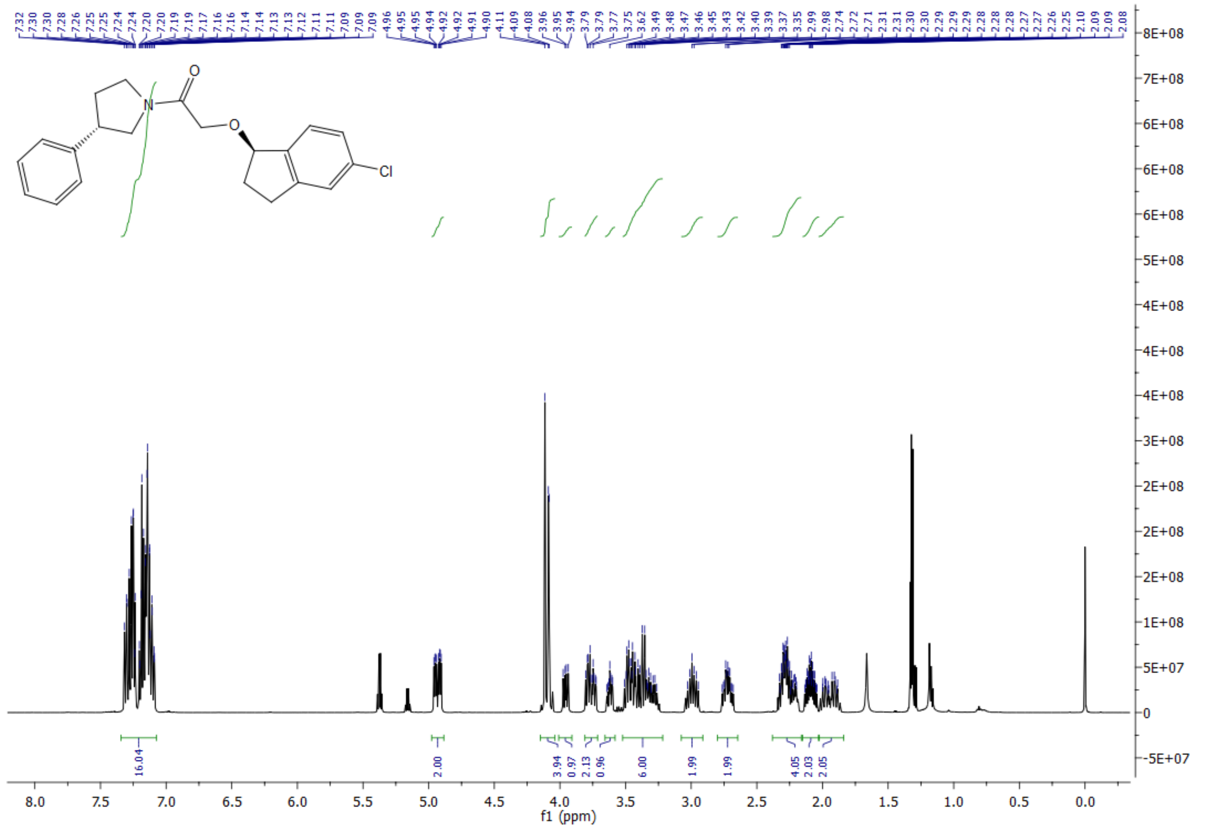
7.32 7.30 7.30 7.28 7.26 7.25 7.25 7.24 7.24 7.20 7.20 7.19 7.19 7.17 7.16 7.16 7.14 7.14 7.13 7.13 7.12 7.11 7.11 7.09 7.09 7.09 4.96 4.95 4.95 4.94 4.92 4.92 4.91 4.90 4.11 4.09 4.08 3.96 3.95 3.94 3.79 3.79 3.77 3.75 3.62 3.49 3.48 3.47 3.46 3.45 3.43 3.42 3.40 3.39 3.37 3.35 2.99 2.98 2.74 2.72 2.71 2.31 2.31 2.30 2.30 2.29 2.29 2.29 2.28 2.28 2.28 2.27 2.27 2.26 2.25 2.10 2.09 2.09 2.08
N
O
O
Cl
16.04
2.00
3.94
0.97
2.13
0.96
6.00
1.99
1.99
4.05
2.03
2.05
f1 (ppm)

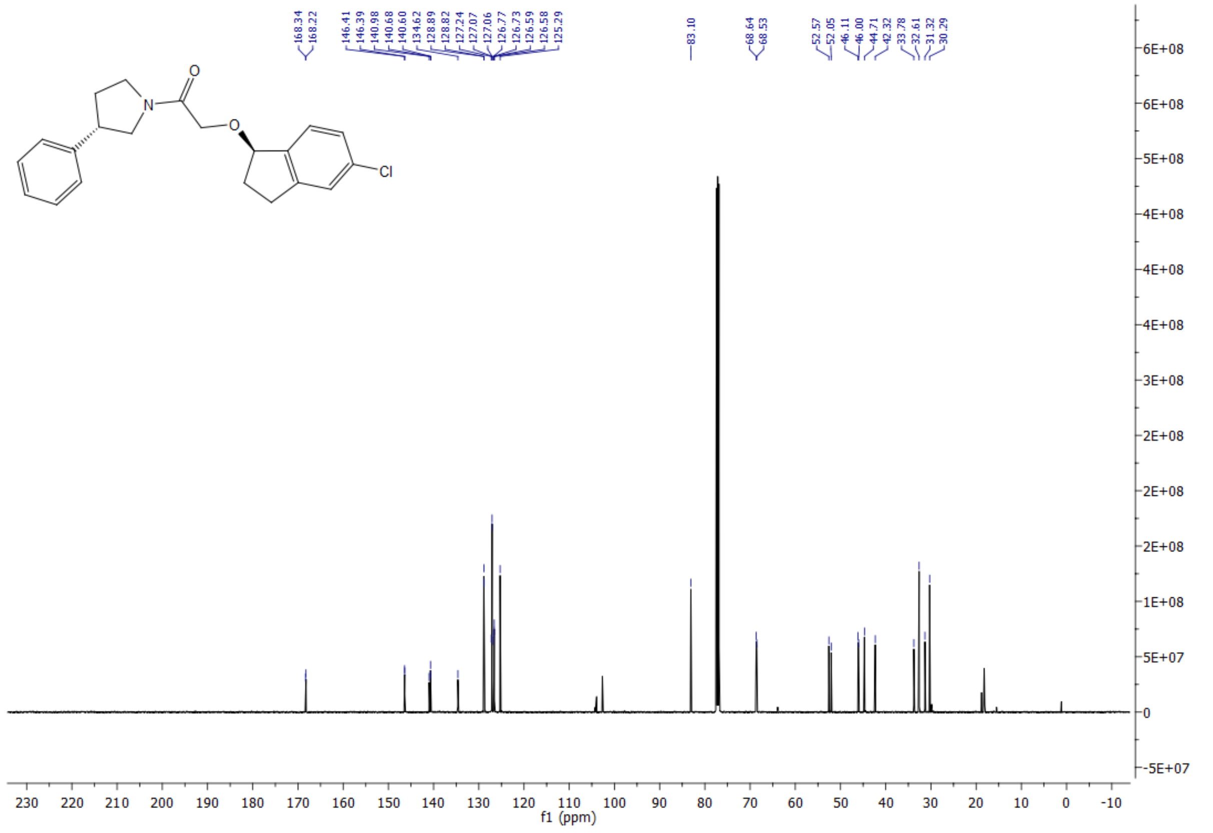
168.34 168.22 146.41 146.39 140.98 140.68 140.60 134.62 128.89 128.82 127.24 127.07 127.06 126.77 126.73 126.59 126.58 125.29 83.10 68.64 68.53 52.57 52.05 46.11 46.00 44.71 42.32 33.78 32.61 31.32 30.29
N
O
O
Cl
f1 (ppm)

ent-**P2a**

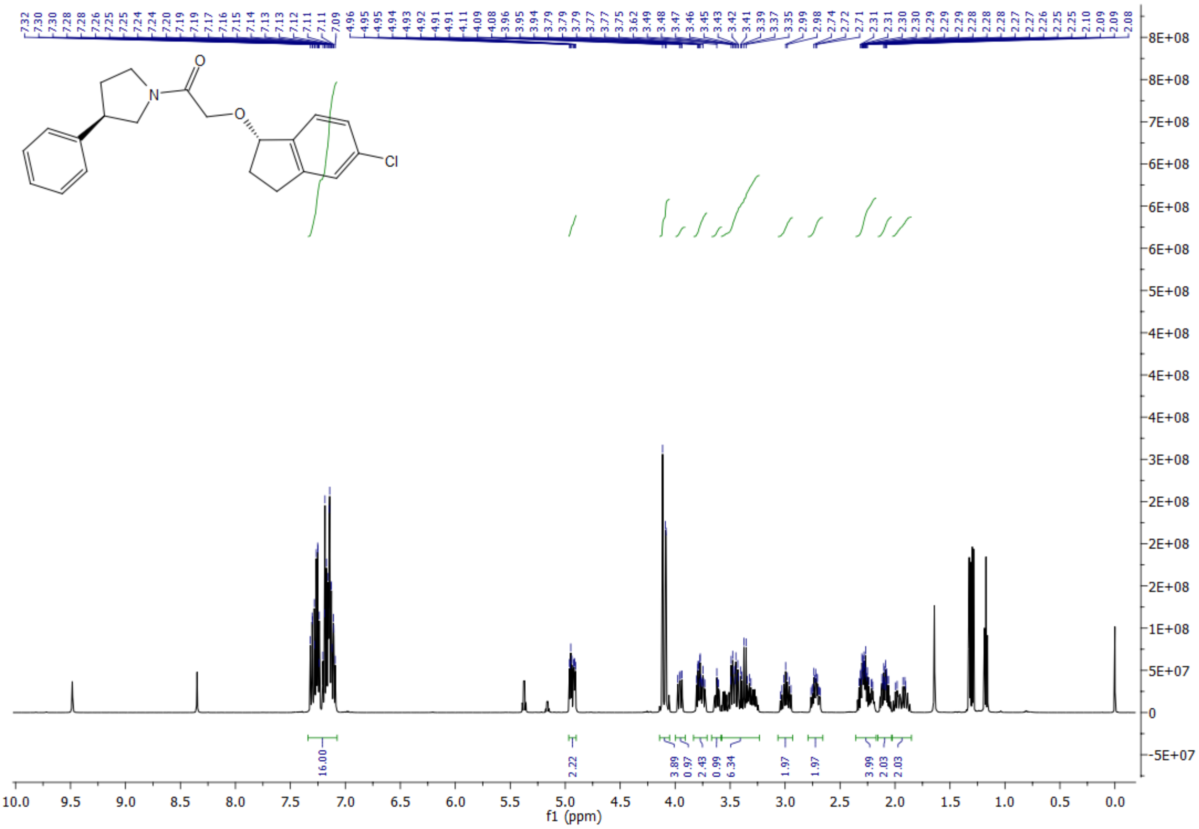

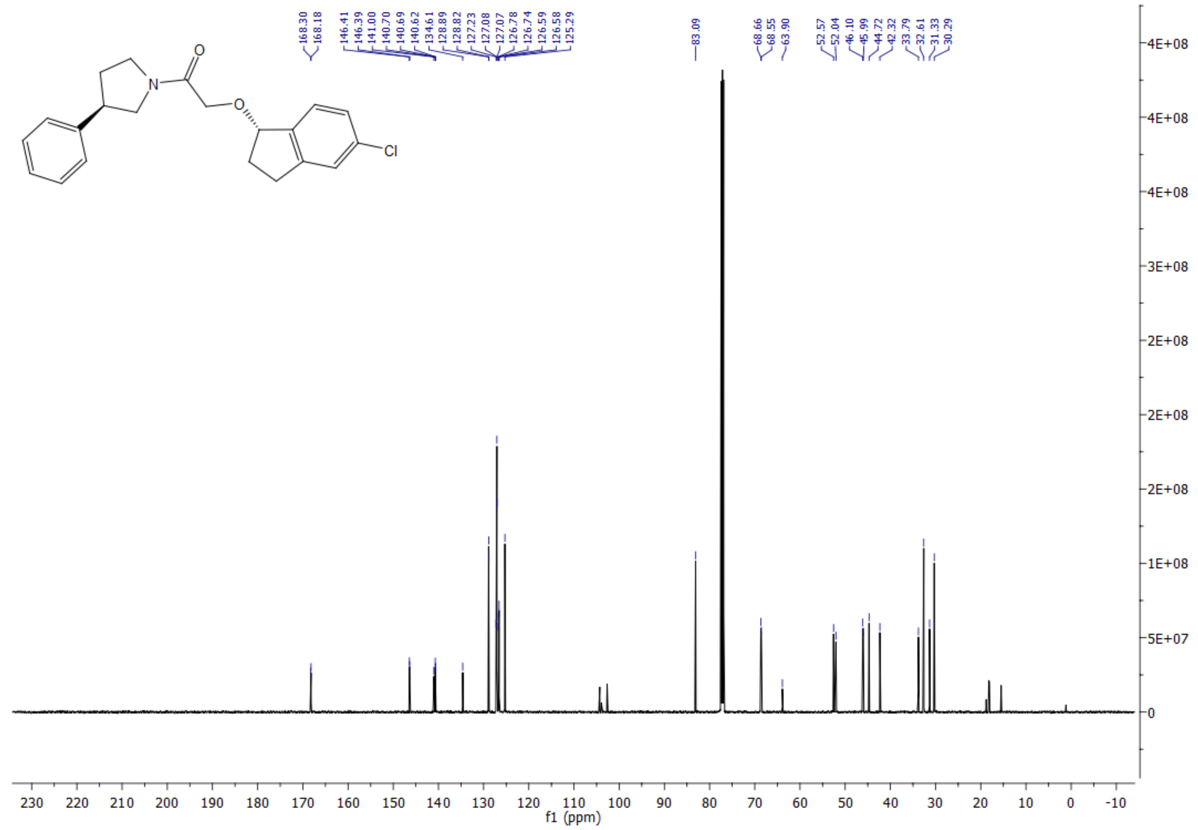

P2b

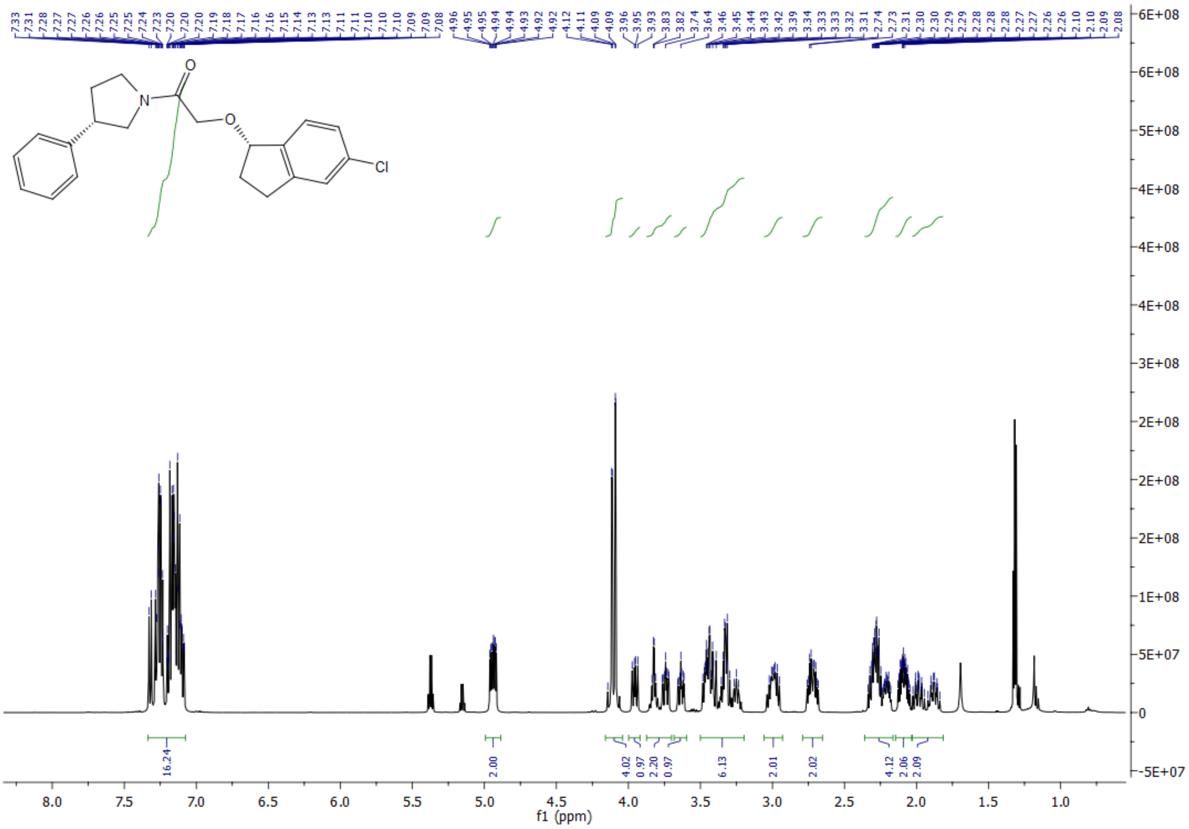

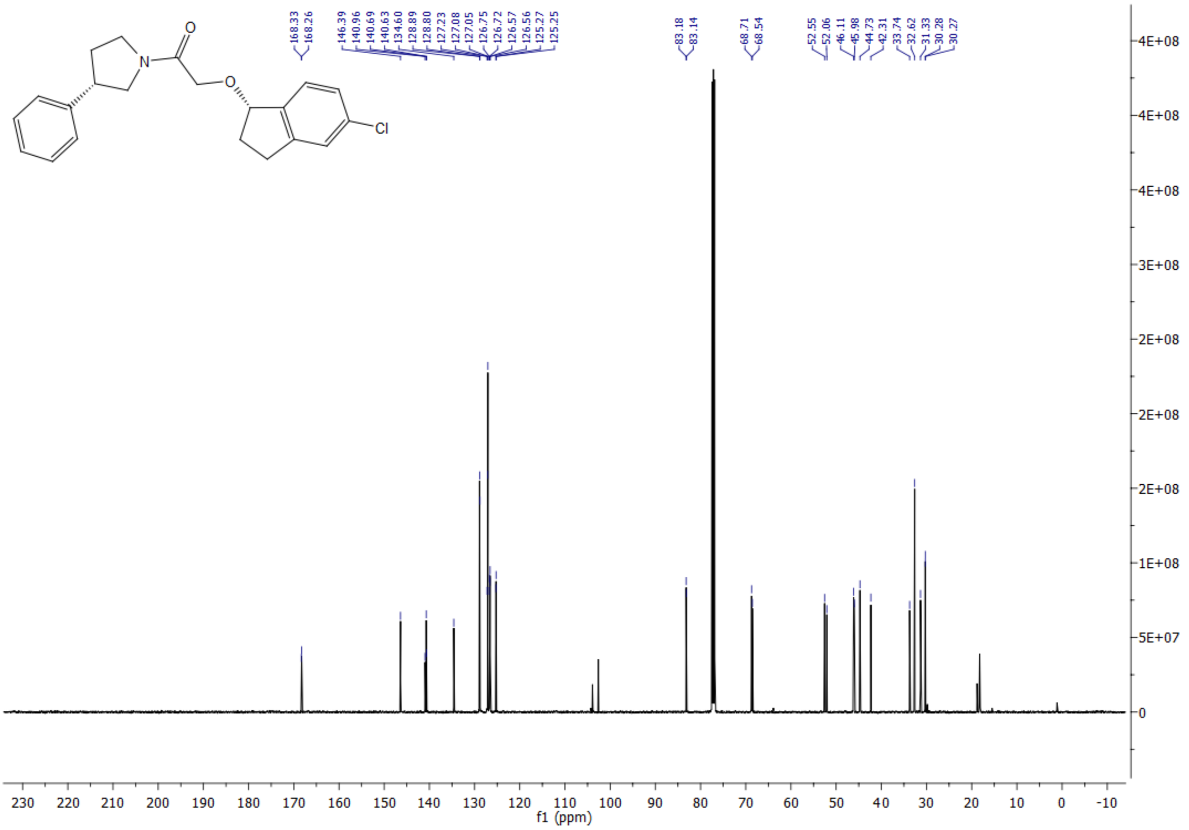

ent-**P2b**

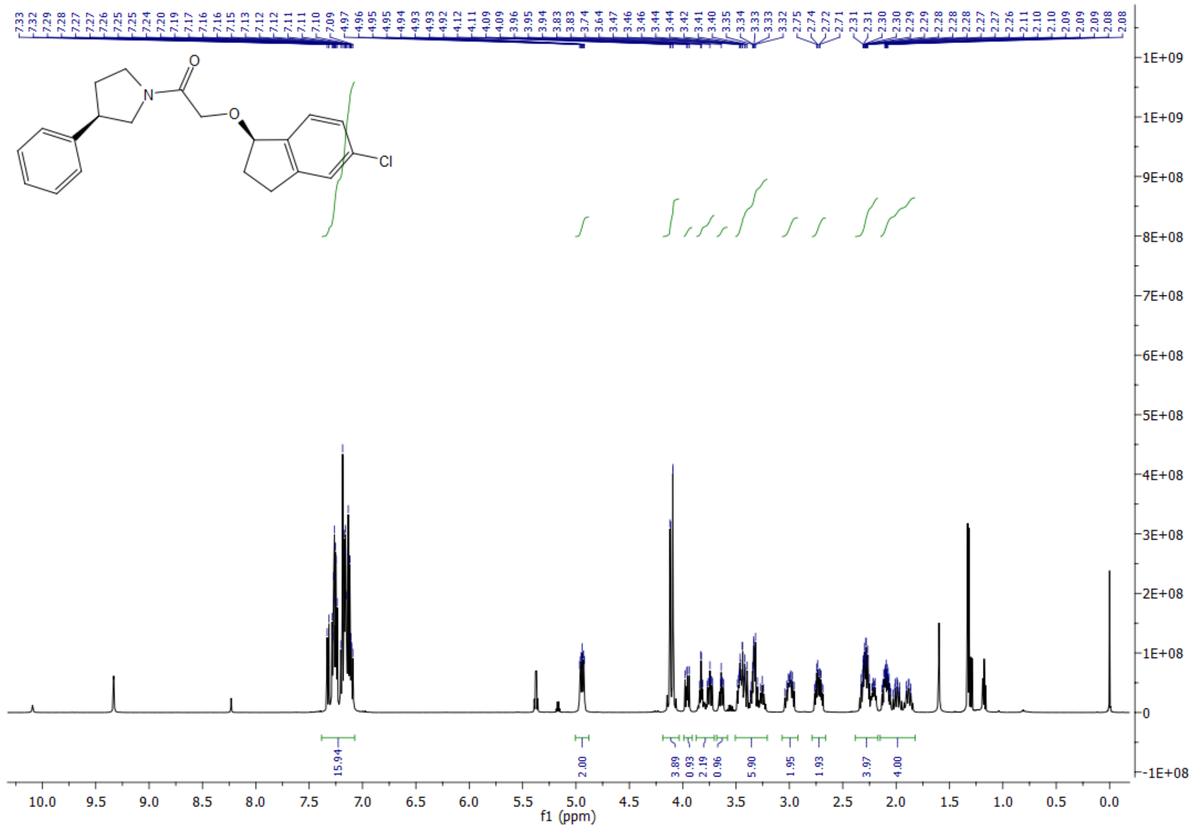

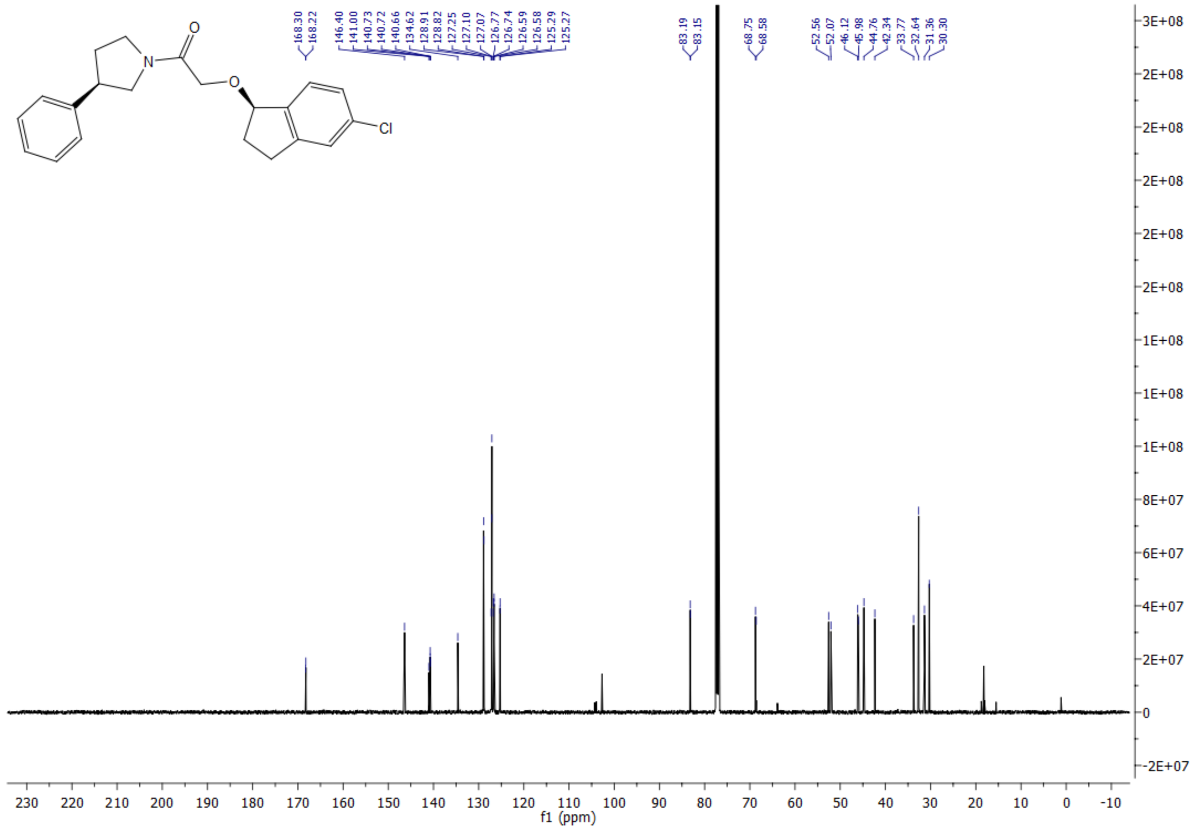

P2b vs *ent*-P2b

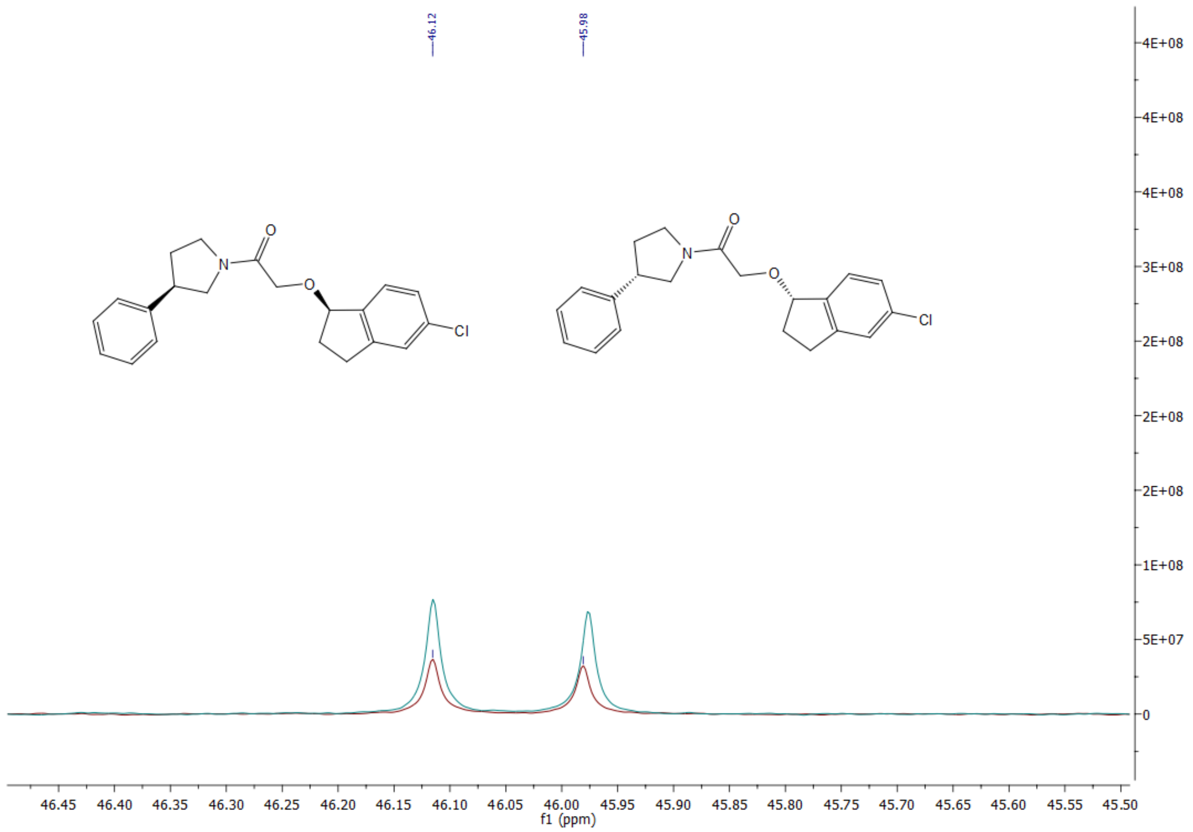

ent-**P2a vs** *ent*-**P2b**

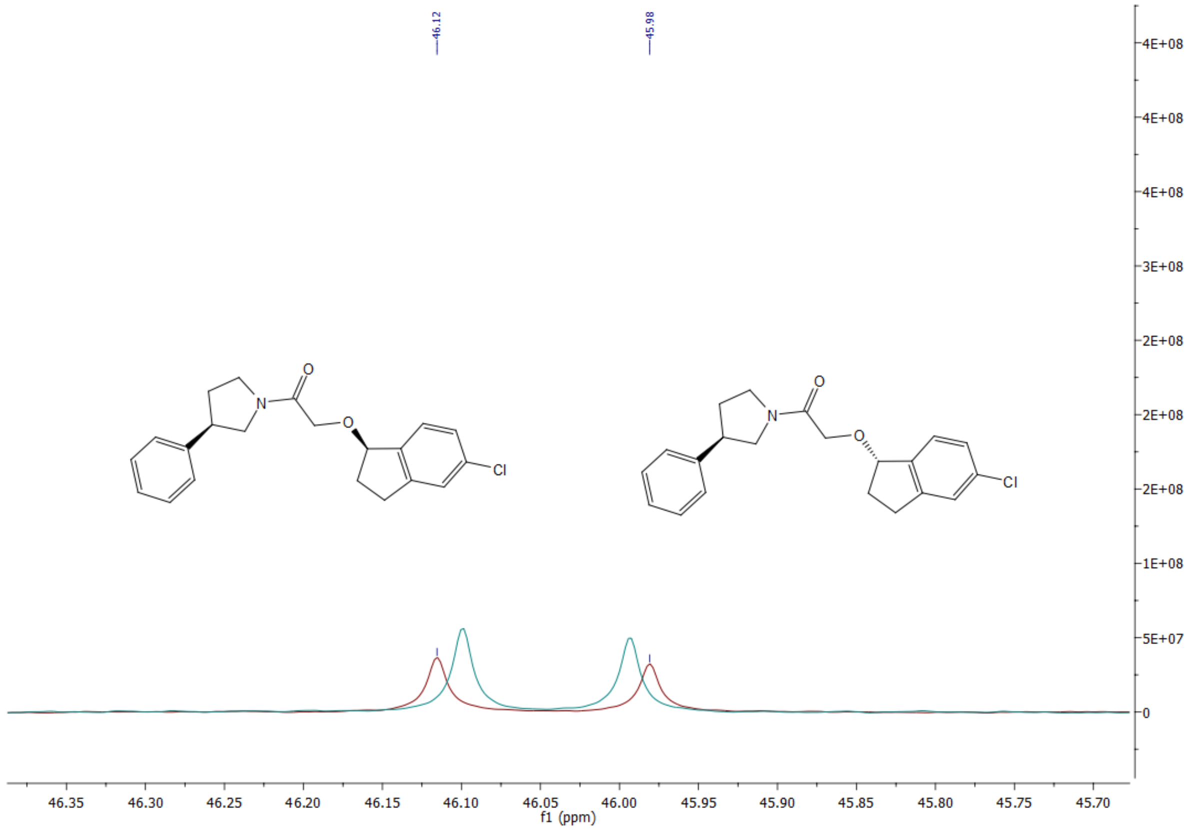

SI1

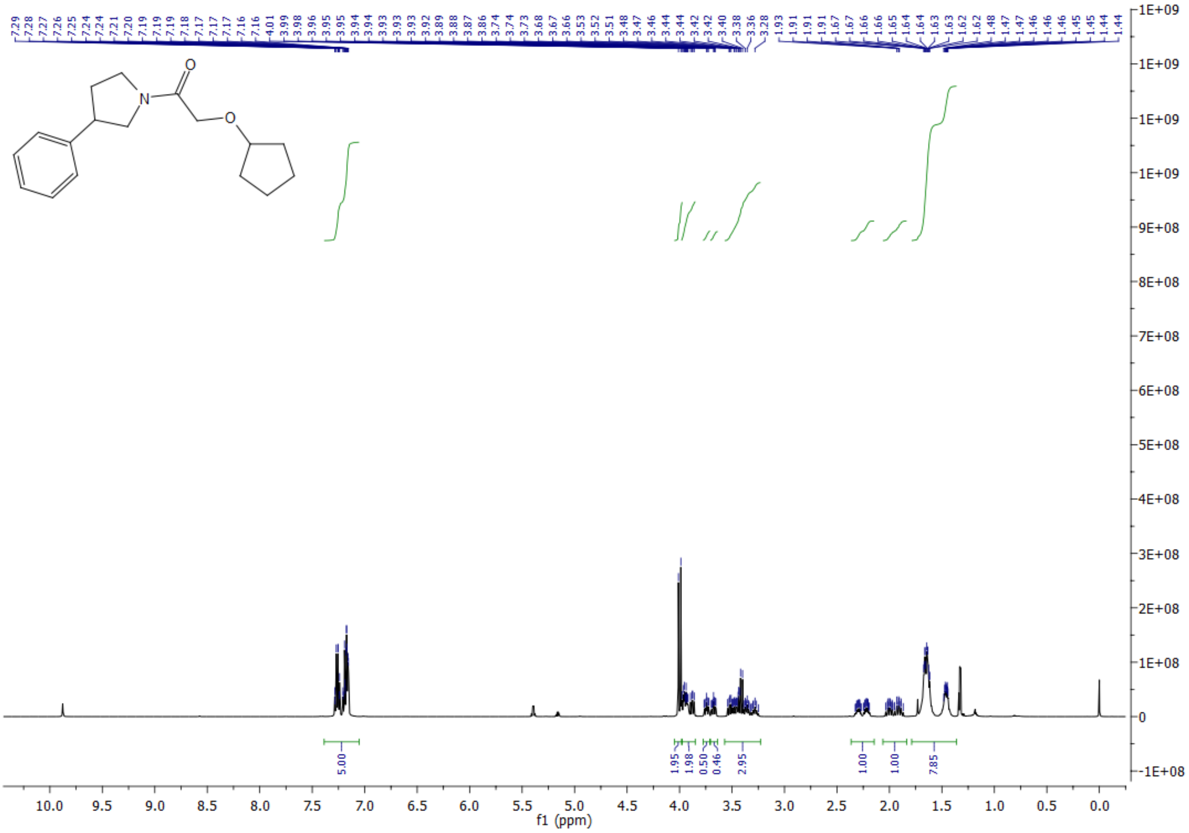

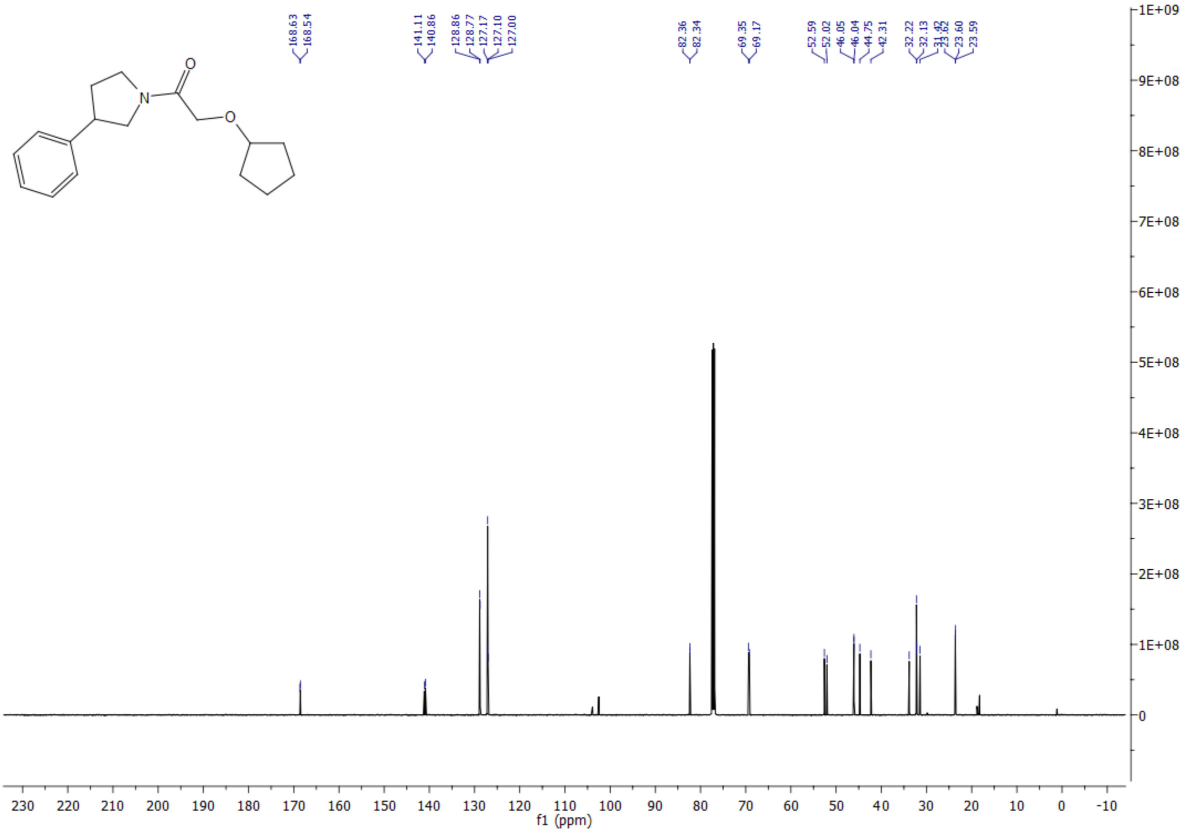

SI2

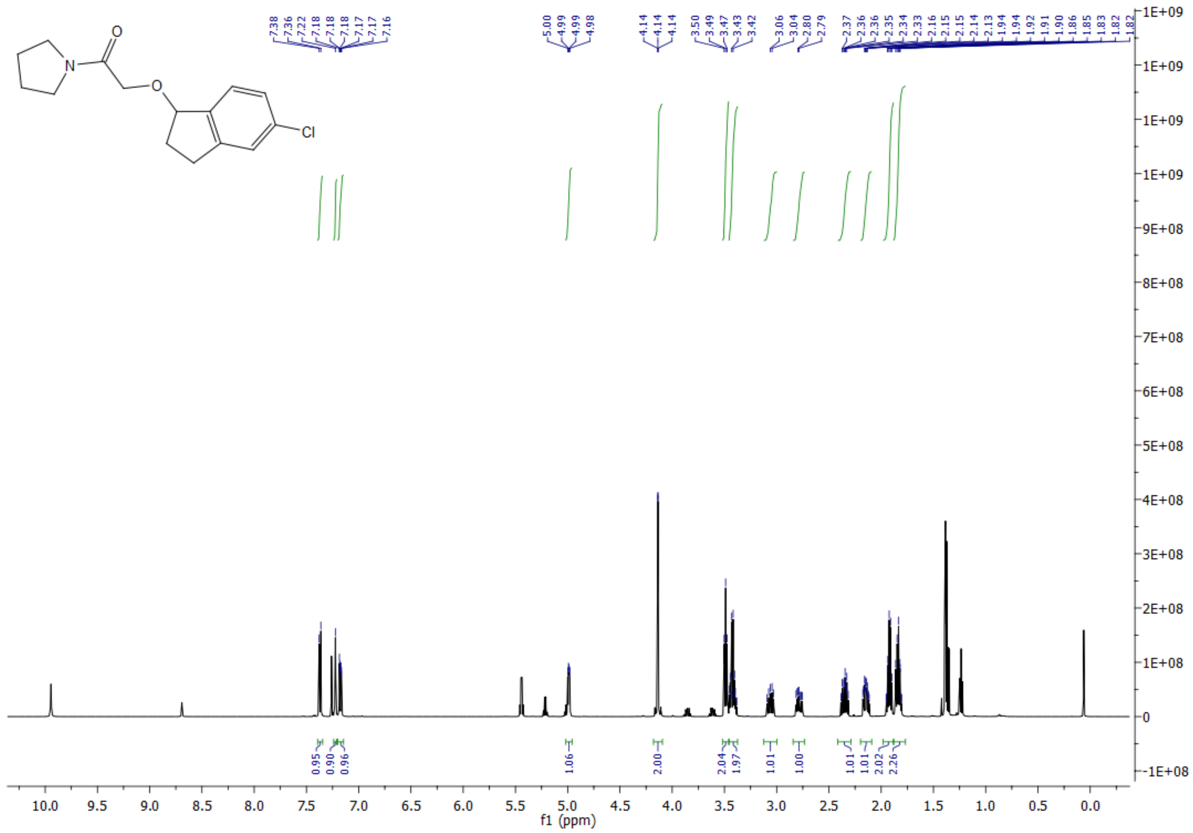
O
N
O
Cl
7.38
7.36
7.22
7.18
7.18
7.18
7.17
7.17
7.16
5.00
4.99
4.99
4.98
4.14
4.14
4.14
4.14
3.50
3.49
3.47
3.43
3.42
3.06
3.04
2.80
2.79
2.37
2.36
2.36
2.35
2.34
2.33
2.16
2.15
2.15
2.14
2.13
1.94
1.94
1.92
1.91
1.90
1.86
1.85
1.83
1.82
1.82
0.95
0.90
0.96
1.06
2.00
2.04
1.97
1.01
1.00
1.01
1.01
2.02
2.26
f1 (ppm)

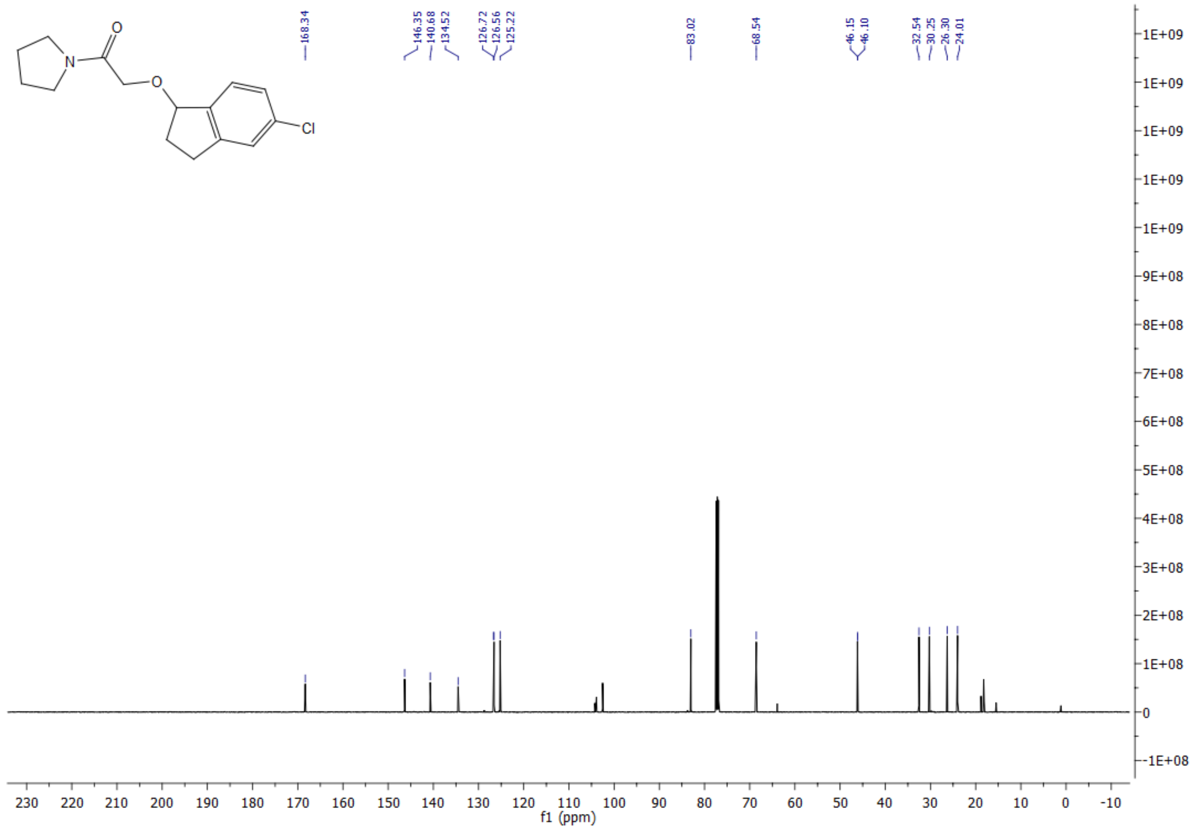
O
N
O
Cl
168.34
146.35
140.68
134.52
126.72
126.56
125.22
83.02
68.54
46.15
46.10
32.54
30.25
26.30
24.01
f1 (ppm)

P1

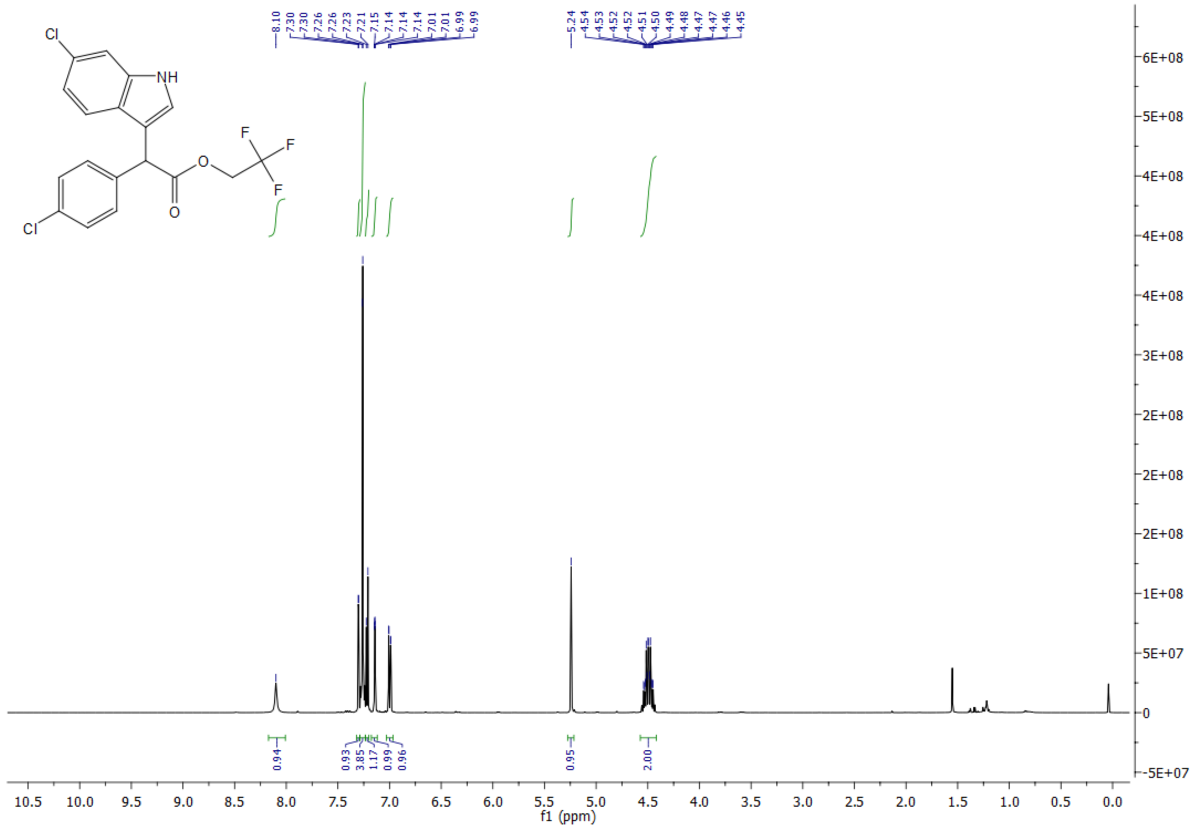
8.10
7.30
7.30
7.26
7.26
7.23
7.21
7.15
7.14
7.14
7.14
7.01
7.01
6.99
6.99
5.24
4.54
4.53
4.52
4.52
4.51
4.50
4.49
4.48
4.47
4.46
4.45
0.94
0.93
3.85
1.17
0.99
0.96
0.95
2.00
f1 (ppm)

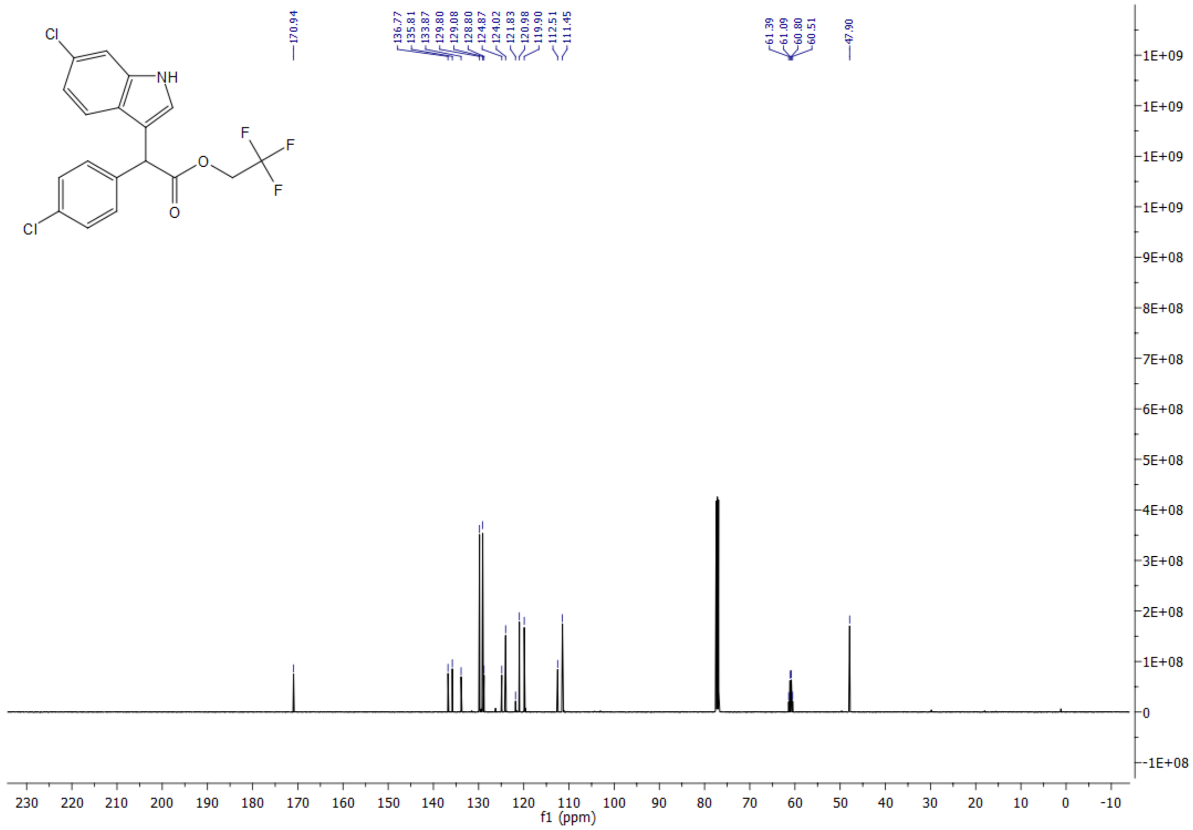
170.94
136.77
135.81
133.87
129.80
129.08
128.80
124.87
124.02
121.83
120.98
119.90
112.51
111.45
61.39
61.09
60.80
60.51
47.90
f1 (ppm)

P6

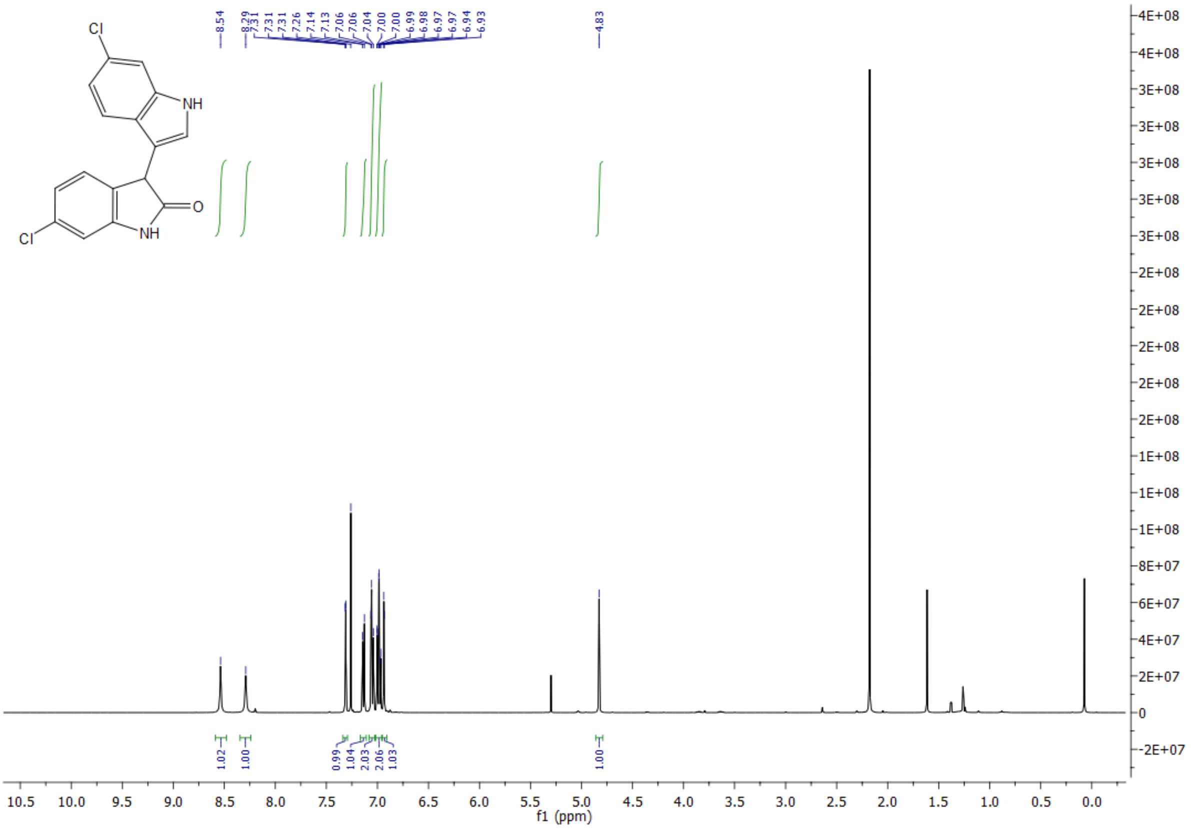
Cl
NH
O
NH
Cl
8.54
8.29
7.31
7.31
7.31
7.26
7.14
7.13
7.06
7.06
7.04
7.00
7.00
6.99
6.98
6.97
6.97
6.94
6.93
4.83
1.02
1.00
0.99
1.04
2.03
2.06
1.03
1.00
f1 (ppm)

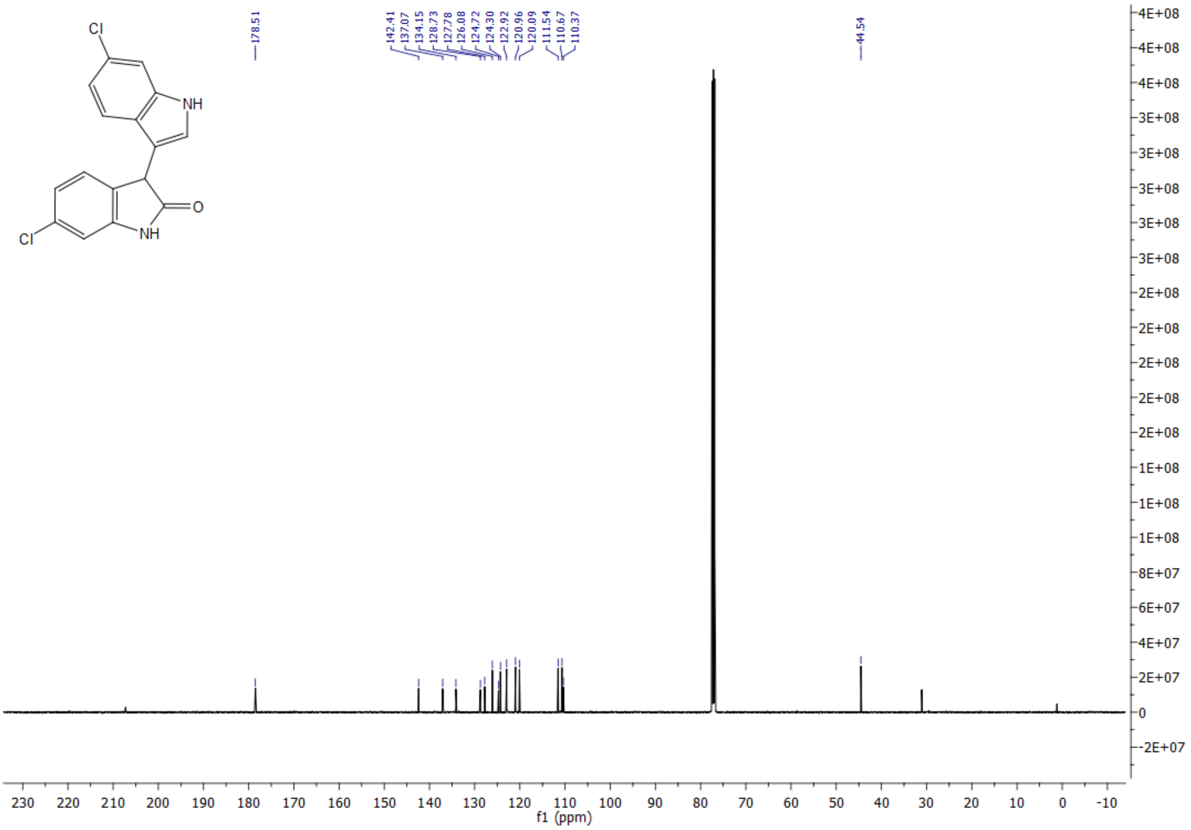
Cl
NH
O
NH
Cl
178.51
142.41
137.07
134.15
128.73
127.78
126.08
124.72
124.30
122.92
120.96
120.09
111.54
110.67
110.37
44.54
f1 (ppm)

SI3

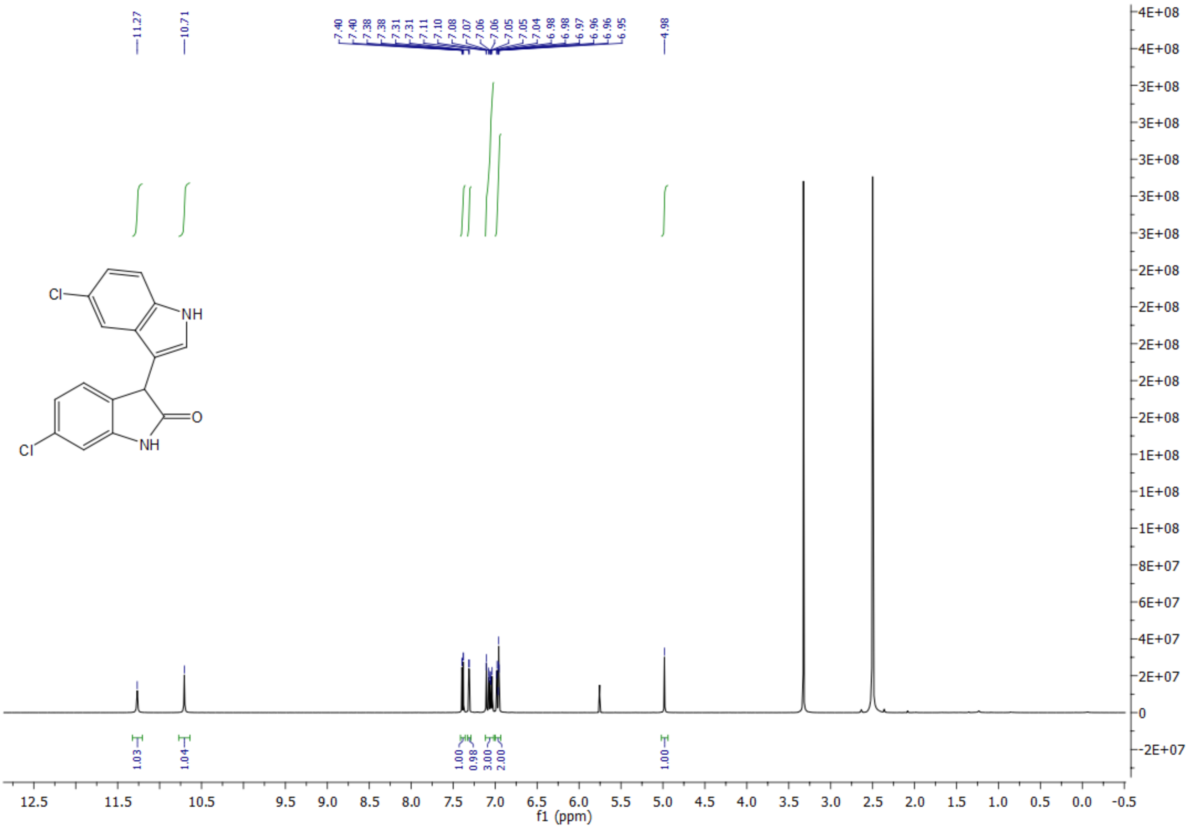

11.27
10.71
7.40 7.40 7.38 7.38 7.31 7.31 7.11 7.10 7.08 7.07 7.06 7.06 7.05 7.05 7.04 6.98 6.98 6.97 6.96 6.96 6.95
4.98
Cl
NH
O
Cl
NH
1.03
1.04
1.00 0.98 3.00 2.00
1.00
f1 (ppm)

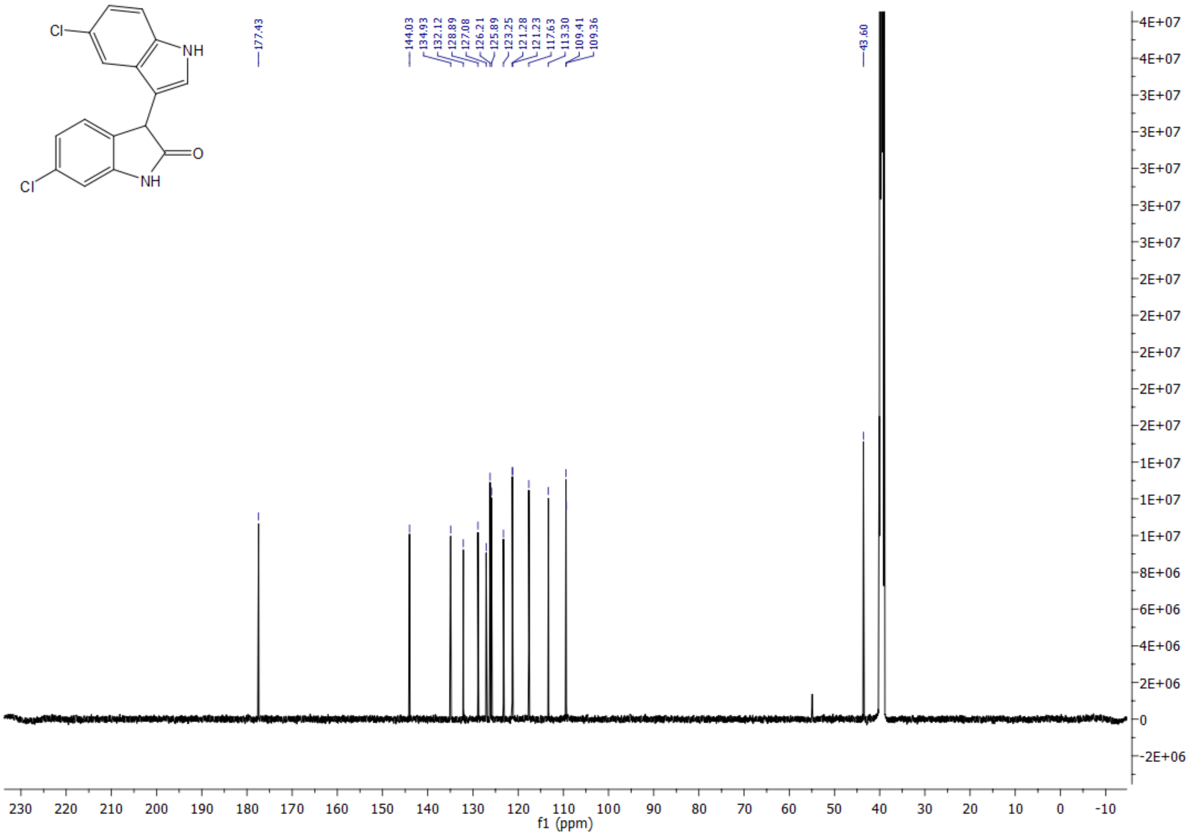

Cl
NH
O
Cl
NH
177.43
144.03 134.93 132.12 128.89 127.08 126.21 125.89 123.25 121.28 121.23 117.63 113.30 109.41 109.36
43.60
f1 (ppm)

P5

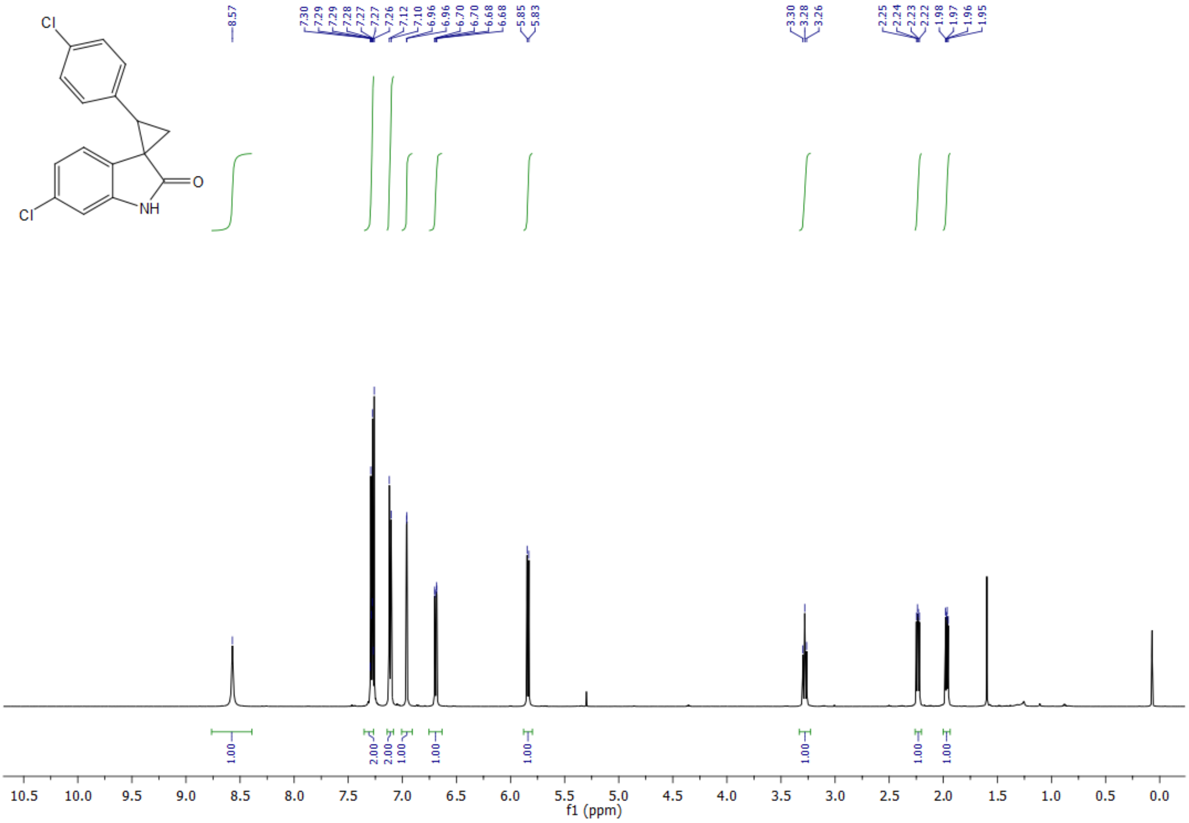
Cl
Cl
NH
O
8.57
7.30 7.29 7.29 7.28 7.27 7.27 7.26 7.12 7.10 6.96 6.96 6.70 6.70 6.68 6.68 5.85 5.83
3.30 3.28 3.26
2.25 2.24 2.23 2.22 1.98 1.97 1.96 1.95
1.00 2.00 2.00 1.00 1.00 1.00 1.00 1.00 1.00
f1 (ppm)

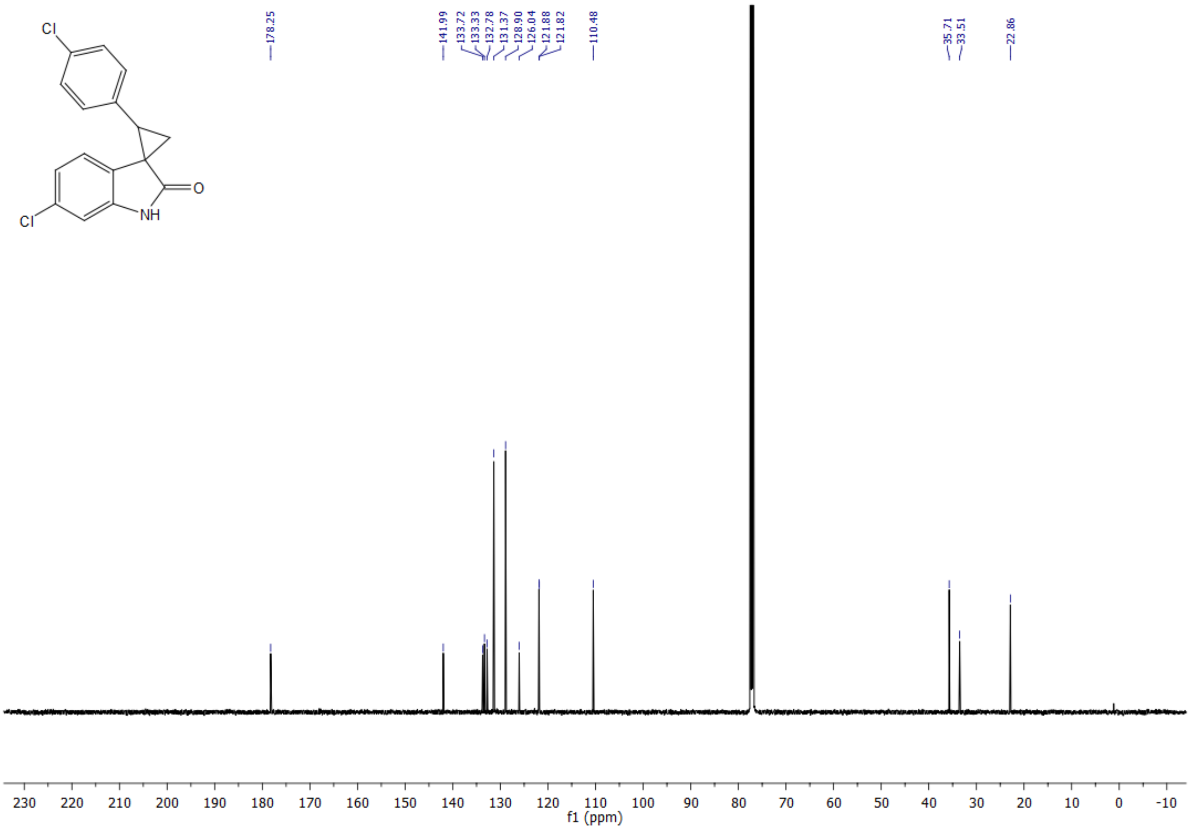
Cl
Cl
NH
O
178.25
141.99 133.72 133.33 132.78 131.37 128.90 126.04 121.88 121.82
110.48
35.71 33.51
22.86
f1 (ppm)

SI4

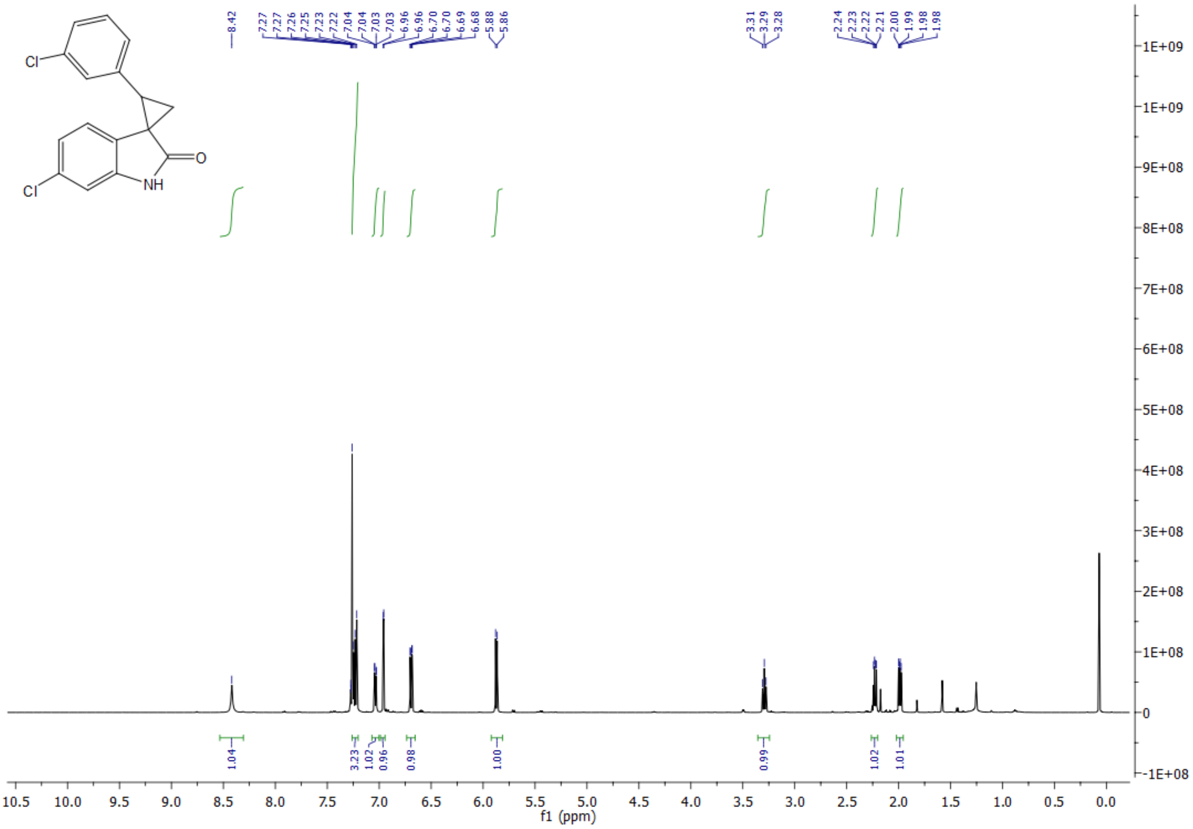
8.42
7.27 7.27 7.26 7.25 7.23 7.22 7.22 7.04 7.03 7.03 6.96 6.96 6.70 6.70 6.69 6.68 5.88 5.86
3.31 3.29 3.28
2.24 2.23 2.22 2.21 2.00 1.99 1.98 1.98
1.04 3.23 1.02 0.96 0.98 1.00 0.99 1.02 1.01
f1 (ppm)

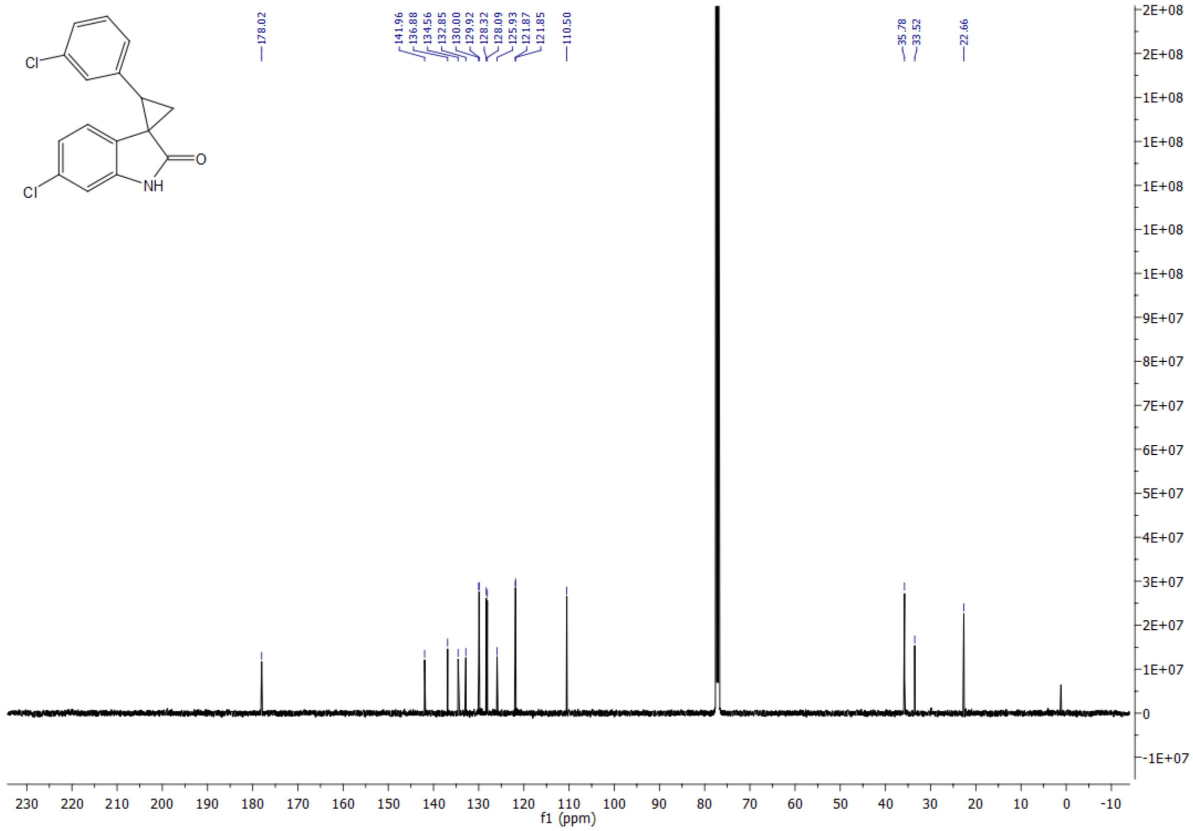
178.02
141.96 136.88 134.56 132.85 130.00 129.92 128.32 128.09 125.93 121.87 121.85
110.50
35.78 33.52
22.66
f1 (ppm)

P3

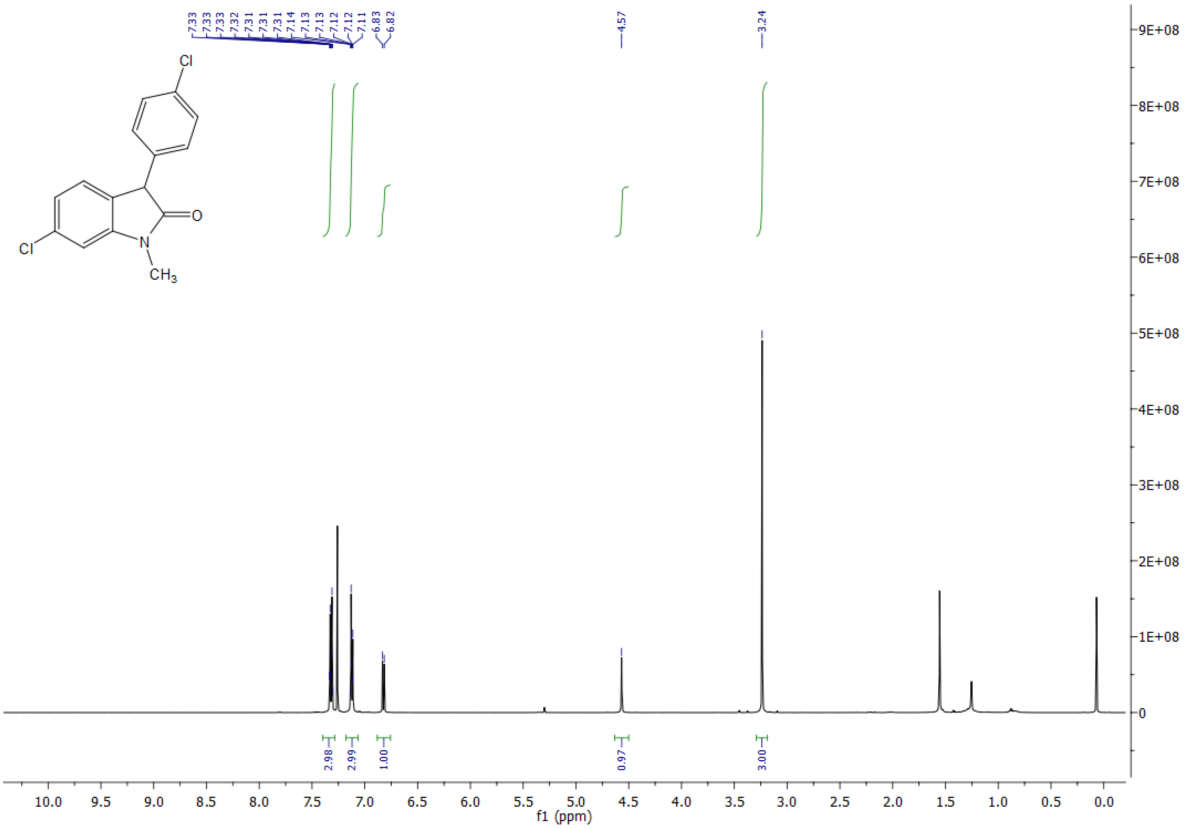
7.33
7.33
7.33
7.32
7.31
7.31
7.31
7.14
7.13
7.13
7.12
7.12
7.11
6.83
6.82
4.57
3.24
2.98
2.99
1.00
0.97
3.00
f1 (ppm)

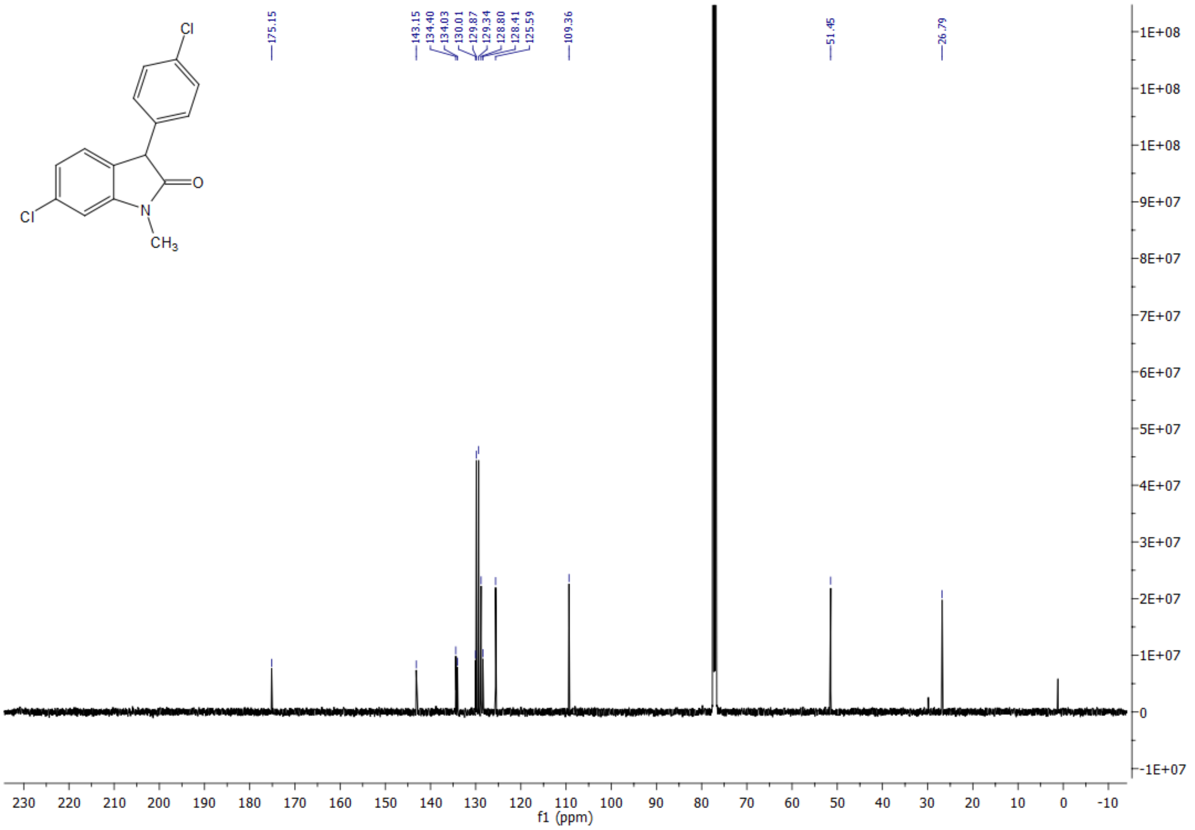
175.15
143.15
134.40
134.03
130.01
129.87
129.34
128.80
128.41
125.59
109.36
51.45
26.79
f1 (ppm)

P4

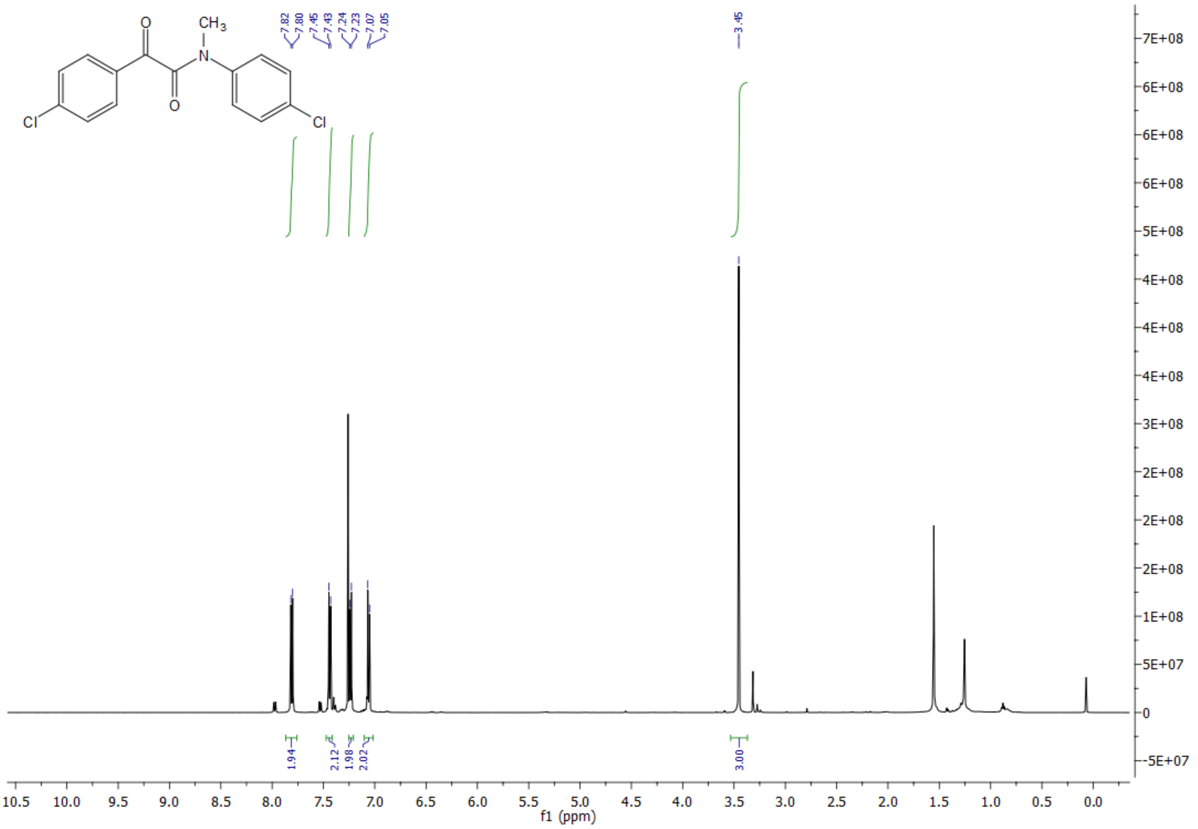

7.82
7.80
7.45
7.43
7.24
7.23
7.07
7.05
3.45
1.94
2.12
1.98
2.02
3.00
f1 (ppm)

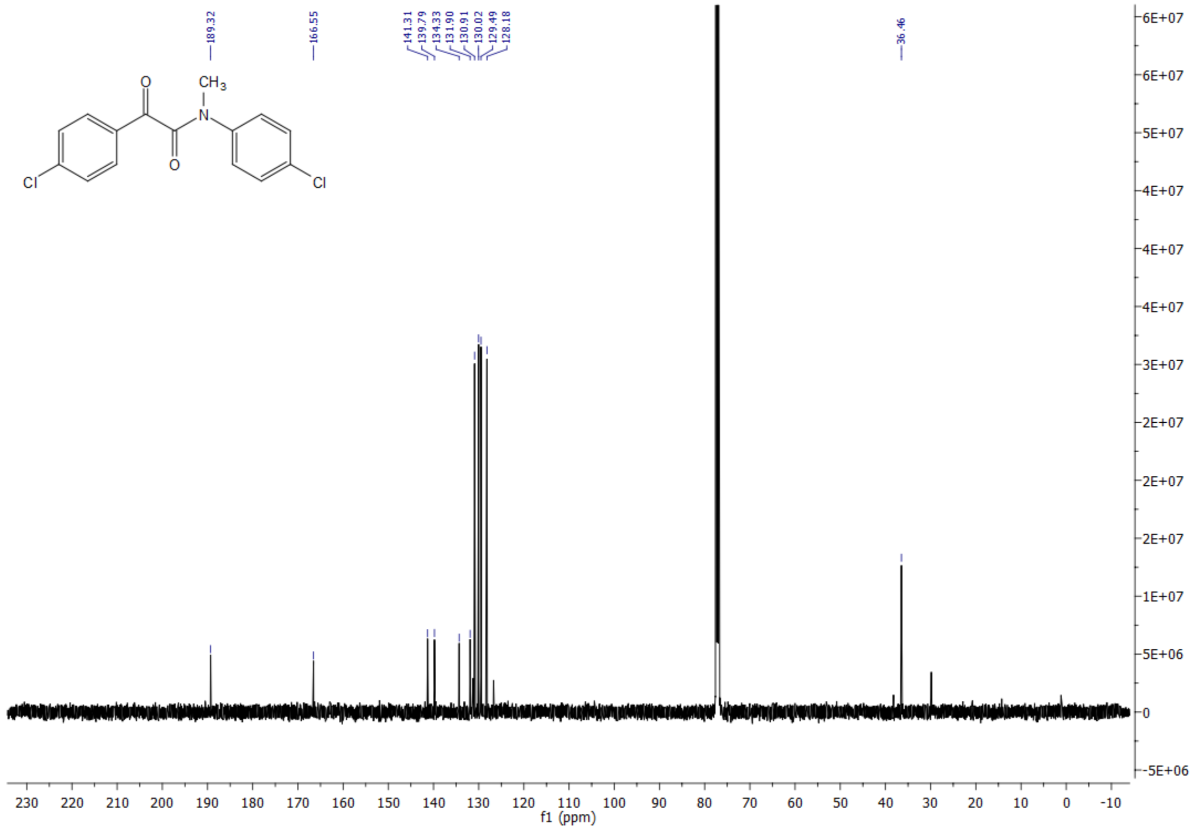

189.32
166.55
141.31
139.79
134.33
131.90
130.91
130.02
129.49
128.18
36.46
f1 (ppm)

P7

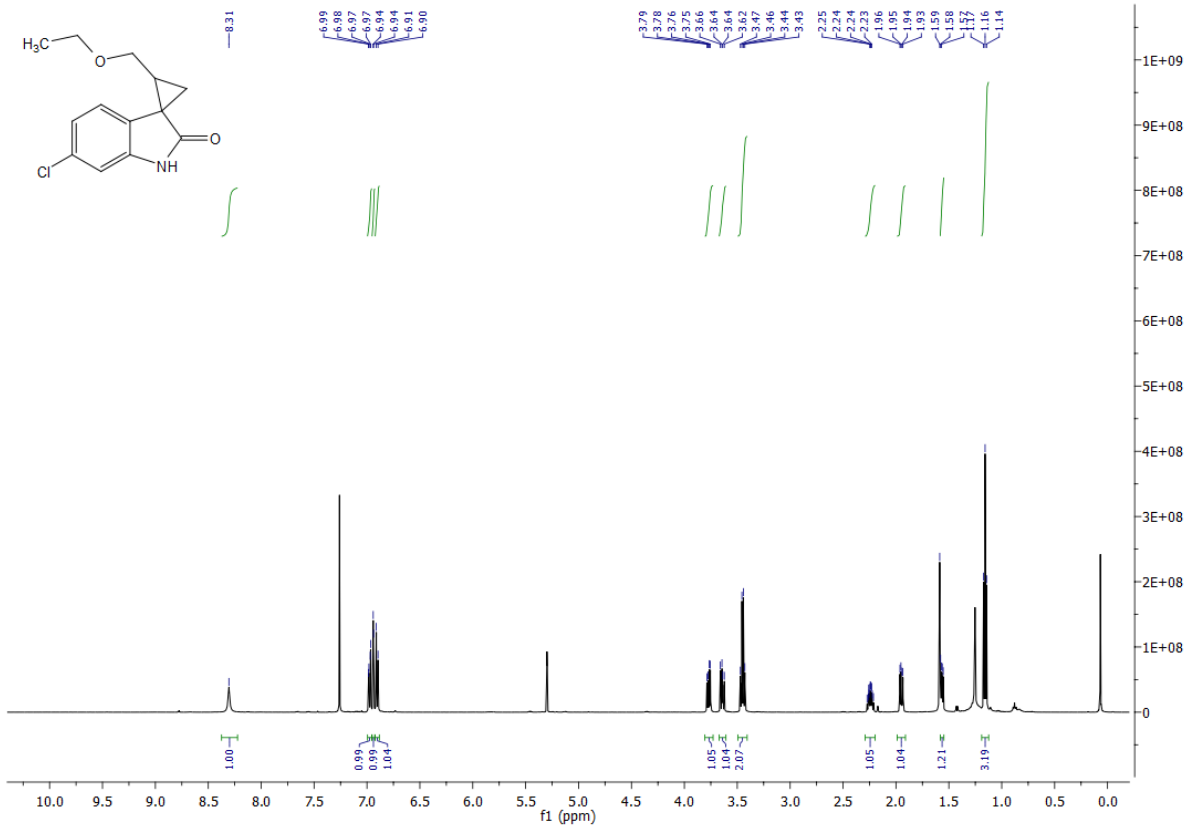

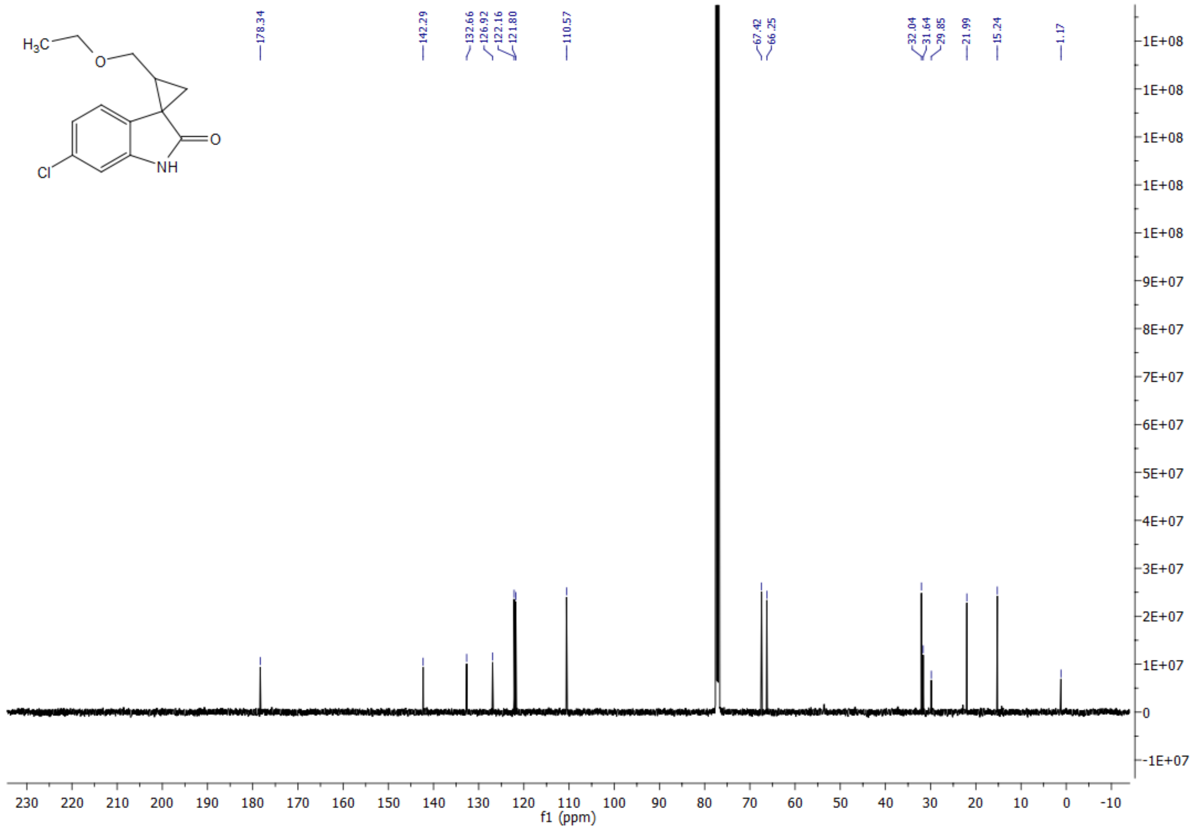

10. References

[1] (a) J. P. Plante, T. Burnley, B. Malkova, M. E. Webb, S. L. Warriner, T. A. Edwards and A. J. Wilson, *Chem. Commun*., 2009, 5091–5093; (b) F. Campbell, J. P. Plante, T. A. Edwards, S. L. Warriner and A. J. Wilson, *J. Org. Biomol. Chem.,* 2010, **8**, 2344-2351; (c) V. Azzarito, P. Prabhakaran, A. I. Bartlett, N. S. Murphy, M. J. Hardie, C. A. Kilner, T. A. Edwards, S. L. Warriner and A. J. Wilson, *Org. Biomol. Chem*., 2012, **10**, 6469-6472; (d) R. Fasan, R. L. Dias, K. Moehle, O. Zerbe, J. W. Vrijbloed, D. Obrecht, J. A. Robinson, *Angew. Chem. Int. Ed*., 2004, **43**, 2109-2112; (e) A. A. Ibarra, G. J. Bartlett, Z. Hegedüs, S. Dutt, F. Hobor, K. A. Horner, K. Hetherington, K. Spence, A. Nelson, T. A. Edwards, D. N. Woolfson, R. B. Sessions and A. J. Wilson, *ACS Chem. Biol.,* 2019, **14**, 2252–2263; (f) A. Barnard, K. Long, D. J. Yeo, J. A. Miles, V. Azzarito, G. M. Burslem, P. Prabhakaran, T. A. Edwards and A. J. Wilson, *Org. Biomol. Chem.,* 2014, 12, 6794-6799.

[2] S. Chow, A. I. Green, C. Arter, S. Liver, A. Leggott, L. Trask, G. Karageorgis, S. Warriner, A. Nelson, *Synthesis*, 2020, DOI: 10.1055/s-0039-1690905.

[3] B. Ma, Z. Chu, B. Huang, Z. Liu, L. Liu and J. Zhang, *Angew. Chem. Int. Ed*., 2017, **56**, 2749–2753.

[4] F. Varano, D. Catarzi, V. Colotta, F. R. Calabri, O. Lenzi, G. Filacchioni, A. Galli, C. Costagli, F. Deflorian and S. Moro, *Bioorganic and Medicinal Chemistry*, 2005, **13**, 5536 – 5549.

[5] L. T. Vassilev, B. T. Vu, B. Graves, D. Carvajal, F. Podlaski, Z. Filipovic, N. Kong, U. Kammlott, C. Lukacs, C. Klein, N. Fotouhi and E. A. Liu, *Science*, 2004, **303**, 844–848.

[6] Goddard, T. D. & Kneller, D. G. SPARKY 3. (University of California, 1997).

[7] Molecular Operating Environment (MOE); Chemical Computing Group, University of Sherbrooke: 1010 Sherbooke St. West, Suite #910, Montreal, QC, Canada, H3A 2R7, 2019.

[8] A. Z. Gonzalez, J. Eksterowicz, M. D. Bartberger, H. P. Beck, J. Canon, A. Chen, D. Chow, J. Duquette, B. M. Fox, J. Fu, X. Huang, J. B. Houze, L. Jin, Y. Li, Z. Li, Y. Ling, M. C. Lo, A. M. Long, L. R. McGee, J. McIntosh, D. L. McMinn, J. D. Oliner, T. Osgood, Y. Rew, A. Y. Saiki, P. Shaffer, S. Wortman, P. Yakowec, X. Yan, Q. Ye, D. Yu, X. Zhao, J. Zhou, S. H. Olson, J. C. Medina and D. Sun, *J. Med. Chem*., 2014, **57**, 2472–2488.

[9] S. Wang, W. Sun, Y. Zhao, D. McEachern, I. Meaux, C. Barrière, J. A. Stuckey, J. L. Meagher, L. Bai, L. Liu, C. G. Hoffman-Luca, J. Lu, S. Shangary, S. Yu, D. Bernard, A. Aguilar, O. Dos-Santos, L. Besret, S. Guerif, P. Pannier, D. Gorge-Bernat and L. Debussche, *Cancer Res.,* 2014, **74**, 5855–5865.

[10] RDKit: Open-source cheminformatics; http://www.rdkit.org
